# 1α,20*S*-Dihydroxyvitamin D_3_ Interacts with Vitamin D Receptor: Crystal Structure and Route of Chemical Synthesis

**DOI:** 10.1038/s41598-017-10917-7

**Published:** 2017-08-31

**Authors:** Zongtao Lin, Hao Chen, Anna Y. Belorusova, John C. Bollinger, Edith K. Y. Tang, Zorica Janjetovic, Tae-Kang Kim, Zhongzhi Wu, Duane D. Miller, Andrzej T. Slominski, Arnold E. Postlethwaite, Robert C. Tuckey, Natacha Rochel, Wei Li

**Affiliations:** 1 Department of Pharmaceutical Sciences, University of Tennessee Health Science Center, 881 Madison Avenue, Room 561, Memphis, TN 38163 United States; 2 Department of Integrative Structure Biology, IGBMC - CNRS UMR7104 – Inserm U964, 1, rue Laurent Fries, Illkirch, 67400 France; 3 Department of Structural Biology, St. Jude Children’s Research Hospital, Memphis, TN 38105 United States; 4 School of Chemistry and Biochemistry, University of Western Australia, Crawley, WA 6009 Australia; 5 Department of Dermatology, University of Alabama at Birmingham, Birmingham, AL 35294 United States; 6 VA Medical Center, Birmingham, AL 35294 United States; 7 Department of Medicine, University of Tennessee Health Science Center, Memphis, TN 38163 United States; 8 VA Medical Center, Memphis, TN 38104 United States; 9 Present Address: Department of Medicinal Chemistry, RIA iMed, AstraZeneca R&D, Pepparedsleden 1, S-431 83 Mölndal, Sweden

## Abstract

1α,20*S*-Dihydroxyvitamin D3 [1,20*S*(OH)_2_D_3_], a natural and bioactive vitamin D3 metabolite, was chemically synthesized for the first time. X-ray crystallography analysis of intermediate 15 confirmed its 1α-OH configuration. 1,20*S*(OH)_2_D_3_ interacts with the vitamin D receptor (VDR), with similar potency to its native ligand, 1α,25-dihydroxyvitamin D_3_ [1,25(OH)_2_D_3_] as illustrated by its ability to stimulate translocation of the VDR to the nucleus, stimulate VDRE-reporter activity, regulate VDR downstream genes (*VDR*, *CYP24A1*, *TRPV6* and *CYP27B1*), and inhibit the production of inflammatory markers (IFNγ and IL1β). However, their co-crystal structures revealed differential molecular interactions of the 20*S*-OH moiety and the 25-OH moiety to the VDR, which may explain some differences in their biological activities. Furthermore, this study provides a synthetic route for the synthesis of 1,20*S*(OH)_2_D_3_ using the intermediate 1α,3β-diacetoxypregn-5-en-20-one (3), and provides a molecular and biological basis for the development of 1,20*S*(OH)_2_D_3_ and its analogs as potential therapeutic agents.

## Introduction

**Figure 1:**
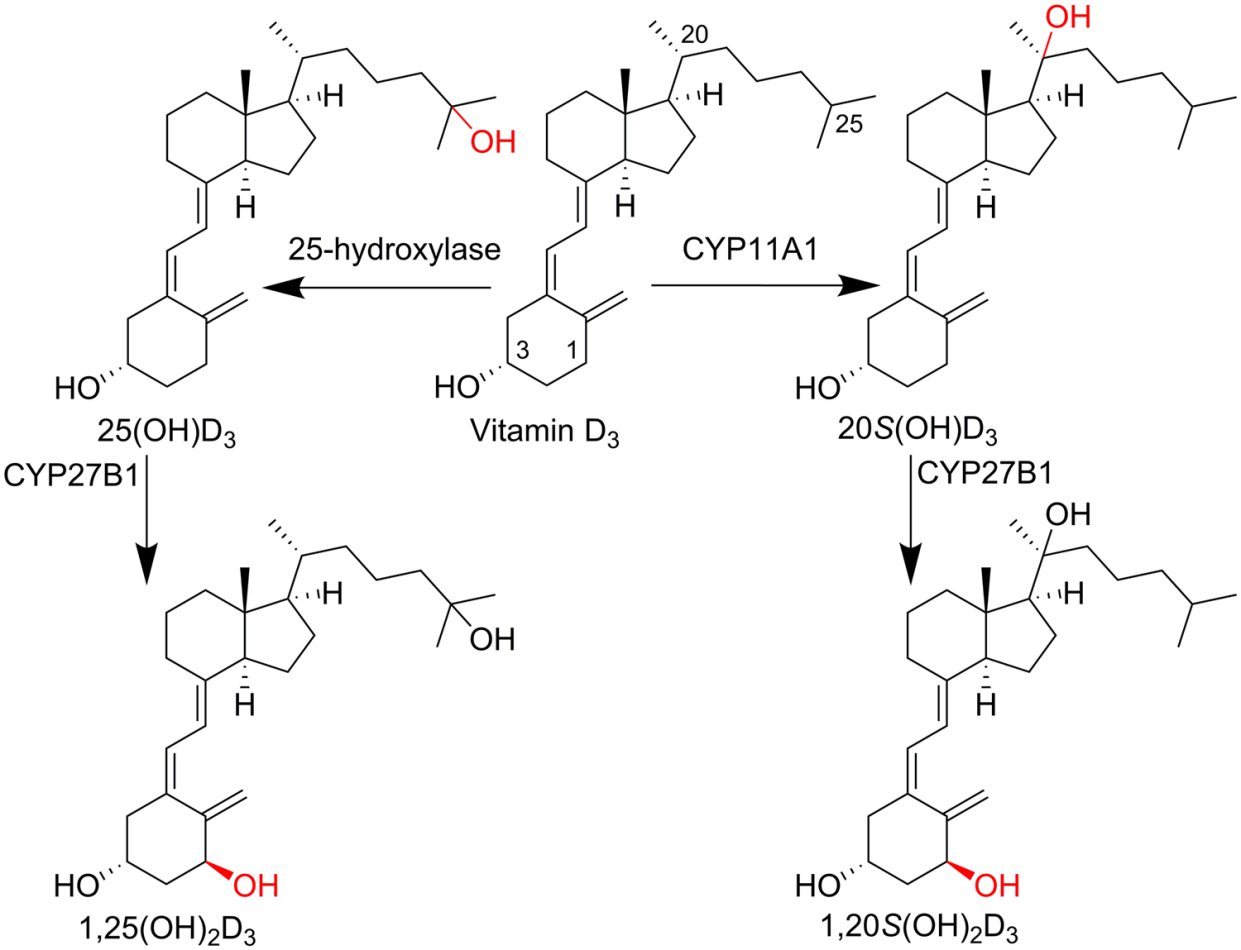
Classical (left) and novel (right) metabolic pathways of vitamin D_3_ activation.

The classical pathway of vitamin D_3_ (D_3_) activation involves two key steps: 25-hydroxylation to produce 25-hydroxyvitamin D_3_ [25(OH)D_3_], and 1α-hydroxylation by cytochrome CYP27B1 to produce the active 1α,25-dihydroxyvitamin D_3_ [1,25(OH)_2_D_3_] (Fig. 1)^1^. This natural ligand of the vitamin D receptor (VDR) regulates expressions of various genes including that encoding catabolic CYP24A1 through the VDR. Other activities mediated via the VDR include anti-inflammation, anti-proliferation, pro-differentiation, pro-apoptosis, immunomodulation, mineral homeostasis and anti-angiogenesis^2–4^. In addition, D_3_ can also be activated by a novel metabolic pathway initiated by CYP11A1 (P450scc) producing 20*S*-hydroxyvitamin D_3_ [20*S*(OH)D_3_] as the major product^5^. As an activation enzyme, CYP27B1 is able to hydroxylate 20*S*(OH)D_3_ producing the natural metabolite 1α,20*S*-dihydroxyvitamin D_3_ [1,20*S*(OH)_2_D_3_] and has been used to produce µg amounts of this product *in vitro* ^6, 7^. Alternative biosynthesis using CYP11A1 to 20*S*-hydroxylate commercially available 1α-hydroxyvitamin D_3_ [1(OH)D_3_] increased the production of 1,20*S*(OH)_2_D_3_ to 0.5–1 mg^7^.

1,20*S*(OH)_2_D_3_ has been found to upregulate the expression of CYP24A1 mRNA, suggesting that it can modulate the expression of genes downstream of the VDR^7^. It also inhibits cell growth and shows potent anti-leukemic and anti-melanoma activities, while displaying less calcemic (toxic) effect than 1,25(OH)_2_D_3_^7–10^. In addition, 1,20*S*(OH)_2_D_3_ was found in human epidermis suggesting an endogenous role in the skin^11^. However, the lack of detailed information on the interactions between 1,20*S*(OH)_2_D_3_ and VDR makes it difficult to understand its mechanism of action, and some of the differential effects of 1,20*S*(OH)_2_D_3_ and 1,25(OH)_2_D_3_. Here we present the high resolution X-ray crystal structure of 1,20*S*(OH)_2_D_3_ in complex with the VDR, as well as further characterization of its biological activities. Importantly, while 1,20*S*(OH)_2_D_3_ has great potential as a therapeutic agent, the production of 1,20*S*(OH)_2_D_3_^6, 7^ and its analogs^12, 13^ has been limited to date by the need for purified enzymes, CYP27B1 or CYP11A1, for their biosynthesis. Now we report the first chemical synthesis of 1,20*S*(OH)_2_D_3_ facilitating its production for further testing of its biological activities.

## Results and Discussion

### Retrosynthesis of 1,20*S*(OH)_2_D_3_

**Figure 2:**
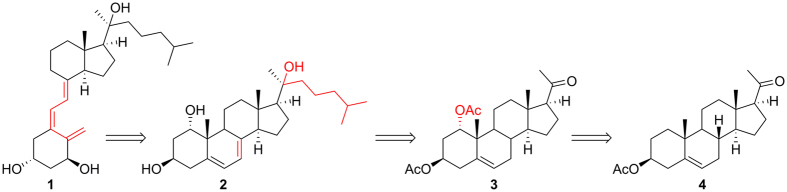
Retrosynthesis of 1,20*S*(OH)_2_D_3_.

A retrosynthetic strategy including a common 1α-OH intermediate **3** was proposed (Fig. 2). The D_3_-like structure could be obtained from UVB transformation of **2** ^12–14^, of which the 20*S*-OH and side chain could be achieved by Grignard reaction of **3** ^12–15^. Introduction of 1α-OH to **4** could be carried out by a multi-step conversion following the synthesis of androstenolone^16^.

### Synthesis of 1,20*S*(OH)_2_D_3_

**Figure 3:**
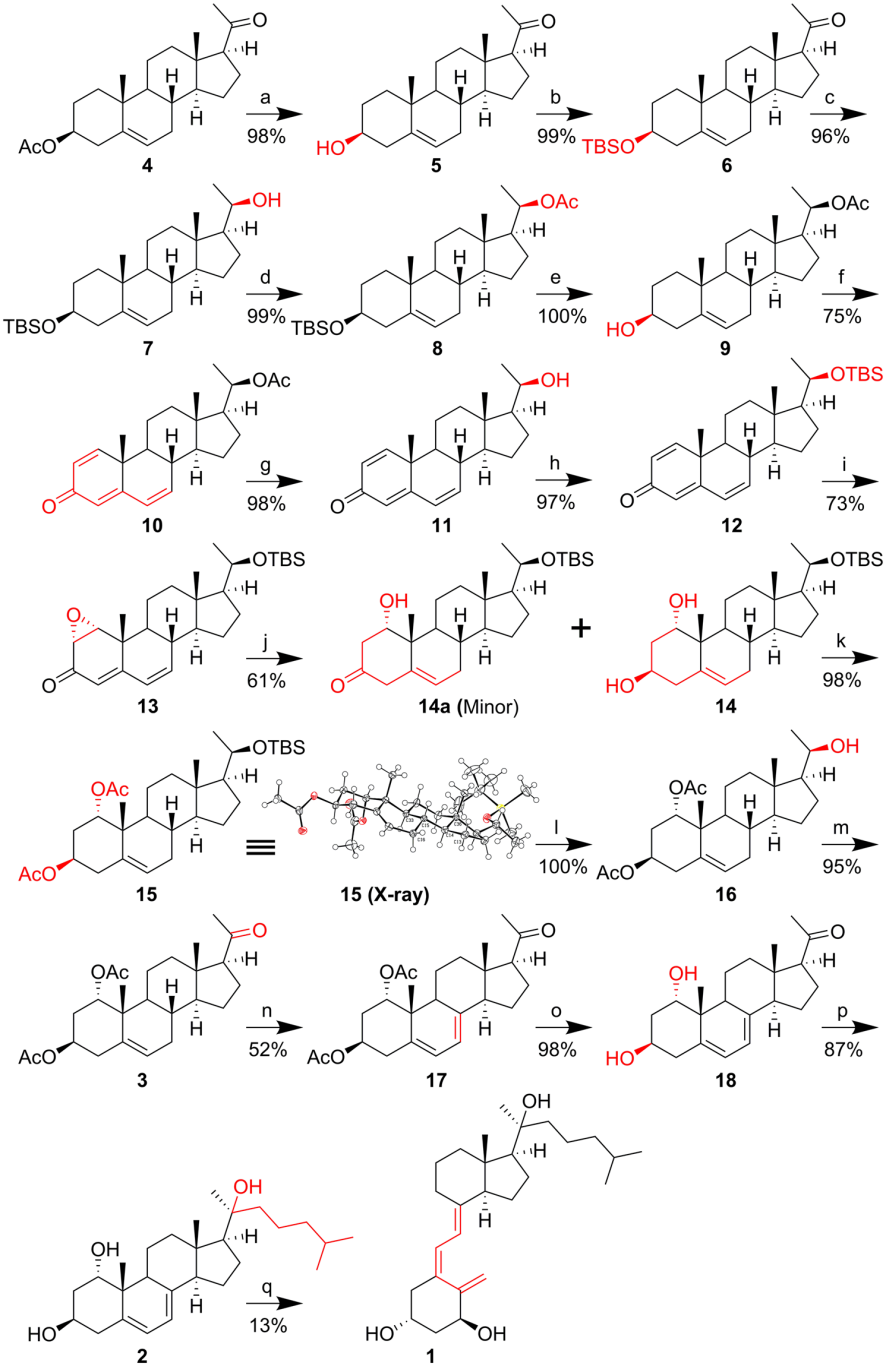
Synthesis of 1α,20*S*-dihydroxyvitamin D_3_. Reagents and conditions: (**a**) K_2_CO_3_, MeOH, r.t., overnight. (**b**) TBSCl, imidazole, DMF, r.t., overnight. (**c**) NaBH_4_, DCM: MeOH (1:1), 0 °C - r.t., overnight. (d) Ac_2_O, Et_3_N, DMAP, DCM, r.t., overnight. (e) TBAF, THF, r.t., 12 h. (**f**) DDQ, 1,4-dioxane, reflux, 4 h. (**g**) KOH, MeOH, r.t., 3 h. (**h**) TBSCl, imidazole, DMF, r.t., overnight. (**i**) KOH in MeOH, 30% H_2_O_2_, MeOH, −40 °C – 0 °C, 12 h. (**j**) Li, NH_3_ (liquid), −80 °C, 30 min; addition of starting material in THF, −80 °C, 2 h; −40 °C, 1 h; NH_4_Cl, −80 °C, 2 h. (k) Ac_2_O, Et_3_N, DMAP, DCM, r.t., overnight. (**l**) TBAF, THF, r.t., 48 h. (**m**) DMP, DCM, r.t., 12 h. (**n**) Dibromantin, AIBN, benzene: hexane (1:1), reflux 20 min; TBAB, THF, r.t., 75 min; TBAF, r.t., 50 min. (**o**) K_2_CO_3_, MeOH, r.t., overnight. (**p**) i) Mg, I_2_, 1-bromo-4-methylpentane, THF, reflux, 1 h; ii) **18**, THF, 0 °C – r.t., overnight. (**q**) UVB irradiation, Et_2_O, 50 °C, 15 min; r.t., 10 d; HPLC, MeCN:H_2_O. AIBN, azobisisobutyronitrile; DDQ, 2,3-dichloro-5,6-dicyanobenzoquinone; DMP, Dess–Martin periodinane; DMAP, 4-dimethylaminopyridine; HPLC, high-performance liquid chromatography; TBAB, tetra-*n*-butylammonium bromide; TBAF, tetra-*n*-butylammonium fluoride; TBSCl, *tert*-butyldimethylsilyl chloride.

The synthesis (Fig. 3) started with deacetylation and TBS protection of pregnenolone acetate (**4**) to give intermediate **6**. NaBH_4_ treatment of **6** selectively afforded the 20 *R* epimer as a major product which was then protected with an acetyl group to go through the DDQ oxidation safely (75% yield) to produce intermediate **10**. After replacing 20-OAc with 20-OTBS, a 1α,2α-epoxide group was introduced by adding KOH and H_2_O_2_ solution to afford intermediate **13** (73%), followed by Birch reduction to give 1α,3β-diol **14** (61%) as a major product^16–18^. To confirm the 1α-OH formation, **14** was protected with an acetyl group to produce **15**, which was characterized by 1D and 2D NMR spectrometry, and crystalized from hexane for X-ray structure analysis (see Supplementary Fig. S2). After removal of 20-OTBS, intermediate **16** was oxidized by DMP to 1α,3β-diacetoxypregn-5-en-20-one (**3**, 95%), which was then transformed into the 5,7-diene 7DHC intermediate (**17**, 52%) following a well-established procedure^12–14^. To avoid potential separation problems caused by acetyl protection after Grignard reaction, ester hydrolysis was carried out prior to Grignard reaction (87%) to afford 1α,20*S*-7DHC (**2**) where the formation of 20*S* confirmation was discussed in previous reports^12–14^. UVB irradiation of **2** in ethyl ether followed by pre-vitamin D_3_ isomerization afforded the desired product **1** (13%), which was compared with its enzymatic counterpart after HPLC separation.

Pregnenolone acetate (**4**) has often been used as the starting material for 20*S*(OH)D_3_ analogs^12–14^, in which 1α-hydroxylation was necessary to display potent stimulation of the VDR^12, 13^. Owing to the lack of appropriate 1α-OH intermediates, the production of 1α-OH derivatives of 20*S*(OH)D_3_ analogs was dependent on the purification of recombinant CYP27B1. The limited amount of 1α-OH derivatives that could be made was thus a hurdle for extensive biological testing. The production of 1α,3β-diacetoxypregn-5-en-20-one (**3**) in this report enables production of various analogs of 1,20*S*(OH)_2_D_3_ for future studies.

We experienced inconsistent yields during the Birch reduction of epoxide **13** in the initial trials. In fact, the addition of NH_4_Cl (a quenching step) is the key to the success of this reaction. Quick addition (<10 min) of NH_4_Cl gave predominantly intermediate **14a**, whereas slow addition (>2 h) afforded mainly the desired product **14**. To our knowledge, **14a** as a semi-reduced intermediate was obtained and characterized for the first time.

### HPLC showed matched retention times of chemical and enzymatic 1,20*S*(OH)_2_D_3_

**Figure 4:**
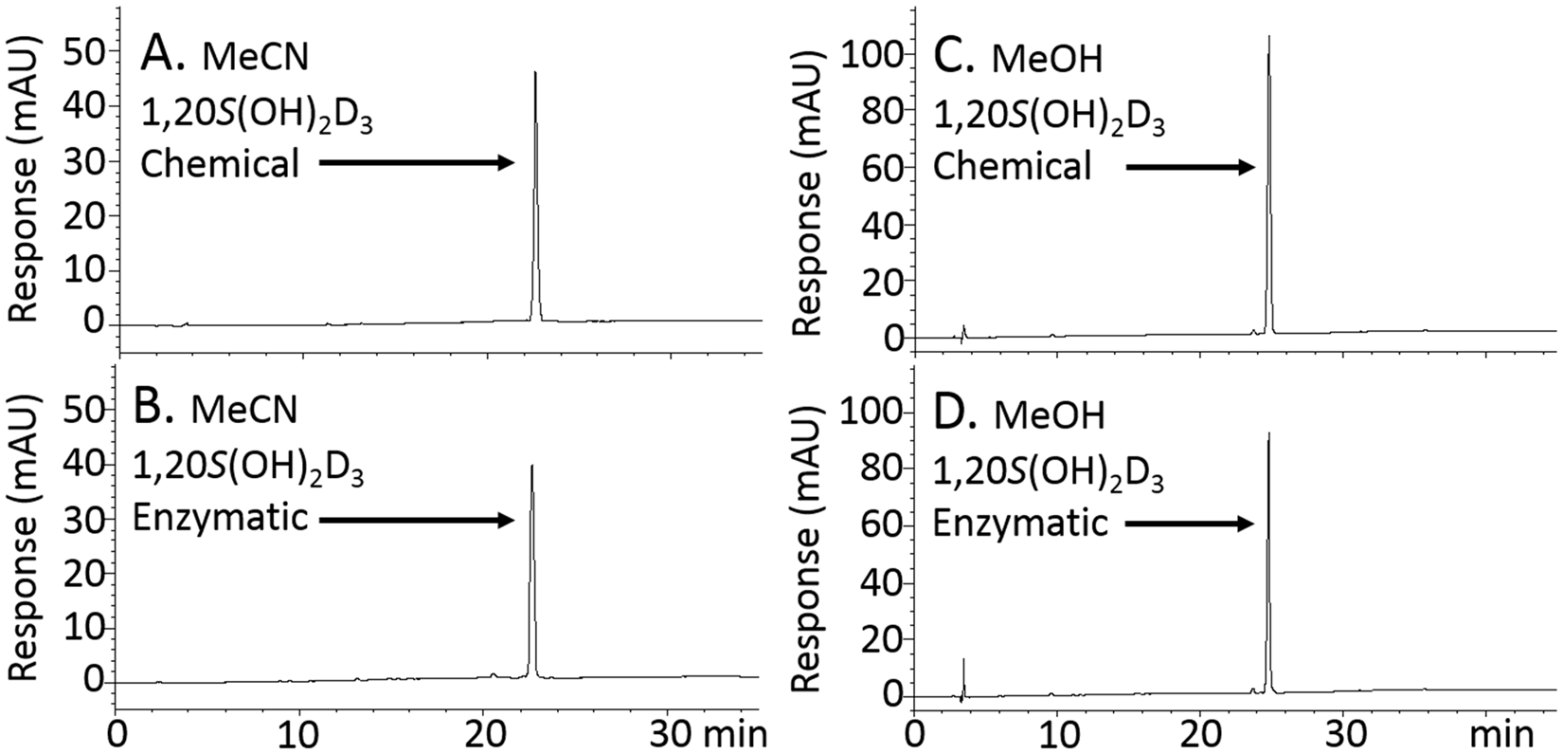
Comparison of HPLC retention times of chemical and enzymatic 1,20*S*(OH)_2_D_3_. Chemically synthesized (**A** and **C**) and enzymatically produced (**B** and **D**) 1,20*S*(OH)_2_D_3_ was analysed under MeCN: water condition (**A** and **B**, 0.25 µg) and MeOH: water condition (**C** and **D**, 0.5 µg).

In addition to the UV spectra and NMR identification (Supplementary Information), the chemically synthesized 1,20*S*(OH)_2_D_3_ was analysed by HPLC under two different solvent systems, either an acetonitrile in water gradient or a methanol in water gradient. We conclude that the chemically synthesized 1,20*S*(OH)_2_D_3_ and the enzymatically produced counterpart are identical on the basis of their UV and NMR spectra, as well as their HPLC retention times (Fig. 4). Co-migration of the chemically and enzymatically synthesized 1,20*S*(OH)_2_D_3_ was further confirmed by chromatography of a mixture of the two (see Supplementary Information).

### Identification of 15 as having a 1α-OH by NMR analysis

To identify the formation of the 1α-hydroxyl, the structure of intermediate **15** was characterized from its NMR spectra (Supplementary Information). The NOESY spectrum of **15** gave a strong NOE integral (0.42, see Supplementary Fig. S1) of 1H_β_ to 19-CH_3_, using the NOE integral of 1H_β_ to 2H_β_ as an internal reference. In contrast, the NOE signal of 1H_β_ to 2H_α_ was not observed, suggesting the presence of 1α-OAc group in **15**.

### Confirmation of 15 by X-ray crystallographic analysis

To confirm the structure of **15**, crystals were produced in hexane for X-ray crystallographic analysis (Supplementary Information). The X-ray structure of **15** (CCDC code: 1527430, Fig. 3) confirmed its absolute structure as the desired product reported in the Fig. 3.

### Transcriptional activity

The ability of 1,20*S*(OH)_2_D_3_ to activate the VDR was analysed in three cell lines (HaCaT, Caco-2 and Jurkat) transduced with a lentiviral vitamin D response element (VDRE) reporter (luciferase)^12–15^. Compared with 1,25(OH)_2_D_3_ and 22-oxa-1,25(OH)_2_D_3_ (22-Oxa), two known VDR agonists, 1,20*S*(OH)_2_D_3_ showed potent transcriptional activity with EC_50_s of 450 nM in HaCaT cells, 285 nM in Caco-2 cells and 19.1 nM in Jurkat cells (Table 1). Although less potent than 22-Oxa in all three cell lines, 1,20*S*(OH)_2_D_3_ is equally potent to (HaCaT and Caco-2 cells) or less potent than (Jurkat cells) 1,25(OH)_2_D_3_, the native ligand of the VDR.

**Table 1:** Stimulation of VDRE-reporter activity and inhibition of cytokine production by 1,20*S*(OH)_2_D_3_.

Compound	VDRE stimulation (nM)			Cytokine level	
	HaCaT	Caco-2	Jurkat	IFNγ	IL1β
Control	NA	NA	NA	710 ± 9	123 ± 2
1,20*S*(OH)_2_D_3_	450.4 ± 14.9	284.8 ± 13.2	19.1 ± 0.9	383 ± 3	90 ± 2
1,25(OH)_2_D_3_	421.9 ± 3.1	300.2 ± 9.2	2.1 ± 0.1	353 ± 11	121 ± 3
22-Oxa	10.5. ± 2.6	154.5 ± 0.8	1.2 ± 0.1	258 ± 2	91 ± 2

Note: VDRE stimulation activity = EC_50_ ± standard deviation, cytokine level in splenocyte cultures = value ± standard error of the mean (pg/mL).

### X-ray crystallographic analysis of the zVDR ligand binding domain in complex with 1,20*S*(OH)_2_D_3_

**Figure 5:**
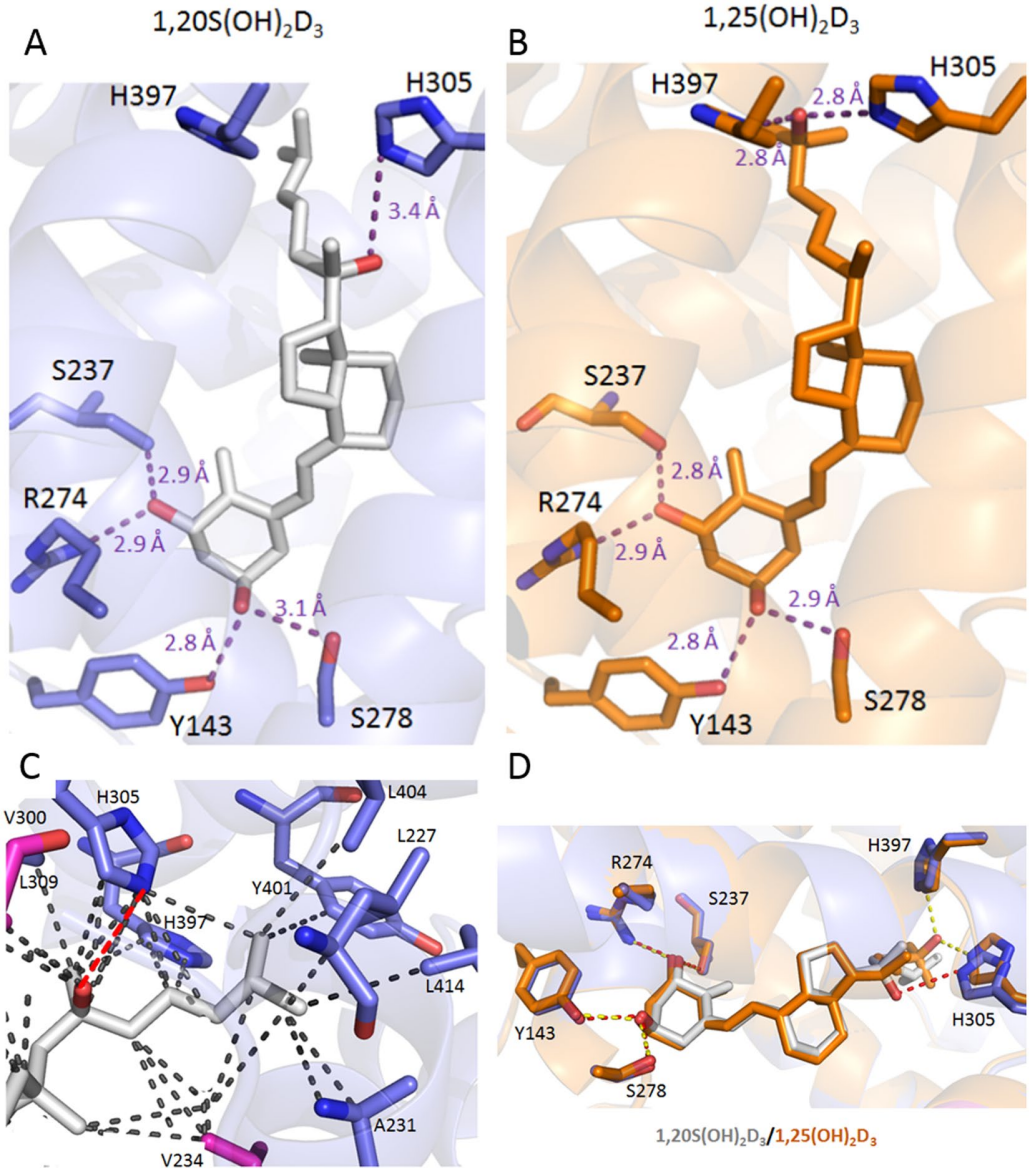
X-ray crystal structures of 1,20*S*(OH)_2_D_3_ and 1,25(OH)_2_D_3_ in complex with zVDR LBD. (A) Binding pose and interactions of 1,20*S*(OH)_2_D_3_ in in complex with zVDR LBD. (**B**) Binding pose and interactions of 1,25(OH)_2_D_3_ in in complex with zVDR LBD. (**C**) Details of the interactions mediated by the side chain of 1,20*S*(OH)_2_D_3_ with residues of the zVDR LBD at a 4.0 Å distance cutoff. The residues numbers correspond to hVDR. (**D**) Overlay of 1,25(OH)_2_D_3_ (carbon atoms in orange and oxygen atoms in red) with 1,20*S*(OH)_2_D_3_ (grey) within zVDR LBD complexes with the indication of the hydrogen bonds formed by the ligands. The Hydrogen bonds are shown by red or yellow dashed lines and Van Der Waals interactions are shown by grey dashed lines. Hydrogen bonds are shown by dashed lines, and hydrogen-bonding residues are labelled.

To characterize molecular interactions in order to understand the mechanisms underlying the differential VDRE stimulatory effects, the *Danio rerio* VDR (zVDR) ligand binding domain (LBD) was crystallized in the presence of 1,20*S*(OH)_2_D_3_ or 1,25(OH)_2_D_3_. The overall structure of VDR-1,20*S*(OH)_2_D_3_ (PDB code: 5MX7) is highly homologous to the VDR-1,25(OH)_2_D_3_ structure, adopting the canonical active conformation. When compared to the zVDR LBD-1,25(OH)_2_D_3_ structure^19, 20^, the Cα atoms of the zVDR LBD–1,20*S*(OH)_2_D_3_ complex have a root mean square deviation of 0.25 Å over 238 residues. The ligand binds similarly to 1,25(OH)_2_D_3_ with the notable difference being that the 20*S*-OH forms a weak H-bond with His305 (3.42 Å) and does not interact with His397 (note that the residues numbers correspond to hVDR). The H-bond with His305 causes a ligand-induced conformational change in the receptor where His305 (loop6-7) is shifted by 0.63 Å to enable this H-bonding interaction. (Fig. 5). The 1α-OH and 3β-OH form similar hydrogen bonds to the zVDR to those seen with 1,25(OH)_2_D_3_.

An additional difference in the structures is that the 20*S*-OH forms a Van der Waals interaction with Val300. While most of the Van der Waals interactions are maintained, the side chain and terminal methyl groups that are differently positioned to interact differently with some of the residues (Fig. 5). Weaker interactions are formed with Leu227 (4.1 Å instead of 3.8 Å with C26) and Tyr399 (4.1 Å instead of 3.8 Å with C27), interactions compensated by stronger interactions with Val234 (3.9 Å instead of 4.2 Å with C22), and Leu412 (3.9 Å instead of 4.2 Å with C27). Overall, the hydrogen bonding interaction of 20*S*-OH with His305 and hydrophobic contacts formed by the ligand explains its agonist activity, however, with less potency than that of 1,25(OH)_2_D_3_.

### VDR translocation activity

**Figure 6:**
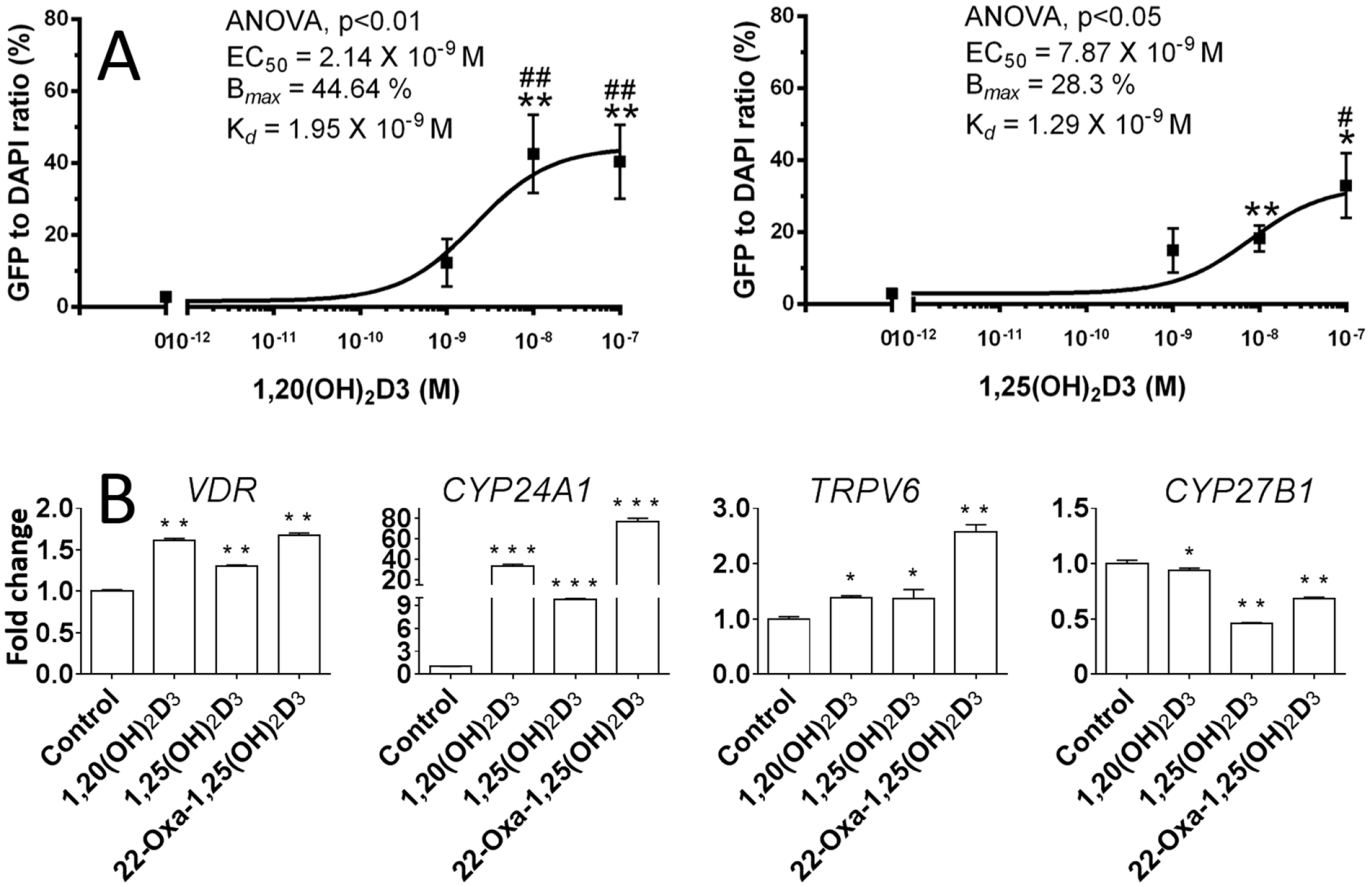
VDR translocation and gene regulation activities of 1,20*S*(OH)_2_D_3_. (**A**) The effect on vitamin D receptor (VDR) translocation from the cytoplasm to the nucleus. Data are mean ± SEM (*n *≥ 3). The dose-dependent stimulation of VDR translocation was analysed by one-way ANOVA with ^#^ *p* < 0.05 and ^##^ *p* < 0.01. The differences between control and treatment were analysed with Student’s *t*-test, where *p < 0.05 and **p < 0.01. (**B**) 1,20*S*(OH)_2_D_3_ regulates mRNA expression of genes *VDR*, *CYP24A1*, *TRPV6* and *CYP27B1* in HaCaT cells at 100 nM after 24 h treatment (*n* = 3). *p < 0.05, **p < 0.01 and ***p < 0.001.

1,25(OH)_2_D_3_ binds to cytosolic or membrane-assocoated VDR^3^, then translocation of 1,25(OH)_2_D_3_-bound VDR from the cytoplasm to the nucleus is a key step to exert its gene-regulatory effects^1, 3^. In SKMEL-188 melanoma cells transduced with pLenti-CMVVDR-EGFP-pgk-puro^21^, both 1,20*S*(OH)_2_D_3_ and 1,25(OH)_2_D_3_ showed stimulatory effects on this translocation with EC_50_ values of 2.14 × 10^−9^ and 7.87 × 10^−9^ M (Fig. 6A), respectively. The results indicate that 1,20*S*(OH)_2_D_3_ induces VDR translocation in a similar fashion to 1,25(OH)_2_D_3_.

### Regulatory activity of 1,20*S*(OH)_2_D_3_ on VDR downstream genes

To investigate how 1,20*S*(OH)_2_D_3_ affects VDR target genes through VDR activation, expression of *VDR*, *CYP24A1*, *TRPV6* and *CYP27B1* genes at the mRNA level was determined in HaCaT cells (Fig. 6B). 1,20*S*(OH)_2_D_3_ was capable of mildly upregulating the expression (1.6-fold) of the gene encoding its own receptor, the VDR, while being moderately stronger than 1,25(OH)_2_D_3_ (1.3-fold) and comparable to 22-Oxa (1.7-fold). 1,25(OH)_2_D_3_ is known to induce expression of the vitamin D catabolic enzyme, CYP24A1^14, 22^. Similarly, 1,20*S*(OH)_2_D_3_ strongly stimulates *CYP24A1* mRNA levels 34-fold, as compared to 10-fold for 1,25(OH)_2_D_3_ and 78-fold for 22-Oxa. In addition, *TRPV6* encoding an intestinal calcium channel is also a well-known target of VDR for mineral homeostasis^14, 23^. The mRNA levels of *TRPV6* were increased by 1.4-, 1.4- and 2.6-fold for 1,20*S*(OH)_2_D_3_, 1,25(OH)_2_D_3_ and 22-Oxa, respectively. Moreover, VDR activation induced by its agonists inhibits the expression of the vitamin D activation enzyme, CYP27B1^24, 25^. Although less than 1,25(OH)_2_D_3_ and 22-Oxa, 1,20*S*(OH)_2_D_3_ slightly but significantly inhibited the expression of *CYP27B1*. These results indicate that 1,20*S*(OH)_2_D_3_ is able to activate the VDR, and exert its effects through regulating VDR target genes in a similar manner to 1,25(OH)_2_D_3_. Since 1,20*S*(OH)_2_D_3_ affected the expression of *VDR*, *TRPV6* and *CYP27B1* weakly (~2-fold), further investigation on their protein levels will be beneficial to confirm the actions of 1,20*S*(OH)_2_D_3_.

### Anti-inflammatory activity

The anti-inflammatory effect of 1,20*S*(OH)_2_D_3_ was determined in mouse splenocytes stimulated by lipopolysaccharide prior to treatments with the secosteroids. The concentrations of IFNγ and IL1β in the culture media were significantly reduced by 1,20*S*(OH)_2_D_3_, compared with the control (Table 1). The effect of 1,20*S*(OH)_2_D_3_ (1.0 nM) was comparable with or slightly weaker than that of 1,25(OH)_2_D_3_ but weaker than 22-Oxa for reduction of IFNγ production. In contrast, 1,20*S*(OH)_2_D_3_ (100 nM) showed equal efficacy to 22-Oxa, and higher than that of 1,25(OH)_2_D_3_ for reduction of IL1β production. These studies suggested that 1,20*S*(OH)_2_D_3_, acting similarly to 1,25(OH)_2_D_3_ and 22-Oxa, is a potent anti-inflammatory agent.

## Conclusions

Similar to 1,25(OH)_2_D_3_, 1,20*S*(OH)_2_D_3_ can interact with the VDR with high potency, as evidenced by its ability to stimulate its translocation to the nucleus, regulate VDR downstream genes (including but not limited to *VDR*, *CYP24A1*, *TRPV6* and *CYP27B1*), and exert strong anti-inflammatory activity. The crystal structure of 1,20*S*(OH)_2_D_3_ bound to the VDR reveals differences from the 1,25(OH)_2_D_3-_bound form with respect to their interactions, including the important role of the H-bond between the 20*S*-OH and His305 that shifts the position of this residue compared to the 1,25(OH)_2_D_3_-bound form. This difference may contribute to their differential activities of these secosteroids such as the lower calcemic activity of 1,20*S*(OH)_2_D_3_ compared to 1,25(OH)_2_D_3_^8^. This study provides a molecular basis for the rational design and practical synthesis of novel 1,20*S*(OH)_2_D_3_ analogs that interact with VDR for future drug development. 1,20*S*(OH)_2_D_3_ was successfully chemically synthesized for the first time, providing ample material for further characterization of its biological activities, including animal studies in the future. The 1α,3β-diacetoxypregn-5-en-20-one (**3**) intermediate can serve as a common precursor for production of other 1,20*S*(OH)_2_D_3_ analogs which will facilitate the synthesis of similar secosteroids containing a 1α-OH group.

## Methods

### General procedures

Reagents and solvents for the synthesis were anhydrous (purchased or self-dried) to ensure good product yield. Solvents used for separations were ACS chemical grade, purchased from commercial sources and used upon arrival. NH_4_Cl was sublimed in our lab for Birch reduction. Reactions for light sensitive compounds (7DHC or D_3_ structures) were protected from light by wrapping flasks with aluminum foil, and were monitored under UV lights. Moisture-sensitive reactions were carried out under argon gas in flame-dried flasks. Reactions for non-UV active compounds were visualized on TLC by 5% phosphomolybdic acid in ethanol. All NMR data were collected on a Bruker Avance III 400 MHz NMR or a Varian Inova 500 MHz NMR. Samples were dissolved in 0.5 mL CDCl_3_, methanol-*d*_4_, DMSO-*d*_6_ or actone-*d*_6_, and NMR data were collected at r.t. Mass spectra of compounds were acquired using a Bruker LC-IT-MS system with an ESI source. High-resolution MS spectra and extracted ion chromatogram (EIC) were obtained using a Waters ACQUITY UPLC I-Class System equipped with a Xevo G2-S QTof mass spectrometer based on our previously reported conditions^26, 27^. Reaction mixtures were extracted with ethyl acetate, DCM or hexanes, washed with aqueous Na_2_CO_3_, brine, and water, and then dried over anhydrous Na_2_SO_4_. The solution was transferred to a round-bottom flask and dried by rotary evaporator. The purities of final products were determined by HPLC as >98% (Fig. 4).

### Crystallization of intermediate 15

To a clean test tube (13 × 100 mm), 18 mg of compound **15** powder and 3 mL anhydrous *n*-hexane were added. The tube was shaken until the solid was completely dissolved, then sealed with 5 layers of sealing film (Para film) membrane. The resulting solution was allowed to stand in a quiet environment for 10 days, by which time the hexane had evaporated, leaving crystals of **15** which were collected for crystallographic analysis (Supplementary Information).

### Crystallization and structural analysis of 1,20*S*(OH)_2_D_3_–VDR complex

cDNA encoding zVDR LBD (156–453 AA) was subcloned into pET28b vector to generate an *N*-terminal His-tag fusion protein. Purification was carried out as previously described, including metal affinity chromatography and gel filtration^28^. The protein was concentrated using Amicon ultra-30 (Millipore) to 3–7 mg/mL and incubated with a two-fold excess of ligand and a three-fold excess of the coactivator SRC-1 peptide (686-RHKILHRLLQEGSPS-698). Crystals were obtained in 50 mM Bis–Tris pH 6.5, 1.6 M lithium sulfate and 50 mM magnesium sulfate. Protein crystals were mounted in a fiber loop and flash-cooled under a nitrogen flux after cryo-protection with 20% glycerol. Data collection from a single frozen crystal was performed at 100 K on the ID23-1 beamline at ESRF (France). The raw data were processed and scaled with the HKL2000 program suite^29^. The crystals belong to the space group P6_5_22, with one LBD complex per asymmetric unit. The structure was solved and refined using BUSTER^30^, Phenix^31^ and iterative model building using COOT^32^. Crystallographic refinement statistics are presented in Supplementary Table S9. All structural figures were prepared using PyMOL (www.pymol.org/).

### Biosynthesis of 1,20*S*(OH)_2_D_3_

Enzymatic synthesis of 1,20*S*(OH)_2_D_3_ involved the 20*S*-hydroxylation of 1α(OH)D_3_ by recombinant bovine CYP11A1 and was carried out as described in detail before^7^. HPLC comparison was determined by using an Agilent HPLC 1100 system and a Phenomenex Luna-PFP C_18_ column (5 µm, 250 mm × 4.6 mm, Torrance, CA) at 25 °C and a flow rate of 1.0 mL/min. MeCN: H_2_O and MeOH: H_2_O were used as mobile phases with a gradient comprising 50–100% organic solvent for 30 min. 263 nm was used to display chromatograms.

### VDRE-reporter assay

HaCat, Caco-2 and Jurkat cells were transduced by lentiviral VDRE-reporter (luciferase) vector^12–15^. Caco-2 cells were grown in Dulbecco’s Modified Eagle Medium (DMEM) containing 10% fetal bovine serum (FBS) and 1% penicillin/streptomycin/amphotericin antibiotic solution (Ab) (Sigma-Aldrich, St. Louis, MO). HaCaT cells were grown in DMEM supplemented with 5% FBS and 1% Ab. Jurkat cells were grown in RPMI 1640 medium containing 10% FBS and 1% Ab. All cells were cultured at 37 °C in a humidified atmosphere containing 5% CO_2_. All cell lines were selected for at least one week by medium containing additional 1.0 µg/mL puromycin before treatment with secosteroid. Each cell line was then plated in a 96-well plate (10,000 cells/100 µL medium/well) using FBS-free media and incubated for 24 h. 1,20*S*(OH)_2_D_3_, 1,25(OH)_2_D_3_ and 22-Oxa at a series of concentrations in 10% DMSO were added separately to 96-well plates (1.0 µL/well), while 10% DMSO was used as control. After 24 h incubation, 100 µL of ONE-Glo^TM^ Luciferase Assay System (Promega, Madison, WI) was added to each well. After 5 min at r.t., the signal was recorded by a BioTek Synergy HT microplate reader (BioTek Instruments, Inc., Winooski, VT, US). All concentrations of secosteroids were tested in triplicate.

### VDR translocation assay

The effects of 1,20 *S*(OH)_2_D_3_ on VDR translocation from the cytoplasm to the nucleus were tested on the previously described SKMEL-188 cell model^21, 33^, using cells stably transduced with pLenti-CMV-VDREGFP-pgk-puro (VDR and EGFP expressed as fusion protein)^34^. Cells were treated with secosteroids (up to 100 nM) for 90 min followed by analysis with Cytation 5 (BioTek, Winooski, VT, US). Translocation to the nucleus was determined by counting cells with a fluorescent nucleus and the results are presented as the percentage of the total cells that displayed nuclear staining, as described previously^21^. The data were obtained from at least two separate experiments, with images taken in the central area from at least three different wells and counted as described^21, 34^.

### Real-time PCR assay

HaCaT cells were seeded in 60 mm dishes (1 million/dish) in 10 mL DMEM supplemented with 5% FBS and 1% Ab. After overnight incubation they were cultured in FBS-free medium for another 12 h to synchronize the cells. The media were then removed and secosteroids in DMEM (5% FBS and 1% Ab) with a concentration of 100 nM were added to the dishes. After 24 h incubation, media were removed, and 10 mL PBS was used to wash the dish. Cells were then detached by trypsin, centrifuged in Eppendorf tubes, washed with PBS (5 mL), and stored at −80 °C. Absolutely RNA Miniprep Kit (Stratagene, La Jolla, CA, USA) was used to isolate the RNA, and Transcriptor First Strand cDNA Synthesis Kit (Roche Inc., Mannheim, Germany) was used for reverse transcription (100 ng RNA/reaction). Real-time PCR was carried out using cDNA which was diluted 10-fold in sterile water and a SYBR Green PCR Master Mix. The forward reverse primers for VDR, CYP24A1, TRPV6 and CYP27B1 genes were designed based on the rat and mouse sequences using Primer Quest software (Integrated Device Technology, San Jose, CA, USA). Reactions (*n = *3) were performed at 50 °C for 2 min, 95 °C for 10 min and then 40 cycles of 95 °C for 15 s, 60 °C for 30 s and 72 °C for 30 s. Data were collected and analyzed on a Roche Light Cycler 480. Using a comparative Ct method^25^, the amount of the final amplified product was normalized to the amount of β-actin as a housekeeping gene.

### IFNγ production assay

All animal experiments in this study were performed in accordance with the NIH animal use guidelines and protocol (protocol No.: 15–043.0) approved by the Institutional Animal Care and Use Committee (IACUC) at the University of Tennessee Health Science Center (UTHSC, Memphis, TN). Splenocytes were isolated from 7-week old C57BL/6 female mice and cultured at 2 × 10^6^/mL 500 μL/well for 72 h at 37 °C in a humidified atmosphere. Harvested supernatants were analyze for levels of murine IFNγ by ELISA (R & D Systems Minneapolis, MN) according to the manufacturer’s instructions. Results are expressed as mean [IFNγ] ± SEM of triplicate determinations (pg/mL) of culture supernatant. The difference in [IFNγ] between EtOH vehicle + anti-CD3e MOAB (0.08 μg/mL) and [IFNγ] in each of the cultures containing D_3_ analog was analyzed by one way ANOVA Sigma. The amount of EtOH in the EtOH + PBS and EtOH + anti-CD3e MOAB culture was equal to that in the cultures of the D_3_ analogs at a level of 10^−9^ M. Culture medium was RPMI 1640 supplemented with 9% charcoal stripped fetal bovine serum, non-essential amino acids, HEPES buffer Glutamax, penicillin 100 μg/mL, streptomycin 100 μg/mL, fungizone 1 μg/mL (GIBCO, Grand Island, NY) and 50 μM β-mercaptoethanol (Sigma, St. Louis, MO)^35^.

### IL1β production assay

Splenocytes were isolated from 7-week old C57BL/6 female mice and cultured at 2 × 10^6^/mL, 500 μL/well, for 24 h at 37 °C in a humidified atmosphere. The vitamin D analogs or EtOH vehicle were added to the splenocyte cultures 2 h prior to addition of Lipopolysaccharide W *E*. *coli* 055:B5 (LPS) (Difco Lab. Defrost MI) 100 ng/mL or PBS vehicle. Harvested supernatants were analyzed for levels of murine IL1β by ELISA (R & D Systems Minneapolis, MN) according to the manufacturer’s instructions. Results are expressed as mean IL1β concentration ± SEM of triplicate determinations (pg/mL) of culture supernatant. The amount of EtOH in the EtOH + PBS and EtOH + LPS culture was equal to that in the cultures of the vitamin D analogs at a level of 10^-7^ M. Culture medium was RPMI 1640 supplemented with 9% charcoal stripped fetal bovine serum, non-essential amino acids, HEPES buffer Glutamax, penicillin 100 μg/mL, streptomycin 100 μg/mL, fungizone 1 μg/mL (GIBCO, Grand Island, NY) and 50 μM β-mercaptoethanol (Sigma, St. Louis, MO). The difference in [IL1β] between control and D_3_ analog treatment was analyzed by one way ANOVA (Sigma Plot 13.0).

## Electronic supplementary material

Supplementary Info

## References

[1] Lin Z, Li W (2016). The roles of vitamin D and its analogs in inflammatory diseases. Curr. Top. Med. Chem..

[2] Wierzbicka J, Piotrowska A, Zmijewski MA (2014). The renaissance of vitamin D. Acta Biochim. Pol..

[3] Haussler MR (2008). Vitamin D receptor: molecular signaling and actions of nutritional ligands in disease prevention. Nutr. Rev..

[4] Christakos, S. *et al*. Vitamin D: Metabolism, Molecular Mechanism of Action, and Pleiotropic Effects. *Physiol*. *Rev*. **96**, 365–408, doi:10.1152/physrev.00014.2015 (2016). 10.1152/physrev.00014.2015 PMC4839493 26681795

[5] Slominski AT (2012). *In vivo* evidence for a novel pathway of vitamin D(3) metabolism initiated by P450scc and modified by CYP27B1. FASEB J..

[6] Li W (2010). Chemical synthesis of 20S-hydroxyvitamin D3, which shows antiproliferative activity. Steroids.

[7] Tuckey RC (2008). Metabolism of 1alpha-hydroxyvitamin D3 by cytochrome P450scc to biologically active 1alpha,20-dihydroxyvitamin D3. J. Steroid Biochem. Mol. Biol..

[8] Slominski AT (2010). Products of vitamin D3 or 7-dehydrocholesterol metabolism by cytochrome P450scc show anti-leukemia effects, having low or absent calcemic activity. PLoS One.

[9] Slominski AT (2014). The role of CYP11A1 in the production of vitamin D metabolites and their role in the regulation of epidermal functions. J. Steroid Biochem. Mol. Biol..

[10] Slominski AT (2012). Novel vitamin D hydroxyderivatives inhibit melanoma growth and show differential effects on normal melanocytes. Anticancer Res..

[11] Slominski AT (2015). Detection of novel CYP11A1-derived secosteroids in the human epidermis and serum and pig adrenal gland. Sci. Rep..

[12] Lin Z (2015). Chemical synthesis and biological activities of 20S,24S/R-dihydroxyvitamin D3 epimers and their 1alpha-hydroxyl derivatives. J. Med. Chem..

[13] Lin Z (2016). Synthesis and Biological Evaluation of Vitamin D3 Metabolite 20S,23S-Dihydroxyvitamin D3 and Its 23R Epimer. J. Med. Chem..

[14] Lin Z (2016). Design, Synthesis and Biological Activities of Novel Gemini 20S-Hydroxyvitamin D3 Analogs. Anticancer Res..

[15] Wang Q (2015). Total synthesis of biologically active 20S-hydroxyvitamin D3. Steroids.

[16] Yin YZ, Liu C, Tang LQ, Liu ZP (2012). Recoverable Pd/C catalyst mediated dehydrogenation of sterols and an improved synthesis of 1alpha-hydroxydehydroepiandrosterone. Steroids.

[17] Poza J (2007). Synthesis and evaluation of new 6-hydroximinosteroid analogs as cytotoxic agents. Bioorg. Med. Chem..

[18] Mouriño A (1978). An improved synthesis of 1α, 3β-dihydroxycholesta-5, 7-diene. Synth. Commun..

[19] Otero R (2016). Carborane-based design of a potent vitamin D receptor agonist. Chem. Sci..

[20] Huet T (2015). A vitamin D receptor selectively activated by gemini analogs reveals ligand dependent and independent effects. Cell Rep..

[21] Slominski AT (2011). 20-Hydroxyvitamin D2 is a noncalcemic analog of vitamin D with potent antiproliferative and prodifferentiation activities in normal and malignant cells. Am. J. Physiol. Cell Physiol..

[22] St-Arnaud R (2010). CYP24A1-deficient mice as a tool to uncover a biological activity for vitamin D metabolites hydroxylated at position 24. J. Steroid Biochem. Mol. Biol..

[23] Clapham DE, Julius D, Montell C, Schultz G (2005). International Union of Pharmacology. XLIX. Nomenclature and structure-function relationships of transient receptor potential channels. Pharmacol. Rev..

[24] Holick MF (2006). Resurrection of vitamin D deficiency and rickets. J. Clin. Invest..

[25] Zbytek B (2008). 20-Hydroxyvitamin D3, a product of vitamin D3 hydroxylation by cytochrome P450scc, stimulates keratinocyte differentiation. J. Invest. Dermatol..

[26] Pingili AK (2015). 6beta-hydroxytestosterone, a cytochrome P450 1B1 metabolite of testosterone, contributes to angiotensin II-induced hypertension and its pathogenesis in male mice. Hypertension.

[27] Lin Z, Yang R, Guan Z, Chen A, Li W (2014). Ultra-performance LC separation and quadrupole time-of-flight MS identification of major alkaloids in Plumula Nelumbinis. Phytochem. Anal..

[28] Huet T (2011). Structure–function study of gemini derivatives with two different side chains at C-20, Gemini-0072 and Gemini-0097. MedChemComm.

[29] Otwinowski Z, Minor W (1997). Processing of X-ray diffraction data collected in oscillation mode. Methods Enzymol..

[30] Bricogne, G. *et al*. BUSTER version 2.11. 2. Cambridge, United Kingdom: Global Phasing Ltd (2011).

[31] Adams PD (2010). PHENIX: a comprehensive Python-based system for macromolecular structure solution. Acta Crystallogr. D Biol. Crystallogr..

[32] Emsley P, Cowtan K (2004). Coot: model-building tools for molecular graphics. Acta Crystallogr. D Biol. Crystallogr..

[33] Slominski A, Zbytek B, Slominski R (2009). Inhibitors of melanogenesis increase toxicity of cyclophosphamide and lymphocytes against melanoma cells. Int. J. Cancer.

[34] Kim T-K (2012). Correlation between secosteroid-induced vitamin D receptor activity in melanoma cells and computer-modeled receptor binding strength. Mol. Cell. Endocrinol..

[35] Slominski AT (2014). RORalpha and ROR gamma are expressed in human skin and serve as receptors for endogenously produced noncalcemic 20-hydroxy- and 20,23-dihydroxyvitamin D. FASEB J..

