# Supplementary material for: 1α,20S-Dihydroxyvitamin D3 Interacts with Vitamin D Receptor: Crystal Structure and Route of Chemical Synthesis
Source: Sci Rep. 2017 Aug 31;7:10193. doi: 10.1038/s41598-017-10917-7 (PMC5579064; doi:10.1038/s41598-017-10917-7)

Supplementary Information

1*α*,20*S*-Dihydroxyvitamin D_3 Interacts with Vitamin D Receptor: Crystal Structure and Route of Chemical Synthesis

Zongtao Lin,[1] Hao Chen,[1] Anna Y. Belorusova,[2,9] John C. Bollinger,[3] Edith K.Y. Tang,[4] Zorica Janjetovic,[5] Tae-Kang Kim,[5] Zhongzhi Wu,[1] Duane D. Miller,[1] Andrzej T. Slominski,[5,6] Arnold E. Postlethwaite,[7,8] Robert C. Tuckey,[4] Natacha Rochel,[2] and Wei Li[1,]*

[1]Department of Pharmaceutical Sciences, University of Tennessee Health Science Center, 881 Madison Avenue, Room 561, Memphis, TN 38163, United States.
[2]Department of Integrative Structure Biology, IGBMC - CNRS UMR7104 – Inserm U964, 1, rue Laurent Fries, Illkirch 67400, France.
[3]Department of Structural Biology, St. Jude Children's Research Hospital, Memphis, TN 38105, United States.
[4]School of Chemistry and Biochemistry, University of Western Australia, Crawley, WA 6009, Australia.
[5]Department of Dermatology, University of Alabama at Birmingham, Birmingham, AL 35294, United States.
[6]VA Medical Center, Birmingham, AL 35294, United States.
[7]Department of Medicine, University of Tennessee Health Science Center, Memphis, TN 38163, United States.
[8]VA Medical Center, Memphis, TN 38104, United States.
[9]Present Address. Department of Medicinal Chemistry, RIA iMed, AstraZeneca R&D, Pepparedsleden 1, S-431 83 Mölndal, Sweden.

Table of Contents

1. Chemistry. 2
1.1. Synthesis of 5....................2
1.2. Synthesis of 6....................2
1.3. Synthesis of 7....................2
1.4. Synthesis of 8....................2
1.5. Synthesis of 9....................3
1.6. Synthesis of 10....................3
1.7. Synthesis of 11....................4
1.8. Synthesis of 12....................4
1.9. Synthesis of 13....................4
1.10. Synthesis of 14a. 5
1.11. Synthesis of 14....................5
1.12. Synthesis of 15....................5
1.13. Synthesis of 16....................6
1.14. Synthesis of 3....................6
1.15. Synthesis of 17....................7
1.16. Synthesis of 18....................7
1.17. Synthesis of 2....................7
1.18. Synthesis of 1....................8
2. Identification of 1α-OH in 15 by NOESY. 8
3. Crystal structure analysis of 15....................8
4. Crystallographic analysis of VDR-1,20S$(OH)_2D_3$. 18
5. Spectra. 19
6. Checkcif of 15. 87

1. Chemistry.

1.1. Synthesis of 5.

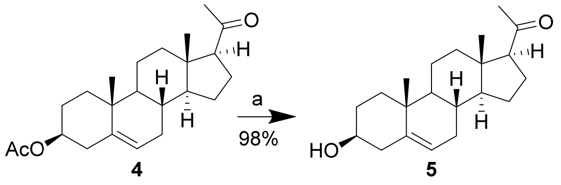

1-((3S,8S,10R,13S,14S,17S)-3-hydroxy-10,13-dimethyl-2,3,4,7,8,9,10,11,12,13,14,15,16,17-tetradecahydro-1H-cyclopenta[a]phenanthren-17-yl)ethan-1-one. To a stirred mixture of pregnenolone acetate (**4**) (10.3 g, 28.8 mmol) in methanol (150 mL) was added K_2CO_3 (19.8 g, 143.8 mmol, 5 equiv.). The reaction mixture was stirred overnight at room temperature. Ice water (1.9 L) was added, and the reaction mixture was stirred for 30 min, filtered, and washed by distilled water (500 mL) to give **5** as a white solid (8.9 g, 28.2 mmol, 98%). ^{1}H NMR (400 MHz, Chloroform-*d*) δ 5.35 (dt, *J* = 5.4, 2.0 Hz, 1H), 3.52 (td, *J* = 11.0, 5.5 Hz, 1H), 2.54 (t, *J* = 9.0 Hz, 1H), 2.38 – 2.15 (m, 4H), 2.13 (s, 3H), 2.09 – 1.95 (m, 2H), 1.94 – 1.78 (m, 2H), 1.77 – 1.38 (m, 8H), 1.36 – 1.04 (m, 3H), 1.01 (s, 3H), 0.63 (s, 3H). MS (ESI) m/z 339.3 $[M + Na]^+$.

1.2. Synthesis of 6.

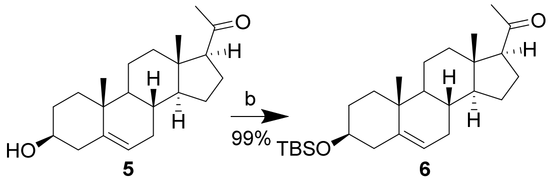

1-((3S,8S,10R,13S,14S,17S)-3-((tert-butyldimethylsilyl)oxy)-10,13-dimethyl-2,3,4,7,8,9,10,11,12,13,14,15,16,17-tetradecahydro-1H-cyclopenta[a]phenanthren-17-yl)ethan-1-one. To a stirred mixture of **5** (8.9 g, 28.2 mmol) in DMF (freshly distilled over CaH_2, 200 mL) was added *tert*-butyldimethylsilyl chloride (8.5 g, 56.3 mmol, 2 equiv.) and imidazole (7.7 g, 112.6 mmol, 4 equiv.). The above mixture was stirred at r.t. overnight until completion as monitored by TLC (20% EA in hexane). The reaction was quenched by addition of ice water (1.8 L), the precipitate was filtered, washed with water (400 mL), and dried under vacuum to give **6** (11.9 g, 27.9 mmol, 99%) as a white solid. ^{1}H NMR (400 MHz, Chloroform-*d*) δ 5.26 (dq, *J* = 5.8, 1.7 Hz, 1H), 3.42 (tt, *J* = 11.0, 4.8 Hz, 1H), 2.47 (t, *J* = 8.9 Hz, 1H), 2.20 (ddtd, *J* = 14.4, 8.7, 6.2, 5.8, 3.0 Hz, 1H), 2.15 – 2.08 (m, 1H), 2.06 (s, 3H), 2.03 – 1.86 (m, 2H), 1.76 (dt, *J* = 13.2, 3.5 Hz, 1H), 1.71 – 1.31 (m, 9H), 1.26 – 0.95 (m, 4H), 0.94 (s, 3H), 0.93 – 0.87 (m, 1H), 0.83 (s, 9H), 0.57 (s, 3H), 0.00 (s, 6H). MS (ESI) m/z 453.4 $[M + Na]^+$.

1.3. Synthesis of 7.

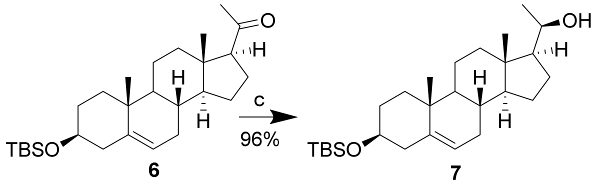

(1R)-1-((3S,8S,10R,13S,14S,17S)-3-((tert-butyldimethylsilyl)oxy)-10,13-dimethyl-2,3,4,7,8,9,10,11,12,13,14,15,16,17-tetradecahydro-1H-cyclopenta[a]phenanthren-17-yl)ethan-1-ol. To a stirred solution of **6** (11.9 g, 27.9 mmol) in 100 mL DCM:MeOH (1:1), $NaBH_4$ (1060 mg, 27.9 mmol, 1.0 equiv.) was added at 0 ºC. The reaction was allowed to reach r.t. and stirred until completion monitored by TLC (20% EA in hexane). The mixture was added sat. Na_2CO_3 (300 mL) and stirred for 30 min, extracted with DCM (3 × 100 mL). The organic layer was combined, washed with brine (100 mL) and water (2 × 100 mL), dried over anhydrous Na_2SO_4. The crude mixture was subjected to flash chromatography (10% ethyl acetate in hexanes) to give **7** (11.6 g, 26.8 mmol, 96%) as a white solid. ^{1}H NMR (400 MHz, Chloroform-*d*) δ 5.26 (dt, *J* = 5.5, 1.9 Hz, 1H), 3.68 (dt, *J* = 9.7, 5.8 Hz, 1H), 3.42 (tt, *J* = 11.0, 4.7 Hz, 1H), 2.29 – 2.15 (m, 1H), 2.11 (ddd, *J* = 13.1, 5.1, 2.3 Hz, 1H), 2.01 (dt, *J* = 12.5, 3.5 Hz, 1H), 1.97 – 1.85 (m, 1H), 1.76 (dt, *J* = 13.3, 3.6 Hz, 1H), 1.70 – 1.33 (m, 9H), 1.33 – 1.15 (m, 2H), 1.12 (d, *J* = 5.5 Hz, 1H), 1.08 (d, *J* = 6.1 Hz, 3H), 1.04 – 0.97 (m, 2H), 0.95 (s, 3H), 0.89 (td, *J* = 11.1, 5.5 Hz, 1H), 0.83 (s, 9H), 0.71 (s, 3H), 0.00 (s, 6H). MS (ESI) m/z 455.4 $[M + Na]^+$.

1.4. Synthesis of 8.

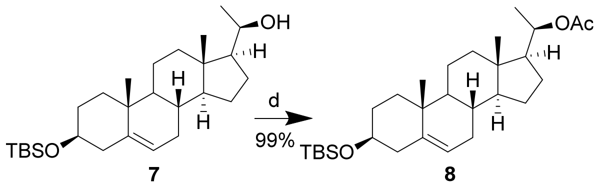

(1R)-1-((3S,8S,10R,13S,14S,17S)-3-((tert-butyldimethylsilyl)oxy)-10,13-dimethyl-2,3,4,7,8,9,10,11,12,13,14,15,16,17-tetradecahydro-1H-cyclopenta[a]phenanthren-17-yl)ethyl acetate. To a solution of alcohol **7** (10.1 g, 23.4 mmol) in DCM (100 mL) was added acetic anhydride (11.9 g, 117.0 mmol, 5 equiv.), Et_3N (24.3 g, 234 mmol, 10 equiv.) and catalytic DMAP (0.05 equiv.). The reaction mixture was stirred at r.t. overnight. The solvents were removed under reduced pressure, and the resulting mixture was subjected to flash chromatography (10% EtOAc in hexane) to give **8** (11.0 g, 23.2 mmol, 99%) as a white solid. ^{1}H NMR (400 MHz, Chloroform-*d*) δ 5.32 (dt, *J* = 4.9, 1.8 Hz, 1H), 4.85 (dq, *J* = 10.4, 6.1 Hz, 1H), 3.48 (tt, *J* = 11.0, 4.7 Hz, 1H), 2.27 (ddd, *J* = 13.7, 10.9, 2.8 Hz, 1H), 2.17 (ddd, *J* = 13.4, 5.0, 2.2 Hz, 1H), 2.02 (d, *J* = 7.3 Hz, 3H), 1.96 (q, *J* = 3.7, 2.4 Hz, 1H), 1.92 – 1.34 (m, 12H), 1.25 (ddq, *J* = 14.7, 12.9, 8.7, 7.3 Hz, 2H), 1.16 (d, *J* = 6.1 Hz, 3H), 1.13 – 1.01 (m, 2H), 1.01 (s, 3H), 0.95 (dt, *J* = 11.2, 5.8 Hz, 1H), 0.90 (s, 9H), 0.65 (s, 3H), 0.06 (s, 6H). ^{13}C NMR (101 MHz, $CDCl_3$) δ 170.44, 141.63, 120.95, 77.21, 72.88, 72.61, 56.17, 55.00, 50.26, 42.82, 42.18, 39.22, 37.40, 36.61, 32.10, 31.93, 31.77, 25.94, 24.32, 21.54, 20.97, 19.95, 19.44, 18.27, 12.37, -4.57. MS (ESI) m/z 497.4 $[M + Na]^+$.

1.5. Synthesis of 9.

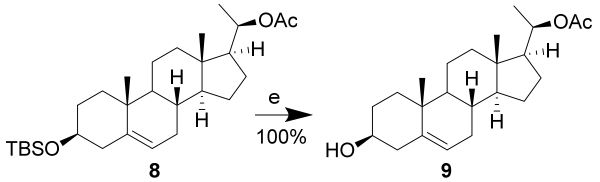

(1R)-1-((3S,8S,10R,13S,14S,17S)-3-hydroxy-10,13-dimethyl-2,3,4,7,8,9,10,11,12,13,14,15,16,17-tetradecahydro-1H-cyclopenta[a]phenanthren-17-yl)ethyl acetate. To a solution of ester **8** (10.4 g, 22.0 mmol) in THF (100 mL) was added tetrabutylammonium fluoride (1.0 M in THF, 43.9 mL, 2.0 equiv.), and the mixture was stirred at room temperature for 12 h. The reaction mixture was quenched with sat. $NaHCO_3$ (400 mL), then extracted with ethyl acetate (3 × 200 mL). The combined organic layer was washed with brine (200 mL) and H_2O (200 mL), and dried over Na_2SO_4. The crude mixture resulting from removing solvents under reduced pressure was subjected to flash chromatography (30% ethyl acetate in hexane) to give an alcohol with quantitative yield. ^{1}H NMR (400 MHz, Chloroform-*d*) δ 5.35 (dt, *J* = 5.3, 1.9 Hz, 1H), 4.84 (dq, *J* = 10.3, 6.1 Hz, 1H), 3.52 (q, *J* = 8.8, 6.1 Hz, 1H), 2.38 – 2.13 (m, 2H), 2.02 (s, 3H), 1.96 (td, *J* = 4.9, 2.4 Hz, 1H), 1.89 – 1.78 (m, 3H), 1.78 – 1.33 (m, 9H), 1.33 – 1.18 (m, 3H), 1.16 (d, *J* = 6.1 Hz, 3H), 1.14 – 1.02 (m, 2H), 1.01 (d, *J* = 2.0 Hz, 3H), 0.96 (ddd, *J* = 12.0, 10.7, 5.0 Hz, 1H), 0.65 (s, 3H). ^{13}C NMR (101 MHz, $CDCl_3$) δ 170.46, 140.83, 121.48, 72.88, 71.75, 56.12, 54.98, 50.16, 42.29, 42.17, 39.18, 37.26, 36.52, 31.89, 31.76, 31.66, 25.46, 24.32, 21.54, 20.98, 19.94, 19.40, 12.38. MS (ESI) m/z 383.4 $[M + Na]^+$.

1.6. Synthesis of 10.

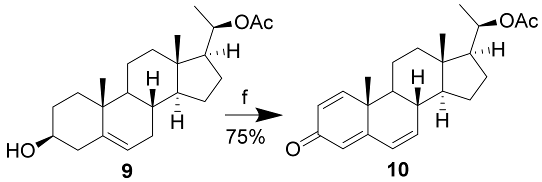

(1R)-1-((8S,10R,13S,14S,17S)-10,13-dimethyl-3-oxo-8,9,10,11,12,13,14,15,16,17-decahydro-3H-cyclopenta[a]phenanthren-17-yl)ethyl acetate. A solution of **9** (5.0 g, 13.9 mmol) and DDQ (12.6 g, 55.6 mmol) in 1,4-dioxane (250 mL) freshly dried over NaH was refluxed for 4 hours. The reaction mixture was cooled to r.t., 40% ethyl acetate in hexane (250 mL) was added, and the mixture was stirred for 3 min and filtered. The filtrate was dried and subjected to flash chromatography twice (15% ethyl acetate in hexane and 15% ethyl acetate in DCM) to afford **10** (3.7 g, 10.4 mmol, 75%) as white crystals. ^{1}H NMR (400 MHz, Chloroform-*d*) δ 6.99 (d, *J* = 10.1 Hz, 1H), 6.17 (ddd, *J* = 9.8, 6.1, 2.3 Hz, 2H), 5.95 (dd, *J* = 11.5, 2.0 Hz, 2H), 4.81 (dq, *J* = 10.5, 6.1 Hz, 1H), 2.21 (t, *J* = 10.3 Hz, 1H), 1.96 (s, 3H), 1.90 – 1.64 (m, 3H), 1.57 (ddd, *J* = 16.8, 9.4, 3.8 Hz, 2H), 1.38 (ddd, *J* = 13.0, 9.9, 3.7 Hz, 1H), 1.34 – 1.14 (m, 4H), 1.12 (s, 3H), 1.10 (d, *J* = 6.1 Hz, 3H), 0.92 – 0.75 (m, 1H), 0.69 (s, 3H). ^{13}C NMR (101 MHz,

CDCl$_3$) δ 186.35, 170.37, 162.54, 152.90, 138.23, 128.13, 127.72, 123.81, 72.59, 54.69, 53.03, 48.40, 42.81, 41.20, 38.82, 38.01, 25.36, 23.69, 21.76, 21.52, 20.73, 19.88, 12.50. MS (ESI) m/z 377.5 $[M + Na]^+$.

1.7. Synthesis of 11.

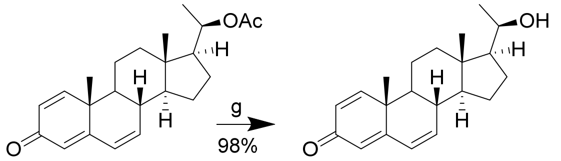

10 **11**

(8S,10R,13S,14S,17S)-17-((R)-1-hydroxyethyl)-10,13-dimethyl-8,9,10,11,12,13,14,15,16,17-decahydro-3H-cyclopenta[a]phenanthren-3-one. To a stirred mixture of **10** (3.0 g, 8.5 mmol) in methanol (100 mL) was added KOH (2.4 g, 42.4 mmol, 5 equiv.). The reaction mixture was stirred for 3 h at room temperature. Sat. $NaHCO_3$ (900 mL) was added, the mixture was extracted with DCM (3 × 200 mL). The organic layer was combined, washed with brine (200 mL) and water (200 mL), and dried under reduced pressure. The crude mixture was subjected to flash chromatography (30% ethyl acetate in hexane) to give **11** as a white solid (2.6 g, 8.3 mmol, 98%). ^{1}H NMR (400 MHz, Chloroform-*d*) δ 7.09 (d, *J* = 10.1 Hz, 1H), 6.25 (ddd, *J* = 10.0, 7.8, 2.4 Hz, 2H), 6.08 – 5.93 (m, 2H), 3.77 (dq, *J* = 9.8, 6.1 Hz, 1H), 2.43 – 2.15 (m, 2H), 2.03 – 1.56 (m, 4H), 1.53 – 1.24 (m, 6H), 1.22 (s, 3H), 1.18 (d, *J* = 6.1 Hz, 3H), 0.89 (s, 3H). ^{13}C NMR (101 MHz, CDCl$_3$) δ 186.35, 162.65, 153.01, 138.46, 128.08, 127.62, 123.75, 70.37, 58.08, 53.15, 48.46, 43.01, 41.25, 39.45, 38.04, 25.54, 23.96, 23.86, 21.74, 20.74, 12.39. MS (ESI) m/z 335.4 $[M + Na]^+$.

1.8. Synthesis of 12.

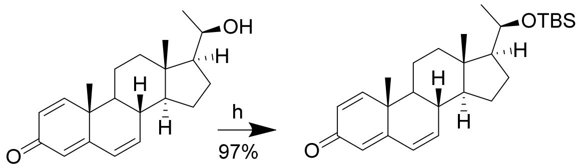

11 **12**

(8S,10R,13S,14S,17S)-17-((R)-1-((tert-butyldimethylsilyl)oxy)ethyl)-10,13-dimethyl-8,9,10,11,12,13,14,15,16,17-decahydro-3H-cyclopenta[a]phenanthren-3-one. To a stirred solution of **11** (2.4 g, 7.7 mmol) in DMF (freshly distilled over CaH_2, 50 mL) was added *tert*-butyldimethylsilyl chloride (2.3 g, 15.4 mmol, 2 equiv.) and imidazole (2.1 g, 3.1 mmol, 4 equiv.). The above mixture was stirred at r.t. overnight. The reaction was quenched by addition of ice water (150 mL), and extracted with DCM (3 × 100 mL). The organic layer was combined, washed with brine (100 mL) and water (100 mL), and dried under reduced pressure. The crude mixture was subjected to flash chromatography (15% ethyl acetate in hexane) to give **12** as a colorless sticky solid (3.3 g, 7.5 mmol, 97%). ^{1}H NMR (400 MHz, Chloroform-*d*) δ 7.10 (d, *J* = 10.1 Hz, 1H), 6.24 (ddd, *J* = 9.6, 7.4, 2.4 Hz, 2H), 6.11 – 5.99 (m, 2H), 3.91 – 3.66 (m, 1H), 2.37 – 2.16 (m, 2H), 1.90 – 1.51 (m, 3H), 1.45 (tq, *J* = 8.8, 4.4, 4.0 Hz, 2H), 1.39 – 1.23 (m, 2H), 1.21 (s, 3H), 1.11 (d, *J* = 5.9 Hz, 3H), 0.93 (d, *J* = 0.9 Hz, 3H), 0.91 (d, *J* = 0.9 Hz, 9H), 0.83 (s, 3H), 0.10 (s, 6H). ^{13}C NMR (101 MHz, CDCl$_3$) δ 186.39, 162.81, 153.14, 138.72, 128.01, 127.53, 123.68, 70.97, 58.04, 53.33, 48.58, 42.76, 41.30, 39.10, 38.11, 26.07, 25.68, 25.65, 23.81, 23.73, 21.69, 20.72, 18.08, 12.13, -3.43, -3.99. MS (ESI) m/z 449.5 $[M + Na]^+$.

1.9. Synthesis of 13.

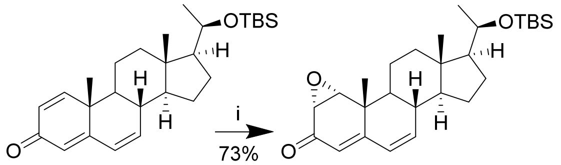

12 **13**

(2aR,3aR,3bR,5aS,6S,8aS,8bS)-6-((R)-1-((tert-butyldimethylsilyl)oxy)ethyl)-3b,5a-dimethyl-2a,3a,3b,3c,4,5,5a,6,7,8,8a,8b-dodecahydro-2H-cyclopenta[7,8]phenanthro[3,4-b]oxiren-2-one. To a solution of **12** (2.1 g, 4.9 mmol) in methanol (44.1 mL) was added 7% methanolic KOH solution (1.47 mL) and 30% H_2O_2 solution (4.9 mL) at -40 ºC. The mixture was allowed to warm up to 0 ºC and stirred at the same temperature for 12 h. The excess H_2O_2 was quenched by adding saturated Na_2SO_3 (100 mL). The mixture was extracted with EtOAc (3 × 100 mL). The combined organic layers were washed with brine (100 mL), dried over Na_2SO_4, and concentrated. The crude mixture was subjected to flash chromatography (15% ethyl acetate in hexane) to provide the epoxide **13** (1.6 g, 3.6 mmol, 73%) as colorless sticky oil. ^{1}H NMR (400 MHz, Chloroform-*d*) δ 6.26 – 5.99 (m, 2H), 5.66 (d, *J* = 1.9 Hz, 1H), 3.79 (dq, *J* = 9.1, 5.9 Hz, 1H), 3.62 (d, *J* = 4.1 Hz, 1H), 3.55 – 3.36 (m, 1H), 2.39 – 2.14 (m, 2H), 2.03 – 1.39

(m, 6H), 1.38 – 1.22 (m, 4H), 1.20 (s, 3H), 1.12 (d, *J* = 5.9 Hz, 3H), 0.91 (d, *J* = 0.9 Hz, 9H), 0.82 (s, 3H), 0.10 (s, 6H). ^{13}C NMR (101 MHz, $CDCl_3$) δ 194.79, 158.98, 140.62, 127.75, 119.39, 77.20, 70.95, 59.49, 58.04, 54.74, 53.00, 46.23, 42.74, 38.92, 37.45, 26.06, 25.64, 23.85, 23.73, 21.03, 18.49, 18.08, 12.00, -3.41, -3.99. MS (ESI) m/z 465.4 $[M + Na]^+$.

1.10. Synthesis of 14a.

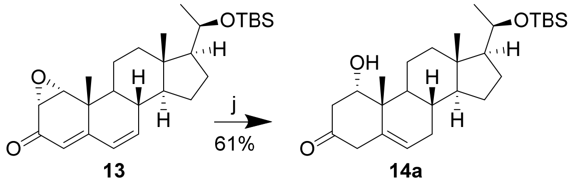

13 14a

(1S,8S,10R,13S,14S,17S)-17-((R)-1-((tert-butyldimethylsilyl)oxy)ethyl)-1-hydroxy-10,13-dimethyl-1,2,4,7,8,9,10,11,12,13,14,15,16,17-tetradecahydro-3H-cyclopenta[a]phenanthren-3-one. A four-necked flask was equipped with a powder addition funnel, a liquid dropping funnel, a cold-finger filled with dry ice-acetone and connected to an anhydrous NH_3 source. Anhydrous N_2 was swept through the system for 10 min, and then ammonia (10 mL) was trapped in the flask cooled to -80 ºC. Lithium wire (350 mg, 6.7 mmol, 50 equiv.) was added to the reaction mixture. After stirring for 30 min, the epoxide **13** (50 mg, 0.14 mmol) in THF (2 mL) was added drop-wise over 2 h. The cooling bath of the flask was changed to dry ice-MeCN (-40 ºC) to allow the reaction to warm up to -40 ºC for 1 h. The flask was dipped in a -80 ºC cooling bath, and sublimed NH_4Cl (361 mg, 6.7 mmol, 50 equiv.) was added over 5 min. The mixture turned white and pasty. Liquid NH_3 was removed via a stream of nitrogen, and the resulting residue was added to H_2O (20 mL) and extracted with EtOAc (3 × 20 mL). The organic layer was washed with brine (10 mL), dried over Na_2SO_4, and concentrated. The residue was subjected to flash chromatography (20% EtOAc in hexane) to give the **14a** (major product, 39 mg, 0.09 mmol, 65%) as a colorless solid. ^{1}H NMR (500 MHz, Chloroform-*d*) δ 5.50 (dd, *J* = 5.5, 2.8 Hz, 1H), 4.03 (s, 1H), 3.67 (dt, *J* = 10.0, 6.0 Hz, 1H), 3.30 – 3.12 (m, 1H), 2.88 (dd, *J* = 17.5, 2.4 Hz, 1H), 2.70 (dd, *J* = 15.6, 2.8 Hz, 1H), 2.45 (dt, *J* = 15.4, 3.0 Hz, 1H), 2.13 (dt, *J* = 13.1, 3.5 Hz, 1H), 2.01 – 1.88 (m, 1H), 1.62 – 1.28 (m, 10H), 1.28 – 1.13 (m, 2H), 1.11 (s, 3H), 1.01 (d, *J* = 6.0 Hz, 3H), 0.81 (d, *J* = 2.0 Hz, 9H), 0.67 (s, 3H), 0.00 (s, 6H). HRMS (ESI+) m/z 447.3289 [M + H]+ (error: -1.1 ppm).

1.11. Synthesis of 14.

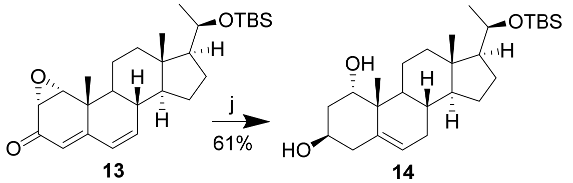

13 14

(1S,3R,8S,10R,13S,14S,17S)-17-((R)-1-((tert-butyldimethylsilyl)oxy)ethyl)-10,13-dimethyl-2,3,4,7,8,9,10,11,12,13,14,15,16,17-tetradecahydro-1H-cyclopenta[a]phenanthrene-1,3-diol. A four-necked flask was equipped with a powder addition funnel, a liquid dropping funnel, a cold-finger filled with dry ice-acetone and connected to an anhydrous NH_3 source. Anhydrous N_2 was swept through the system for 10 min, and then ammonia (150 mL) was trapped in the flask cooled to -80 ºC. Lithium wire (1.1 g, 177.6 mmol, 50 equiv.) was added to the reaction mixture. After stirring for 30 min, the epoxide **13** (1.6 g, 3.6 mmol) in THF (40 mL) was added drop-wise over 2 h. The cooling bath of the flask was changed to dry ice-MeCN (-40 ºC) to allow the reaction to warm up to -40 ºC for 1 h. The flask was dipped in a -80 ºC cooling bath, and sublimed NH_4Cl (9.5 g, 177.6 mmol, 50 equiv.) was added over at least 2 h. After the mixture turned white and pasty, liquid NH_3 was removed via a stream of nitrogen. The resulting residue was added H_2O (300 mL) and extracted with EtOAc (3 × 100 mL). The organic layer was washed with brine (100 mL), dried over Na_2SO_4, and concentrated. The residue was subjected to flash chromatography (60% EtOAc in hexane) to give **14** (970 mg, 2.2 mmol, 61%) as a colorless solid. ^{1}H NMR (400 MHz, Chloroform-*d*) δ 5.60 – 5.39 (m, 1H), 3.92 (tt, *J* = 11.2, 5.1 Hz, 1H), 3.79 (s, 1H), 3.73 – 3.55 (m, 1H), 2.41 – 2.16 (m, 2H), 2.16 – 2.06 (m, 1H), 2.06 – 1.99 (m, 1H), 1.96 – 1.83 (m, 1H), 1.73 – 1.61 (m, 1H), 1.61 – 1.25 (m, 8H), 1.25 – 1.16 (m, 1H), 1.16 – 1.04 (m, 2H), 1.01 (dd, *J* = 6.1, 1.4 Hz, 4H), 0.97 (d, *J* = 1.4 Hz, 3H), 0.82 (d, *J* = 1.5 Hz, 9H), 0.64 (d, *J* = 1.4 Hz, 3H), 0.00 (d, *J* = 1.7 Hz, 6H). ^{13}C NMR (101 MHz, $CDCl_3$) δ 137.39, 125.56, 72.98, 70.99, 66.47, 58.26, 56.30, 42.05, 41.84, 41.76, 41.41, 39.02, 38.22, 31.85, 31.78, 26.09, 25.74, 24.48, 23.78, 20.08, 19.50, 18.09, 12.05, -3.41, -4.02. HRMS (ESI+) m/z 471.3273 [M + Na]+ (error: 0.6 ppm).

1.12. Synthesis of 15.

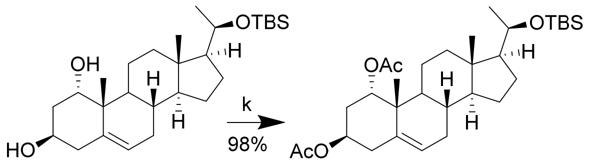

14 **15**

(1S,3R,8S,10R,13S,14S,17S)-17-((R)-1-((tert-butyldimethylsilyl)oxy)ethyl)-10,13-dimethyl-2,3,4,7,8,9,10,11,12,13,14,15,16,17-tetradecahydro-1H-cyclopenta[a]phenanthrene-1,3-diyl diacetate. To a solution of alcohol **14** (725 mg, 1.6 mmol) in DCM (20 mL) was added Ac_2O (1.6 g, 16.2 mmol, 10 equiv.), Et_3N (3.4 g, 32.4 mmol, 20 equiv.) and catalytic DMAP (0.05 equiv.). The reaction mixture was stirred at r.t. for overnight. The solvents were removed under reduced pressure, the resulting mixture was subjected to flash chromatography (10% EtOAc in hexane) to give **15** (843.7 mg, 1.6 mmol, 98%) as a white solid, which was further crystalized in hexane to determine its absolute structure. ^{1}H NMR (400 MHz, Chloroform-*d*) δ 5.58 – 5.49 (m, 1H), 5.06 (d, *J* = 3.0 Hz, 1H), 4.92 (tt, *J* = 11.2, 4.9 Hz, 1H), 3.72 (dt, *J* = 11.8, 5.9 Hz, 1H), 2.48 (dd, *J* = 13.7, 5.2 Hz, 1H), 2.42 – 2.28 (m, 1H), 2.21 – 2.08 (m, 3H), 2.06 (s, 3H), 2.03 (s, 3H), 2.01 – 1.91 (m, 2H), 1.91 – 1.75 (m, 1H), 1.62 (s, 3H), 1.57 – 1.23 (m, 6H), 1.23 – 1.11 (m, 1H), 1.11 (s, 3H), 1.08 (d, *J* = 5.7 Hz, 3H), 0.89 (s, 9H), 0.70 (s, 3H), 0.06 (s, 6H). ^{13}C NMR (101 MHz, $CDCl_3$) δ 170.64, 170.59, 136.39, 125.35, 77.43, 74.91, 71.27, 69.66, 58.40, 56.55, 42.35, 42.26, 40.66, 39.23, 37.55, 32.09, 31.96, 31.82, 26.33, 26.04, 24.65, 24.06, 21.55, 21.35, 20.35, 19.66, 18.31, 12.22, -3.31, -3.81. MS (ESI) m/z 555.4 $[M + Na]^+$.

1.13. Synthesis of 16.

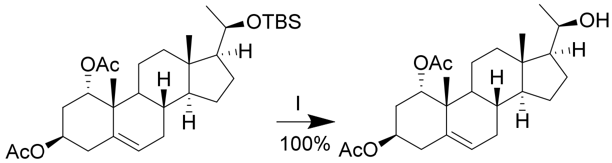

15 **16**

(1S,3R,8S,10R,13S,14S,17S)-17-((R)-1-hydroxyethyl)-10,13-dimethyl-2,3,4,7,8,9,10,11,12,13,14,15,16,17-tetradecahydro-1H-cyclopenta[a]phenanthrene-1,3-diyl diacetate. To a solution of ester **15** (437 mg, 0.8 mmol) in THF (5 mL) was added TBAF (1.0 M in THF, 1.6 mL, 2.0 equiv.) and stirred at room temperature for 48 h. The reaction mixture was quenched with sat. $NaHCO_3$ (100 mL), then extracted with ethyl acetate (3 × 20 mL). The combined organic layer was washed with brine (20 mL) and H_2O (20 mL), dried over Na_2SO_4. The crude mixture after removing solvents under reduced pressure was subjected to flash chromatography (30% ethyl acetate in hexane) to give an alcohol with quantitative yield. ^{1}H NMR (400 MHz, Chloroform-*d*) δ 5.47 (dd, *J* = 5.5, 2.4 Hz, 1H), 4.99 (d, *J* = 3.1 Hz, 1H), 4.85 (tt, *J* = 11.4, 5.2 Hz, 1H), 3.66 (s, 1H), 2.49 – 2.36 (m, 1H), 2.28 (t, *J* = 13.0 Hz, 1H), 2.08 – 2.00 (m, 2H), 1.99 (d, *J* = 2.0 Hz, 3H), 1.96 (d, *J* = 2.0 Hz, 3H), 1.94 – 1.85 (m, 1H), 1.77 (t, *J* = 12.8 Hz, 1H), 1.70 – 1.37 (m, 5H), 1.37 – 1.10 (m, 5H), 1.07 (dd, *J* = 6.3, 1.9 Hz, 3H), 1.03 (d, *J* = 2.0 Hz, 3H), 1.01 – 0.93 (m, 1H), 0.82 (d, *J* = 2.1 Hz, 1H), 0.70 (s, 3H). ^{13}C NMR (101 MHz, $CDCl_3$) δ 170.38, 170.31, 136.15, 125.03, 74.61, 70.46, 69.40, 58.46, 56.16, 42.25, 42.14, 40.42, 39.68, 37.33, 31.91, 31.67, 31.54, 25.58, 24.52, 23.72, 21.34, 21.10, 20.24, 19.44, 12.37. MS (ESI) m/z 441.4 $[M + Na]^+$.

1.14. Synthesis of 3.

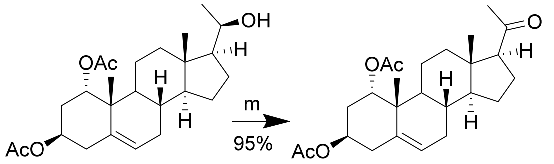

16 **3**

(1S,3R,8S,10R,13S,14S,17S)-17-acetyl-10,13-dimethyl-2,3,4,7,8,9,10,11,12,13,14,15,16,17-tetradecahydro-1H-cyclopenta[a]phenanthrene-1,3-diyl diacetate. To a DCM (10 mL) solution of **16** (200 mg, 0.5 mmol) was added dess–martin periodinane (811 mg, 1.9 mmol, 4.0 equiv.) and stirred at room temperature for 12 h. The reaction mixture was quenched with sat. $NaHCO_3$ (30 mL) and stirred for 1 h, then extracted with DCM (3 × 20 mL). The combined organic layer was washed with brine (10 mL) and H2O (10 mL), dried over Na_2SO_4. The crude mixture after removing solvents under reduced pressure was subjected to flash chromatography (30% EtOAc in hexane) to give **3** (189 mg, 0.45 mmol, 95%) as a white solid. ^{1}H NMR (400 MHz, Chloroform-*d*) δ 5.47 (dd, *J* = 5.4, 2.4 Hz, 1H), 5.01 (d, *J* = 3.2 Hz, 1H), 4.86 (tt, *J* = 11.6, 5.1 Hz, 1H), 2.52 – 2.35 (m, 2H), 2.28 (t, *J* = 12.6 Hz, 1H), 2.12 (q, *J* = 11.4, 10.9 Hz, 1H), 2.04 (s, 3H), 2.00 (s, 3H), 1.96 (s, 3H), 1.92 (d, *J* = 2.8 Hz, 2H), 1.77 (t, *J* = 12.8 Hz, 1H), 1.69 – 1.04 (m, 10H), 1.02 (s, 3H),

0.92 – 0.72 (m, 1H), 0.55 (s, 3H). ^{13}C NMR (101 MHz, $CDCl_3$) δ 209.34, 170.39, 170.34, 136.03, 124.91, 74.52, 69.30, 63.60, 56.85, 43.94, 42.03, 40.43, 38.69, 37.25, 31.92, 31.67, 31.56, 31.50, 24.45, 22.75, 21.33, 21.15, 20.43, 19.41, 13.25. MS (ESI) m/z 439.5 $[M + Na]^+$.

1.15. Synthesis of 17.

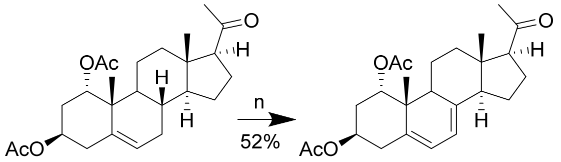

3 **17**

(1S,3R,10R,13S,14R,17S)-17-acetyl-10,13-dimethyl-2,3,4,9,10,11,12,13,14,15,16,17-dodecahydro-1H-cyclopenta[a]phenanthrene-1,3-diyl diacetate. To a solution of compound **3** (170 mg, 0.41 mmol) in sodium-dried benzene–hexane (10 mL, 1:1) was added dibromantin (70 mg, 0.25 mmol, 0.6 equiv.) and AIBN (2.7 mg, 0.02 mmol, 0.04 equiv.). The mixture was refluxed for 20 min in a preheated oil bath (100 ºC) and then placed in an ice bath to cool. Insoluble material was removed by filtration and the filtrate was concentrated to yield a white solid. To a solution of this solid in tetrahydrofuran (10 mL) was added tetrabutylammonium bromide (33 mg, 0.1 mmol, 0.25 equiv.) and stirred for 75 min at r.t. To this reaction mixture was added tetrabutylammonium fluoride (0.8 mL of 1.0 M solution in THF, 2 equiv.) and the resulting solution was stirred for 50 min in the dark. Sat. Na_2CO_3 solution (30 mL) was added and stirred for 30 min. The mixture was extracted by ethyl acetate (3 × 30 mL). The organic layer was dried and concentrated. The residual was subjected to flash chromatography (20% EtOAc in hexane) to give **17** (88 mg, 0.21 mmol, 52%) as a white solid. ^{1}H NMR (400 MHz, Methanol-d_4) δ 5.73 – 5.66 (m, 1H), 5.44 (dd, *J* = 5.7, 2.8 Hz, 1H), 5.03 – 4.99 (m, 1H), 4.96 (q, *J* = 6.0 Hz, 1H), 2.73 (t, *J* = 8.9 Hz, 1H), 2.63 (dd, *J* = 14.7, 5.0 Hz, 1H), 2.57 – 2.35 (m, 2H), 2.21 (t, *J* = 9.8 Hz, 1H), 2.16 (s, 3H), 2.11 (s, 3H), 2.04 (s, 3H), 2.01 – 1.63 (m, 4H), 1.54 (q, *J* = 12.0, 8.9 Hz, 3H), 1.46 – 1.06 (m, 2H), 1.03 (s, 3H), 1.01 – 0.79 (m, 1H), 0.58 (s, 3H). ^{13}C NMR (101 MHz, MeOD) δ 212.25, 172.51, 172.14, 140.91, 136.82, 122.81, 117.73, 75.91, 69.96, 64.34, 55.72, 45.52, 42.39, 39.24, 39.16, 36.83, 33.09, 32.12, 24.41, 23.89, 21.78, 21.70, 21.52, 16.83, 14.02. MS (ESI) m/z 437.5 $[M + Na]^+$.

1.16. Synthesis of 18.

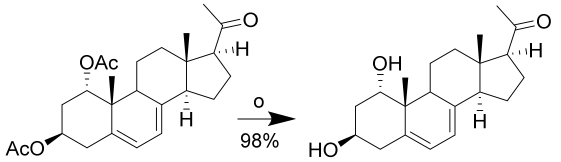

17 **18**

1-((1S,3R,10R,13S,14R,17S)-1,3-dihydroxy-10,13-dimethyl-2,3,4,9,10,11,12,13,14,15,16,17-dodecahydro-1H-cyclopenta[a]phenanthren-17-yl)ethan-1-one. To a stirred solution of **17** (20 mg, 0.048 mmol) in methanol (2 mL) was added K_2CO_3 (33 mg, 0.24 mmol, 5 equiv.). The reaction mixture was stirred overnight at r.t. The reaction mixture was dried and subjected to flash chromatography directly (hexane:EtOAc:MeOH=60:35:5) to give **18** as a white solid (16 mg, 0.047 mmol, 98%). ^{1}H NMR (400 MHz, Methanol-d_4) δ 5.68 – 5.63 (m, 1H), 5.41 (dd, *J* = 5.7, 2.8 Hz, 1H), 3.99 (dq, *J* = 11.2, 5.8, 4.9 Hz, 1H), 3.75 (d, *J* = 3.2 Hz, 1H), 2.89 (t, *J* = 9.7 Hz, 1H), 2.78 (t, *J* = 8.8 Hz, 1H), 2.54 – 2.41 (m, 1H), 2.38 – 2.20 (m, 1H), 2.17 (s, 3H), 2.15 – 2.05 (m, 2H), 1.96 – 1.66 (m, 4H), 1.66 – 1.48 (m, 2H), 1.31 (s, 3H), 0.94 (s, 3H), 0.60 (s, 3H). ^{13}C NMR (101 MHz, MeOD) δ 212.11, 141.09, 139.17, 122.04, 117.30, 73.09, 65.79, 64.25, 55.72, 45.51, 43.29, 40.94, 39.41, 39.31, 38.70, 31.60, 24.37, 23.74, 21.44, 16.74, 13.56. MS (ESI) m/z 353.4 $[M + Na]^+$.

1.17. Synthesis of 2.

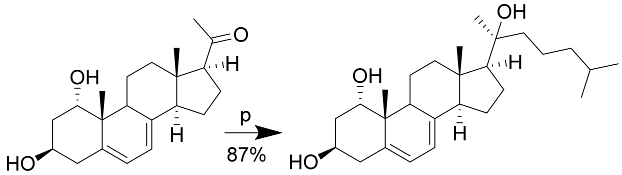

18 **2**

(1S,3R,10R,13S,14R,17S)-17-(2-hydroxy-6-methylheptan-2-yl)-10,13-dimethyl-2,3,4,9,10,11,12,13,14,15,16,17-dodecahydro-1H-cyclopenta[a]phenanthrene-1,3-diol. To a flame dried flask (25 mL) containing Mg (324 mg, 135.5 mmol, 2 equiv.) and catalytic I2 was added sodium dried THF (10 mL) and 1-bromo-4-methylpentane (1.1 g, 6.7 mmol, 1 equiv.). Under argon protection, the reaction mixture was stirred under reflux for 1 h, and cooled to 0 ºC.

Then the self-made Grignard reagent (0.35 mL, 0.235 mmol, 5 equiv.) was added to **18** (16 mg, 0.047 mmol, 1.0 equiv.) pre-dissolved in anhydrous THF (3 mL) under 0 ºC. The reaction mixture was allowed to warm to r.t., stirred overnight, quenched with sat. NH_4Cl (10 mL), and extracted with ethyl acetate (3 × 5 mL). The organic layer was combined and washed with sat. $NaHCO_3$ (3 mL), brine (3 mL) and water (3 mL), dried over anhydrous Na_2SO_4, and concentrated. The crude mixture was subjected to flash chromatography (hexane:EtOAc:MeOH=60:35:5) to give product **2** (17 mg, 0.041 mmol, 87%) as a colorless sticky solid. ^{1}H NMR (400 MHz, Acetone-d_6) δ 5.60 (d, J = 5.7 Hz, 1H), 5.35 (dd, J = 5.9, 2.9 Hz, 1H), 4.03 (d, J = 6.1 Hz, 1H), 3.76 (s, 1H), 3.72 (d, J = 2.5 Hz, 1H), 3.58 – 3.49 (m, 1H), 2.92 (s, 2H), 2.44 (dd, J = 15.2, 5.1 Hz, 1H), 2.24 (dt, J = 38.5, 11.7 Hz, 2H), 2.02 – 1.84 (m, 2H), 1.84 – 1.51 (m, 7H), 1.51 – 1.30 (m, 5H), 1.28 (s, 3H), 1.17 (td, J = 11.3, 9.3, 4.6 Hz, 4H), 0.92 (s, 3H), 0.89 (dd, J = 6.9, 2.5 Hz, 6H), 0.85 (s, 3H). ^{13}C NMR (101 MHz, Acetone) δ 141.86, 139.37, 121.14, 116.21, 74.60, 72.59, 65.01, 58.67, 55.84, 45.16, 43.94, 42.88, 41.44, 40.59, 40.53, 40.02, 38.16, 28.67, 26.96, 23.47, 23.04, 23.00, 22.89, 22.73, 21.05, 16.63, 14.07. HRMS (ESI+) m/z 399.3265 [M + Na - H2O]+ (error: 0.5 ppm).

1.18. Synthesis of 1.

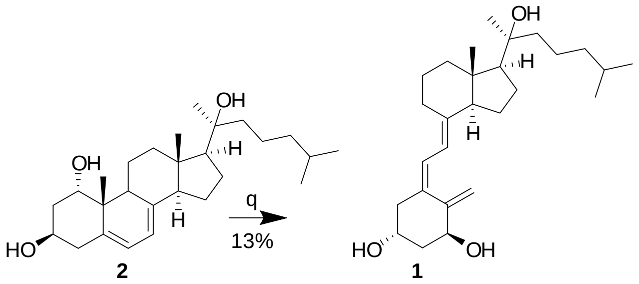

(1R,3S,Z)-5-(2-((1S,3aS,7aS,E)-1-(2-hydroxy-6-methylheptan-2-yl)-7a-methyloctahydro-4H-inden-4-ylidene)ethylidene)-4-methylenecyclohexane-1,3-diol. A ethyl ether solution of **2** (17 mg, 1 mg/mL) was purged with argon gas for 10 min and subjected to UVB irradiation for 15 min in a quartz tube with a temperature below 50 °C, using a Rayonet RPR-100 photochemical reactor (Branford, CT). The compound was protected from light and sit in the dark at r.t. for 10 days to allow the conversion from pre-VD3 to VD3. The mixture was dried, dissolved in EtOH, and analyzed by an Agilent 1100 HPLC system (Santa Clara, CA) to obtain an optimized the analytical condition (50-100% MeCN, 0-30 min). The separation was carried out using a preparative HPLC system. The reaction mixture (500 µL) was injected by an autosampler onto a 5 µm Phenomenex Luna-PFP column (250 mm × 21.2 mm) (Torrance, CA) with mobile phase following the gradient (0-30 min, 40-70% MeCN) at a flow rate of 15 mL/min. Fractions containing product was monitored by UV absorbance, collected, and dried by lyophilizer (2.2 mg, 13%). ^{1}H NMR (400 MHz, Methanol-d_4) δ 6.34 (d, J = 11.1 Hz, 1H), 6.10 (d, J = 11.2 Hz, 1H), 5.31 (s, 1H), 4.92 (s, 3H), 4.38 (d, J = 6.3 Hz, 1H), 4.15 (s, 1H), 2.88 (d, J = 12.7 Hz, 1H), 2.59 – 2.46 (m, 1H), 2.28 (dd, J = 13.4, 6.7 Hz, 1H), 2.17 – 1.96 (m, 2H), 1.90 (d, J = 6.1 Hz, 2H), 1.85 – 1.28 (m, 11H), 1.26 (s, 3H), 1.19 (t, J = 7.2 Hz, 3H), 0.91 (dd, J = 6.6, 2.0 Hz, 6H), 0.73 (s, 3H). HRMS (ESI+) m/z 399.3260 [M + Na - H2O]+ (error: -0.7 ppm).

2. Identification of 1α-OH in 15 by NOESY.

Strong NOE effects of 1β-H/19-Me and 1β-H/2β-H were observed as shown below, suggesting that H (β) on C1 is adjacent to both 19-Me and 2β-H while an α-H would make such strong NOE effects impossible.

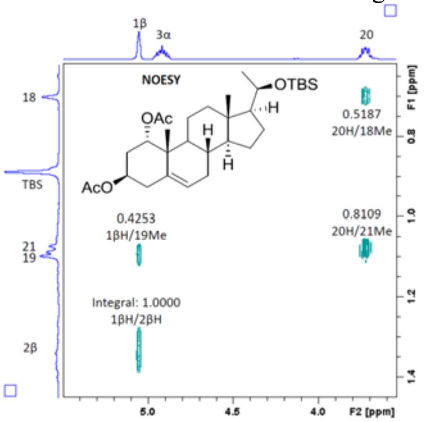

Supplementary Figure S1. Partial NOESY spectrum of intermediate **15**.

3. Crystal structure analysis of 15.

A colorless, needle-like specimen of **15**, approximate dimensions 0.438 mm x 0.050 mm x 0.015 mm, was cleaved from a longer needle. The selected crystal was affixed to a MiTeGen sample support with low-viscosity cryo oil, and flash cooled to 100 K for x-ray analysis on a Bruker X8 κ diffractometer.

The crystal was illuminated with the X-ray beam from a Bruker IμSCu microfocus sealed tube, and the diffraction data were measured with a Bruker Photon-100 detector. The resulting images were integrated with the SAINT software package using a narrow-frame algorithm. Integration of the data based on a monoclinic unit cell yielded a total of 22378 reflections to a maximum θ angle of 68.716° (0.83 Å resolution), of which 5512 were independent and 4649 (84.3%) were considered observed. The data were complete (100.0%) to a θ angle of 67.679° (0.83 Å). A numerical absorption correction was applied, and data were corrected for inter-frame scaling differences, via program SADABS. The structure was solved and refined via SHELXT and SHELXL-2014, using the space group $P2_1$, with $Z = 2$ for the formula unit, $C_{31}H_{52}O_5Si$ (one molecule of **15**). Hydrogen atoms were placed in idealized positions, and refined according to a riding model. The final anisotropic full-matrix least-squares refinement converged at R1 = 5.96% for the observed data and wR2 = 12.80% for all data. The largest peak in the final difference electron density synthesis was 0.381 $e^{-}/Å^3$ and the deepest hole was −0.299 $e^{-}/Å^3$, with an RMS deviation of 0.052 $e^{-}/Å^3$.

The structure contains one unique molecule per unit cell, with nine distinct stereocenters. The data support high confidence in the reported absolute structure, as judged by a refined Flack parameter value of 0.02(5).

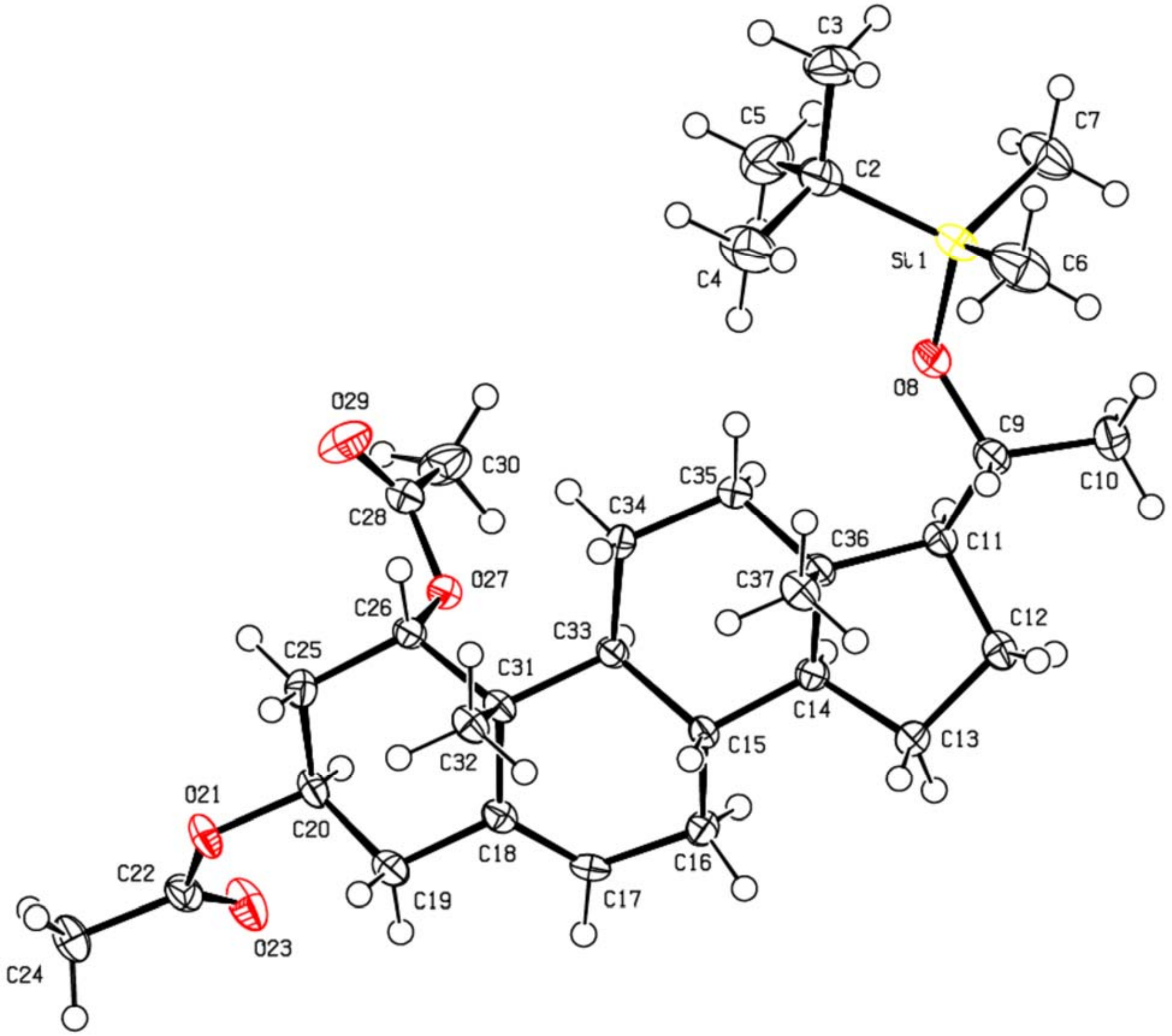

Supplementary Figure S2. Labeled thermal ellipsoid plot and numbering of intermediate **15** for X-ray analysis.

Supplementary Table S1. Chemical and Crystal Data.

Chemical formula	$C_{31}H_{52}O_5Si$
Formula weight, Da	532.81
Temperature, K	100(2)
Crystal description	colorless needle
Crystal size, mm	0.438 x 0.050 x 0.015
Crystal system	monoclinic
Space group	$P2_1$
Unit cell dimensions, from 9910 reflections having 2.27° ≤ θ ≤ 68.51°	
a, Å	12.3062(5)
b, Å	6.4738(3)
c, Å	19.5638(8)
β, °	95.760(3)
Volume, $Å^3$	1550.73(11)
Z	2
Density (calculated), g/cm^3	1.141
Absorption coefficient, mm^{-1}	0.943
F(000)	584

Supplementary Table S2. Data Collection, Structure Solution, and Refinement

X-ray wavelength, Å	1.54178
Theta range for data integration	3.610 to 68.716°
Index ranges	$-14 \leq h \leq 14$, $-7 \leq k \leq 6$, $-23 \leq l \leq 23$
Reflections collected	22378
Independent reflections	5512 (R_{int} = 0.0853)
Observed reflections (I > 2σ(I))	4649
Coverage of independent reflections	99.9%
Absorption correction method	numerical
Max. and min. transmission	1.0000 and 0.7154
Structure solution technique	dual-space method
Structure solution program	SHELXT (Sheldrick 2014)
Refinement method	full-matrix least-squares on F^2
Refinement program	SHELXL-2014/7 (Sheldrick, 2014)
Function minimized	$\Sigma\, w(F_o^2 - F_c^2)^2$
Weighting scheme	$w=1/[\sigma^2(F_o^2)+(0.0480P)^2+1.2000P]$ where $P=(F_o^2+2F_c^2)/3$
Data / restraints / parameters	5512 / 1 / 335
Goodness-of-fit on F^2	1.054
Final residuals	
observed data	*R1* = 0.0596, *wR2* = 0.1202
all data	*R1* = 0.0735, *wR2* = 0.1280
Flack parameter	0.02(5)
Largest difference peak and hole, $e^-/Å^3$	0.381 and −0.299
R.M.S. deviation from mean, $e^-/Å^3$	0.052

Supplementary Table S3. Atomic Coordinates and Equivalent Isotropic Displacement Parameters* (Å^2).

Atom	*x/a*	*y/b*	*z/c*	*U(eq)*
Si1	−0.33836(10)	1.1674(2)	0.91146(6)	0.0235(3)
C2	−0.1989(4)	1.2300(9)	0.9551(3)	0.0347(13)
C3	−0.2085(5)	1.3670(10)	1.0188(3)	0.0476(16)
C4	−0.1336(5)	1.3470(11)	0.9047(3)	0.0558(19)
C5	−0.1386(5)	1.0314(11)	0.9781(3)	0.0552(18)
C6	−0.4096(5)	1.4101(9)	0.8805(3)	0.0429(14)
C7	−0.4202(4)	1.0258(9)	0.9715(3)	0.0379(13)
O8	−0.3156(2)	1.0190(5)	0.84557(16)	0.0249(7)
C9	−0.3811(3)	0.9993(7)	0.7817(2)	0.0226(10)
C10	−0.4993(3)	0.9419(8)	0.7923(3)	0.0307(12)
C11	−0.3291(3)	0.8377(7)	0.7386(2)	0.0190(9)
C12	−0.3944(3)	0.8047(8)	0.6671(2)	0.0237(10)
C13	−0.3100(3)	0.7556(7)	0.6160(2)	0.0221(10)
C14	−0.2044(3)	0.7187(7)	0.6626(2)	0.0177(9)
C15	−0.0980(3)	0.7152(6)	0.6294(2)	0.0156(9)
C16	−0.0973(3)	0.5406(7)	0.5768(2)	0.0188(9)
C17	0.0148(3)	0.4907(7)	0.5581(2)	0.0184(9)
C18	0.1072(3)	0.5636(7)	0.5901(2)	0.0179(9)
C19	0.2154(3)	0.5154(7)	0.5630(2)	0.0222(10)
C20	0.2996(3)	0.4454(7)	0.6201(2)	0.0205(10)
O21	0.4063(2)	0.4235(5)	0.59453(16)	0.0232(7)
C22	0.4299(4)	0.2381(7)	0.5688(2)	0.0221(10)
O23	0.3697(3)	0.0923(5)	0.56697(18)	0.0317(8)
C24	0.5408(4)	0.2399(7)	0.5433(3)	0.0258(11)
C25	0.3133(3)	0.6082(7)	0.6762(2)	0.0203(10)
C26	0.2059(3)	0.6442(7)	0.7066(2)	0.0176(9)
O27	0.1781(2)	0.4538(5)	0.74147(14)	0.0197(7)
C28	0.2238(4)	0.4286(8)	0.8060(2)	0.0248(10)
O29	0.2837(3)	0.5550(6)	0.83612(17)	0.0377(9)
C30	0.1912(4)	0.2281(8)	0.8356(3)	0.0365(13)
C31	0.1094(3)	0.7044(6)	0.6532(2)	0.0166(9)
C32	0.1317(3)	0.9284(7)	0.6309(2)	0.0210(10)
C33	−0.0009(3)	0.6866(7)	0.6850(2)	0.0161(8)
C34	−0.0068(3)	0.8277(7)	0.7481(2)	0.0210(10)
C35	−0.1190(3)	0.8230(7)	0.7766(2)	0.0203(10)
C36	−0.2105(3)	0.8767(7)	0.7209(2)	0.0164(9)
C37	−0.1979(4)	1.1005(7)	0.6964(2)	0.0223(10)

* U(eq) is defined as one third of the trace of the orthogonalized U_{ij} tensor.

Supplementary Table S4. Bond lengths (Å).

Si1-O8	1.654(3)	Si1-C7	1.864(5)
Si1-C6	1.870(5)	Si1-C2	1.882(5)

C2-C5	1.530(8)	C2-C4	1.533(7)
C2-C3	1.544(7)	O8-C9	1.423(5)
C9-C11	1.525(6)	C9-C10	1.535(6)
C11-C36	1.553(5)	C11-C12	1.557(6)
C12-C13	1.545(6)	C13-C14	1.531(6)
C14-C15	1.518(5)	C14-C36	1.540(6)
C15-C16	1.529(6)	C15-C33	1.545(5)
C16-C17	1.498(6)	C17-C18	1.328(6)
C18-C19	1.514(6)	C18-C31	1.533(6)
C19-C20	1.516(6)	C20-O21	1.458(5)
C20-C25	1.518(6)	O21-C22	1.345(5)
C22-O23	1.198(5)	C22-C24	1.498(6)
C25-C26	1.520(5)	C26-O27	1.466(5)
C26-C31	1.550(6)	O27-C28	1.339(5)
C28-O29	1.213(6)	C28-C30	1.493(7)
C31-C32	1.547(6)	C31-C33	1.553(5)
C33-C34	1.543(6)	C34-C35	1.540(6)
C35-C36	1.526(6)	C36-C37	1.539(6)
C3-H3A	0.9800	C3-H3B	0.9800
C3-H3C	0.9800	C4-H4A	0.9800
C4-H4B	0.9800	C4-H4C	0.9800
C5-H5A	0.9800	C5-H5B	0.9800
C5-H5C	0.9800	C6-H6A	0.9800
C6-H6B	0.9800	C6-H6C	0.9800
C7-H7A	0.9800	C7-H7B	0.9800
C7-H7C	0.9800	C9-H9A	1.0000
C10-H10A	0.9800	C10-H10B	0.9800
C10-H10C	0.9800	C11-H11A	1.0000
C12-H12A	0.9900	C12-H12B	0.9900
C13-H13A	0.9900	C13-H13B	0.9900
C14-H14A	1.0000	C15-H15A	1.0000
C16-H16A	0.9900	C16-H16B	0.9900
C17-H17A	0.9500	C19-H19A	0.9900
C19-H19B	0.9900	C20-H20A	1.0000
C24-H24A	0.9800	C24-H24B	0.9800
C24-H24C	0.9800	C24-H24D	0.9800
C24-H24E	0.9800	C24-H24F	0.9800
C25-H25A	0.9900	C25-H25B	0.9900
C26-H26A	1.0000	C30-H30A	0.9800
C30-H30B	0.9800	C30-H30C	0.9800
C30-H30D	0.9800	C30-H30E	0.9800
C30-H30F	0.9800	C32-H32A	0.9800
C32-H32B	0.9800	C32-H32C	0.9800
C33-H33A	1.0000	C34-H34A	0.9900

C34-H34B	0.9900	C35-H35A	0.9900
C35-H35B	0.9900	C37-H37A	0.9800
C37-H37B	0.9800	C37-H37C	0.9800

Supplementary Table S5. Bond angles (°).

O8-Si1-C7	110.5(2)	O8-Si1-C6	110.2(2)
C7-Si1-C6	110.6(3)	O8-Si1-C2	105.10(19)
C7-Si1-C2	110.2(2)	C6-Si1-C2	110.0(3)
C5-C2-C4	109.5(5)	C5-C2-C3	108.6(5)
C4-C2-C3	108.8(5)	C5-C2-Si1	110.2(4)
C4-C2-Si1	109.1(4)	C3-C2-Si1	110.5(4)
C9-O8-Si1	127.7(3)	O8-C9-C11	108.4(3)
O8-C9-C10	111.4(4)	C11-C9-C10	111.4(4)
C9-C11-C36	117.9(4)	C9-C11-C12	112.6(3)
C36-C11-C12	103.6(3)	C13-C12-C11	106.8(3)
C14-C13-C12	103.4(3)	C15-C14-C13	117.6(3)
C15-C14-C36	115.8(3)	C13-C14-C36	103.7(3)
C14-C15-C16	111.3(3)	C14-C15-C33	109.8(3)
C16-C15-C33	109.2(3)	C17-C16-C15	113.0(3)
C18-C17-C16	125.0(4)	C17-C18-C19	120.2(4)
C17-C18-C31	122.4(4)	C19-C18-C31	117.3(4)
C18-C19-C20	111.4(3)	O21-C20-C19	110.4(3)
O21-C20-C25	106.1(3)	C19-C20-C25	110.1(4)
C22-O21-C20	116.9(3)	O23-C22-O21	123.9(4)
O23-C22-C24	125.3(4)	O21-C22-C24	110.9(4)
C20-C25-C26	110.6(3)	O27-C26-C25	107.8(3)
O27-C26-C31	109.0(3)	C25-C26-C31	114.3(3)
C28-O27-C26	116.4(3)	O29-C28-O27	123.6(4)
O29-C28-C30	124.8(4)	O27-C28-C30	111.6(4)
C18-C31-C32	108.6(3)	C18-C31-C26	110.1(3)
C32-C31-C26	106.3(3)	C18-C31-C33	109.4(3)
C32-C31-C33	111.7(4)	C26-C31-C33	110.7(3)
C34-C33-C15	113.4(3)	C34-C33-C31	112.9(3)
C15-C33-C31	110.8(3)	C35-C34-C33	113.2(3)
C36-C35-C34	111.2(3)	C35-C36-C37	110.3(4)
C35-C36-C14	106.8(3)	C37-C36-C14	112.4(3)
C35-C36-C11	116.6(3)	C37-C36-C11	110.4(4)
C14-C36-C11	100.0(3)		
C2-C3-H3A	109.5	C2-C3-H3B	109.5
H3A-C3-H3B	109.5	C2-C3-H3C	109.5
H3A-C3-H3C	109.5	H3B-C3-H3C	109.5
C2-C4-H4A	109.5	C2-C4-H4B	109.5
H4A-C4-H4B	109.5	C2-C4-H4C	109.5
H4A-C4-H4C	109.5	H4B-C4-H4C	109.5
C2-C5-H5A	109.5	C2-C5-H5B	109.5

H5A-C5-H5B	109.5	C2-C5-H5C	109.5
H5A-C5-H5C	109.5	H5B-C5-H5C	109.5
Si1-C6-H6A	109.5	Si1-C6-H6B	109.5
H6A-C6-H6B	109.5	Si1-C6-H6C	109.5
H6A-C6-H6C	109.5	H6B-C6-H6C	109.5
Si1-C7-H7A	109.5	Si1-C7-H7B	109.5
H7A-C7-H7B	109.5	Si1-C7-H7C	109.5
H7A-C7-H7C	109.5	H7B-C7-H7C	109.5
O8-C9-H9A	108.6	C11-C9-H9A	108.6
C10-C9-H9A	108.6	C9-C10-H10A	109.5
C9-C10-H10B	109.5	H10A-C10-H10B	109.5
C9-C10-H10C	109.5	H10A-C10-H10C	109.5
H10B-C10-H10C	109.5	C9-C11-H11A	107.4
C36-C11-H11A	107.4	C12-C11-H11A	107.4
C13-C12-H12A	110.4	C11-C12-H12A	110.4
C13-C12-H12B	110.4	C11-C12-H12B	110.4
H12A-C12-H12B	108.6	C14-C13-H13A	111.1
C12-C13-H13A	111.1	C14-C13-H13B	111.1
C12-C13-H13B	111.1	H13A-C13-H13B	109.0
C15-C14-H14A	106.3	C13-C14-H14A	106.3
C36-C14-H14A	106.3	C14-C15-H15A	108.8
C16-C15-H15A	108.8	C33-C15-H15A	108.8
C17-C16-H16A	109.0	C15-C16-H16A	109.0
C17-C16-H16B	109.0	C15-C16-H16B	109.0
H16A-C16-H16B	107.8	C18-C17-H17A	117.5
C16-C17-H17A	117.5	C18-C19-H19A	109.4
C20-C19-H19A	109.4	C18-C19-H19B	109.4
C20-C19-H19B	109.4	H19A-C19-H19B	108.0
O21-C20-H20A	110.1	C19-C20-H20A	110.1
C25-C20-H20A	110.1	C22-C24-H24A	109.5
C22-C24-H24B	109.5	H24A-C24-H24B	109.5
C22-C24-H24C	109.5	H24A-C24-H24C	109.5
H24B-C24-H24C	109.5	C22-C24-H24D	109.5
H24A-C24-H24D	141.1	H24B-C24-H24D	56.3
H24C-C24-H24D	56.3	C22-C24-H24E	109.5
H24A-C24-H24E	56.3	H24B-C24-H24E	141.1
H24C-C24-H24E	56.3	H24D-C24-H24E	109.5
C22-C24-H24F	109.5	H24A-C24-H24F	56.3
H24B-C24-H24F	56.3	H24C-C24-H24F	141.1
H24D-C24-H24F	109.5	H24E-C24-H24F	109.5
C20-C25-H25A	109.5	C26-C25-H25A	109.5
C20-C25-H25B	109.5	C26-C25-H25B	109.5
H25A-C25-H25B	108.1	O27-C26-H26A	108.5
C25-C26-H26A	108.5	C31-C26-H26A	108.5

C28-C30-H30A	109.5	C28-C30-H30B	109.5
H30A-C30-H30B	109.5	C28-C30-H30C	109.5
H30A-C30-H30C	109.5	H30B-C30-H30C	109.5
C28-C30-H30D	109.5	H30A-C30-H30D	141.1
H30B-C30-H30D	56.3	H30C-C30-H30D	56.3
C28-C30-H30E	109.5	H30A-C30-H30E	56.3
H30B-C30-H30E	141.1	H30C-C30-H30E	56.3
H30D-C30-H30E	109.5	C28-C30-H30F	109.5
H30A-C30-H30F	56.3	H30B-C30-H30F	56.3
H30C-C30-H30F	141.1	H30D-C30-H30F	109.5
H30E-C30-H30F	109.5	C31-C32-H32A	109.5
C31-C32-H32B	109.5	H32A-C32-H32B	109.5
C31-C32-H32C	109.5	H32A-C32-H32C	109.5
H32B-C32-H32C	109.5	C34-C33-H33A	106.4
C15-C33-H33A	106.4	C31-C33-H33A	106.4
C35-C34-H34A	108.9	C33-C34-H34A	108.9
C35-C34-H34B	108.9	C33-C34-H34B	108.9
H34A-C34-H34B	107.8	C36-C35-H35A	109.4
C34-C35-H35A	109.4	C36-C35-H35B	109.4
C34-C35-H35B	109.4	H35A-C35-H35B	108.0
C36-C37-H37A	109.5	C36-C37-H37B	109.5
H37A-C37-H37B	109.5	C36-C37-H37C	109.5
H37A-C37-H37C	109.5	H37B-C37-H37C	109.5

Supplementary Table S6. Torsion angles (°).

O8-Si1-C2-C5	−59.2(4)	C7-Si1-C2-C5	59.9(4)
C6-Si1-C2-C5	−177.9(4)	O8-Si1-C2-C4	61.1(4)
C7-Si1-C2-C4	−179.8(4)	C6-Si1-C2-C4	−57.5(5)
O8-Si1-C2-C3	−179.2(4)	C7-Si1-C2-C3	−60.1(5)
C6-Si1-C2-C3	62.1(4)	C7-Si1-O8-C9	90.1(4)
C6-Si1-O8-C9	−32.5(4)	C2-Si1-O8-C9	−151.0(4)
Si1-O8-C9-C11	−177.8(3)	Si1-O8-C9-C10	−54.9(5)
O8-C9-C11-C36	−59.2(5)	C10-C9-C11-C36	177.9(4)
O8-C9-C11-C12	−179.8(3)	C10-C9-C11-C12	57.4(5)
C9-C11-C12-C13	146.3(4)	C36-C11-C12-C13	17.8(5)
C11-C12-C13-C14	11.3(5)	C12-C13-C14-C15	−166.1(4)
C12-C13-C14-C36	−36.8(4)	C13-C14-C15-C16	−61.6(5)
C36-C14-C15-C16	175.0(3)	C13-C14-C15-C33	177.3(4)
C36-C14-C15-C33	53.9(5)	C14-C15-C16-C17	−163.1(4)
C33-C15-C16-C17	−41.6(5)	C15-C16-C17-C18	9.9(6)
C16-C17-C18-C19	−175.2(4)	C16-C17-C18-C31	2.5(7)
C17-C18-C19-C20	−132.9(4)	C31-C18-C19-C20	49.3(5)
C18-C19-C20-O21	−173.3(4)	C18-C19-C20-C25	−56.4(5)
C19-C20-O21-C22	−89.3(5)	C25-C20-O21-C22	151.4(4)
C20-O21-C22-O23	−1.3(7)	C20-O21-C22-C24	178.9(4)

O21-C20-C25-C26	179.8(3)	C19-C20-C25-C26	60.3(5)
C20-C25-C26-O27	65.5(4)	C20-C25-C26-C31	−55.9(5)
C25-C26-O27-C28	84.2(4)	C31-C26-O27-C28	−151.1(4)
C26-O27-C28-O29	1.5(6)	C26-O27-C28-C30	−178.9(4)
C17-C18-C31-C32	−104.2(5)	C19-C18-C31-C32	73.5(5)
C17-C18-C31-C26	139.8(4)	C19-C18-C31-C26	−42.5(5)
C17-C18-C31-C33	17.9(6)	C19-C18-C31-C33	−164.3(4)
O27-C26-C31-C18	−75.4(4)	C25-C26-C31-C18	45.3(5)
O27-C26-C31-C32	167.1(3)	C25-C26-C31-C32	−72.2(4)
O27-C26-C31-C33	45.7(4)	C25-C26-C31-C33	166.4(4)
C14-C15-C33-C34	−46.1(5)	C16-C15-C33-C34	−168.4(4)
C14-C15-C33-C31	−174.3(4)	C16-C15-C33-C31	63.4(4)
C18-C31-C33-C34	−178.7(3)	C32-C31-C33-C34	−58.4(5)
C26-C31-C33-C34	59.8(5)	C18-C31-C33-C15	−50.2(5)
C32-C31-C33-C15	70.1(4)	C26-C31-C33-C15	−171.7(3)
C15-C33-C34-C35	48.6(5)	C31-C33-C34-C35	175.7(4)
C33-C34-C35-C36	−55.3(5)	C34-C35-C36-C37	−64.3(5)
C34-C35-C36-C14	58.0(5)	C34-C35-C36-C11	168.8(4)
C15-C14-C36-C35	−60.0(4)	C13-C14-C36-C35	169.6(3)
C15-C14-C36-C37	61.0(5)	C13-C14-C36-C37	−69.4(4)
C15-C14-C36-C11	178.1(3)	C13-C14-C36-C11	47.7(4)
C9-C11-C36-C35	80.7(5)	C12-C11-C36-C35	−154.2(4)
C9-C11-C36-C37	−46.1(5)	C12-C11-C36-C37	79.0(4)
C9-C11-C36-C14	−164.6(4)	C12-C11-C36-C14	−39.5(4)

Supplementary Table S7. Anisotropic atomic displacement parameters* (Å^2).

Atom	U_{11}	U_{22}	U_{33}	U_{23}	U_{13}	U_{12}
Si1	0.0287(6)	0.0217(7)	0.0220(6)	−0.0001(5)	0.0111(5)	0.0010(5)
C2	0.032(3)	0.047(3)	0.026(3)	−0.009(2)	0.008(2)	−0.007(2)
C3	0.054(4)	0.060(4)	0.030(3)	−0.018(3)	0.007(3)	−0.013(3)
C4	0.058(4)	0.072(5)	0.041(4)	−0.021(3)	0.022(3)	−0.041(4)
C5	0.042(3)	0.083(5)	0.038(4)	−0.008(3)	−0.006(3)	0.018(3)
C6	0.058(4)	0.033(3)	0.040(3)	0.003(3)	0.018(3)	0.015(3)
C7	0.046(3)	0.034(3)	0.037(3)	0.000(2)	0.021(3)	−0.008(3)
O8	0.0222(16)	0.0293(19)	0.0241(18)	−0.0046(14)	0.0060(13)	0.0021(14)
C9	0.022(2)	0.026(3)	0.021(2)	−0.0002(19)	0.0069(19)	0.0011(19)
C10	0.020(2)	0.040(3)	0.033(3)	−0.006(2)	0.006(2)	0.001(2)
C11	0.018(2)	0.020(2)	0.020(2)	0.0002(18)	0.0044(18)	−0.0009(18)
C12	0.019(2)	0.029(3)	0.024(3)	0.0016(19)	0.0058(19)	−0.0024(19)
C13	0.019(2)	0.027(3)	0.020(2)	−0.0038(19)	0.0016(18)	−0.0026(18)
C14	0.018(2)	0.018(3)	0.017(2)	−0.0004(17)	0.0023(17)	−0.0006(17)
C15	0.0144(19)	0.017(2)	0.016(2)	0.0010(16)	0.0042(16)	−0.0001(16)
C16	0.017(2)	0.019(2)	0.020(2)	−0.0021(18)	−0.0006(18)	0.0004(17)
C17	0.026(2)	0.018(2)	0.012(2)	−0.0023(17)	0.0047(18)	0.0007(18)

Atom	U_{11}	U_{22}	U_{33}	U_{23}	U_{13}	U_{12}
C18	0.020(2)	0.018(2)	0.017(2)	0.0019(18)	0.0046(18)	0.0025(17)
C19	0.022(2)	0.025(3)	0.021(2)	−0.0012(19)	0.0074(19)	0.0004(18)
C20	0.016(2)	0.022(3)	0.025(2)	−0.0025(19)	0.0089(18)	0.0001(18)
O21	0.0179(15)	0.0220(17)	0.0316(18)	−0.0028(14)	0.0122(13)	0.0023(13)
C22	0.023(2)	0.023(3)	0.022(2)	−0.0002(18)	0.0060(19)	0.0055(19)
O23	0.0283(17)	0.0223(19)	0.047(2)	−0.0067(16)	0.0182(16)	−0.0044(14)
C24	0.023(2)	0.020(3)	0.035(3)	0.003(2)	0.011(2)	0.0035(18)
C25	0.016(2)	0.021(3)	0.024(2)	−0.0009(18)	0.0019(18)	−0.0014(17)
C26	0.018(2)	0.019(2)	0.016(2)	−0.0012(18)	0.0037(17)	−0.0014(17)
O27	0.0187(15)	0.0227(18)	0.0177(16)	−0.0013(13)	0.0026(12)	−0.0018(12)
C28	0.023(2)	0.033(3)	0.019(2)	0.000(2)	0.0042(19)	0.001(2)
O29	0.044(2)	0.041(2)	0.0255(19)	0.0004(16)	−0.0090(16)	−0.0065(18)
C30	0.044(3)	0.041(3)	0.023(3)	0.010(2)	−0.005(2)	−0.003(2)
C31	0.017(2)	0.017(3)	0.017(2)	0.0004(17)	0.0056(17)	−0.0005(17)
C32	0.022(2)	0.020(2)	0.023(2)	0.0006(19)	0.0081(19)	−0.0011(18)
C33	0.0162(19)	0.018(2)	0.015(2)	0.0003(18)	0.0036(16)	0.0017(17)
C34	0.014(2)	0.028(3)	0.020(2)	−0.0042(19)	0.0004(18)	0.0006(18)
C35	0.019(2)	0.027(3)	0.016(2)	−0.0008(18)	0.0023(18)	0.0006(18)
C36	0.015(2)	0.019(2)	0.016(2)	0.0013(18)	0.0045(17)	0.0013(16)
C37	0.025(2)	0.017(3)	0.025(2)	0.0004(18)	0.007(2)	0.0016(18)

* The anisotropic atomic displacement factor exponent takes the form: $-2\pi^2$ [$h^2 a^{*2} U_{11}$ + ... + 2 h k $a^* b^* U_{12}$]

Supplementary Table S8. Hydrogen atomic coordinates and isotropic atomic displacement parameters ($Å^2$).

Atom	*x/a*	*y/b*	*z/c*	*U(eq)*
H3A	−0.2470	1.4949	1.0048	0.071
H3B	−0.2492	1.2927	1.0516	0.071
H3C	−0.1353	1.4002	1.0404	0.071
H4A	−0.1723	1.4742	0.8900	0.084
H4B	−0.0613	1.3817	0.9273	0.084
H4C	−0.1255	1.2599	0.8645	0.084
H5A	−0.1804	0.9568	1.0103	0.083
H5B	−0.1305	0.9442	0.9380	0.083
H5C	−0.0662	1.0660	1.0008	0.083
H6A	−0.4226	1.4966	0.9199	0.064
H6B	−0.3639	1.4854	0.8506	0.064
H6C	−0.4795	1.3753	0.8546	0.064
H7A	−0.4330	1.1157	1.0102	0.057
H7B	−0.4905	0.9849	0.9472	0.057
H7C	−0.3803	0.9023	0.9887	0.057
H9A	−0.3815	1.1349	0.7572	0.027
H10A	−0.5423	0.9294	0.7476	0.046
H10B	−0.5001	0.8098	0.8168	0.046
H10C	−0.5309	1.0497	0.8194	0.046

Atom	*x/a*	*y/b*	*z/c*	*U(eq)*
H11A	−0.3292	0.7036	0.7639	0.023
H12A	−0.4465	0.6887	0.6690	0.028
H12B	−0.4361	0.9309	0.6527	0.028
H13A	−0.3314	0.6310	0.5885	0.026
H13B	−0.3020	0.8732	0.5845	0.026
H14A	−0.2117	0.5793	0.6837	0.021
H15A	−0.0895	0.8499	0.6055	0.019
H16A	−0.1293	0.4152	0.5957	0.023
H16B	−0.1440	0.5804	0.5347	0.023
H17A	0.0203	0.3999	0.5205	0.022
H19A	0.2424	0.6401	0.5408	0.027
H19B	0.2047	0.4054	0.5278	0.027
H20A	0.2768	0.3112	0.6396	0.025
H24A	0.5735	0.3772	0.5506	0.039
H24B	0.5873	0.1369	0.5686	0.039
H24C	0.5340	0.2067	0.4942	0.039
H24D	0.5564	0.1034	0.5250	0.039
H24E	0.5425	0.3436	0.5070	0.039
H24F	0.5959	0.2738	0.5814	0.039
H25A	0.3383	0.7392	0.6569	0.024
H25B	0.3695	0.5625	0.7129	0.024
H26A	0.2168	0.7570	0.7415	0.021
H30A	0.1426	0.1532	0.8014	0.055
H30B	0.2565	0.1448	0.8486	0.055
H30C	0.1531	0.2546	0.8764	0.055
H30D	0.2255	0.2152	0.8829	0.055
H30E	0.1116	0.2236	0.8357	0.055
H30F	0.2151	0.1138	0.8079	0.055
H32A	0.1333	1.0202	0.6708	0.031
H32B	0.2021	0.9343	0.6117	0.031
H32C	0.0736	0.9726	0.5960	0.031
H33A	−0.0053	0.5415	0.7020	0.019
H34A	0.0499	0.7842	0.7848	0.025
H34B	0.0096	0.9714	0.7352	0.025
H35A	−0.1320	0.6836	0.7949	0.024
H35B	−0.1193	0.9230	0.8148	0.024
H37A	−0.2571	1.1330	0.6607	0.033
H37B	−0.2011	1.1951	0.7353	0.033
H37C	−0.1274	1.1159	0.6776	0.033

4. Crystallographic analysis of VDR-1,20S$(OH)_2D_3$.

Supplementary Table S9: Crystallographic data collection and refinement statistics for zVDR LBD in complex with 1,20S$(OH)_2D_3$.

Data processing		
Beamline		ID23-1
X-ray source detector		PILATUS 6M
λ (Å)		1.000000
Resolution (Å)		47.85 - 1.98 (2.051 - 1.98)
Crystal space group		$P\ 6_522$
Cell parameters (Å)		*a* = *b* = 65.936;<br>*c* = 262.97
Unique reflections		24360 (2364)
Mean redundancy		2.0 (2.0)
Rsym (%)		1.54 (27.11)
Mean I/σ(I)		14.92 (2.32)
completeness (%)		98 (100)
Refinement		
rmsd bond length (Å)		0.003
rmsd bond angles (deg)		0.62
Rcryst (%)		20.01
Rfree (%)		22.50
no. of non-H atoms		
	protein	1992
	ligands	30
	water	58
average B factor		
	protein	71.72
	ligands	59.89
	water	71.70
Ramachandran plots		
	favored (%)	99
	allowed (%)	1.2

5. Spectra.

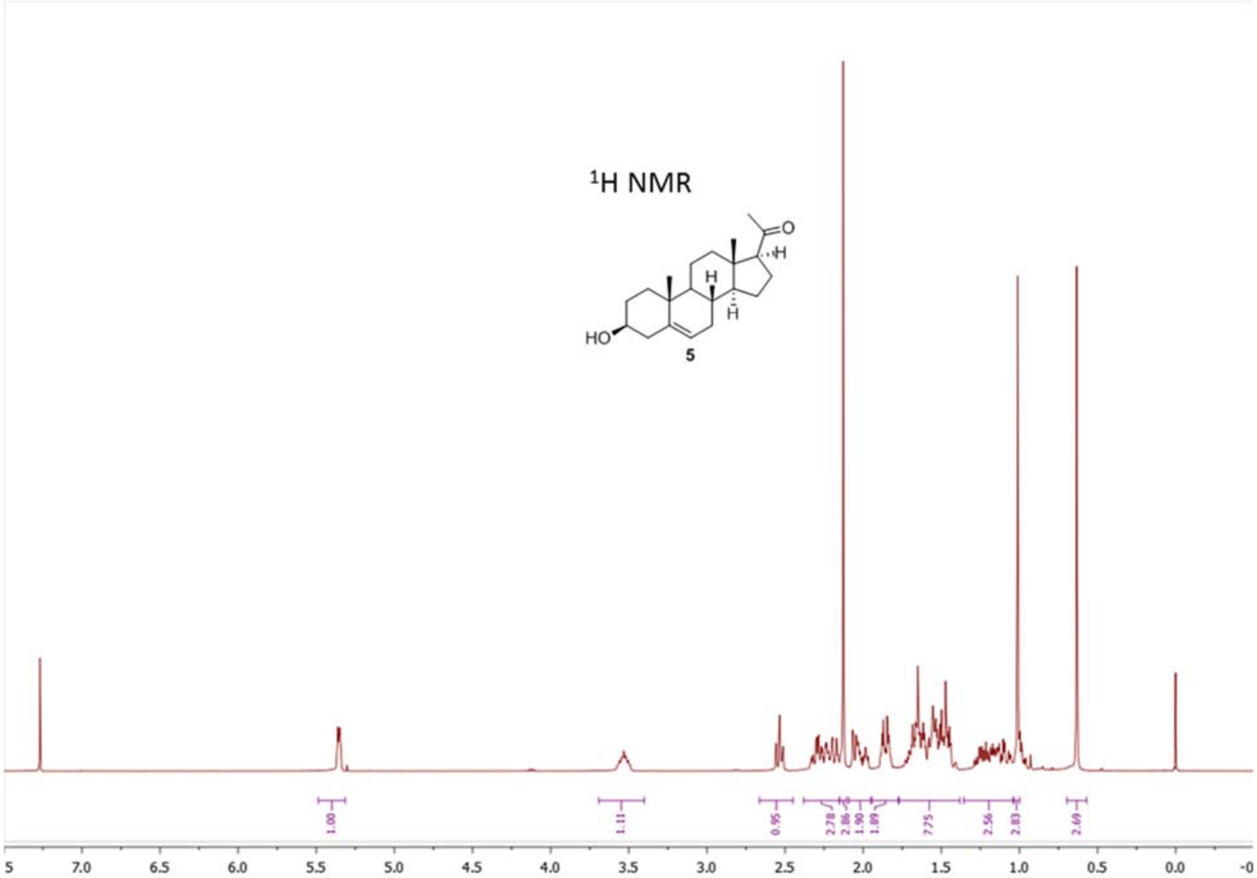
^{1}H NMR
HO
5
1.00
1.11
0.95
2.78
2.86
1.90
1.89
7.75
2.56
2.83
2.69
7.0
6.5
6.0
5.5
5.0
4.5
4.0
3.5
3.0
2.5
2.0
1.5
1.0
0.5
0.0

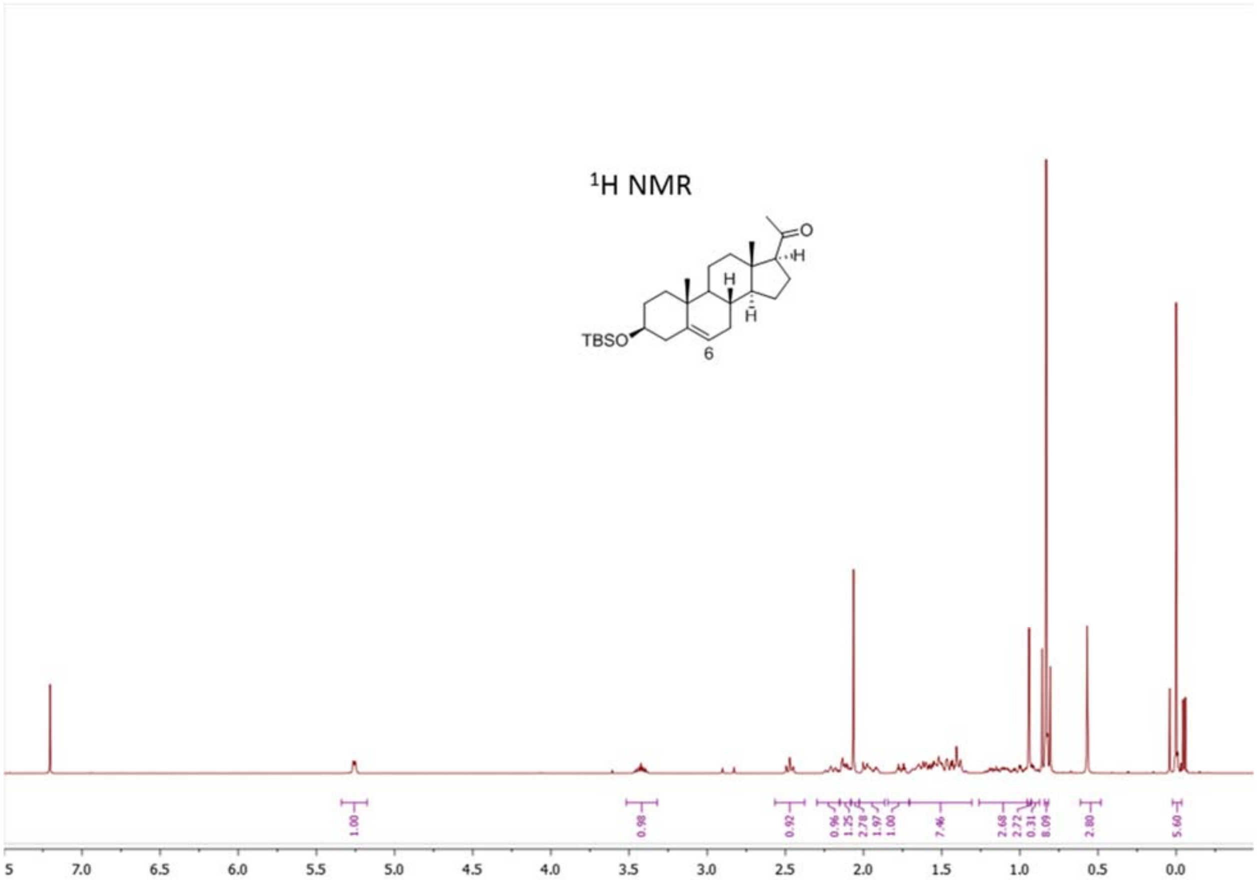

1H NMR
TBSO
6
1.00
0.98
0.92
0.96
1.25
2.78
1.97
1.00
7.46
2.68
2.72
0.31
8.09
2.80
5.60
7.0
6.5
6.0
5.5
5.0
4.5
4.0
3.5
3.0
2.5
2.0
1.5
1.0
0.5
0.0

365

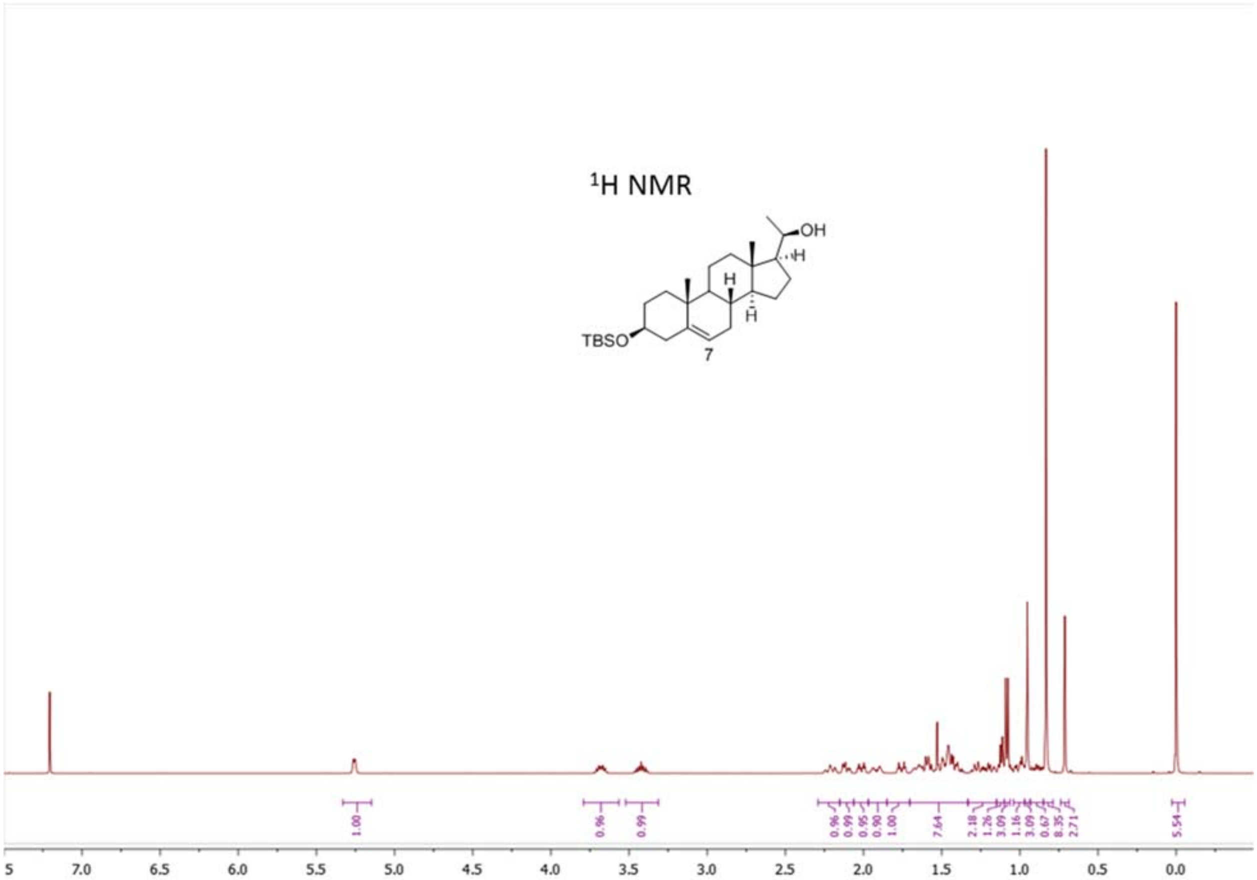

^{1}H NMR
OH
H
H
H
TBSO
7
1.00
0.96
0.99
0.96
0.99
0.95
0.90
1.00
7.64
2.18
1.26
3.09
1.16
3.09
0.67
8.35
2.71
5.54
7.0
6.5
6.0
5.5
5.0
4.5
4.0
3.5
3.0
2.5
2.0
1.5
1.0
0.5
0.0

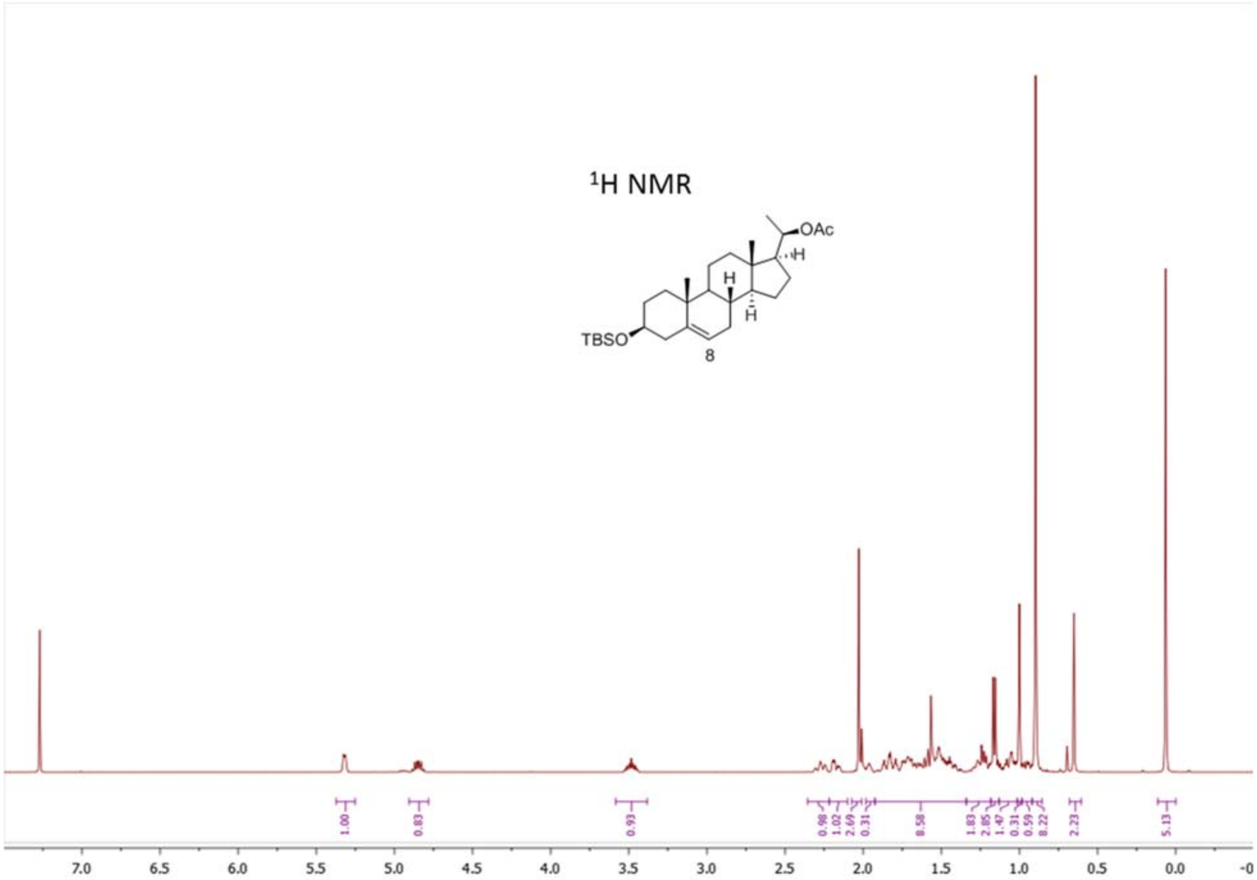

1H NMR
OAc
H
H
H
TBSO
8
1.00
0.83
0.93
0.98
1.02
2.69
0.31
8.58
1.83
2.85
1.47
0.31
0.59
8.22
2.23
5.13
7.0
6.5
6.0
5.5
5.0
4.5
4.0
3.5
3.0
2.5
2.0
1.5
1.0
0.5
0.0

367

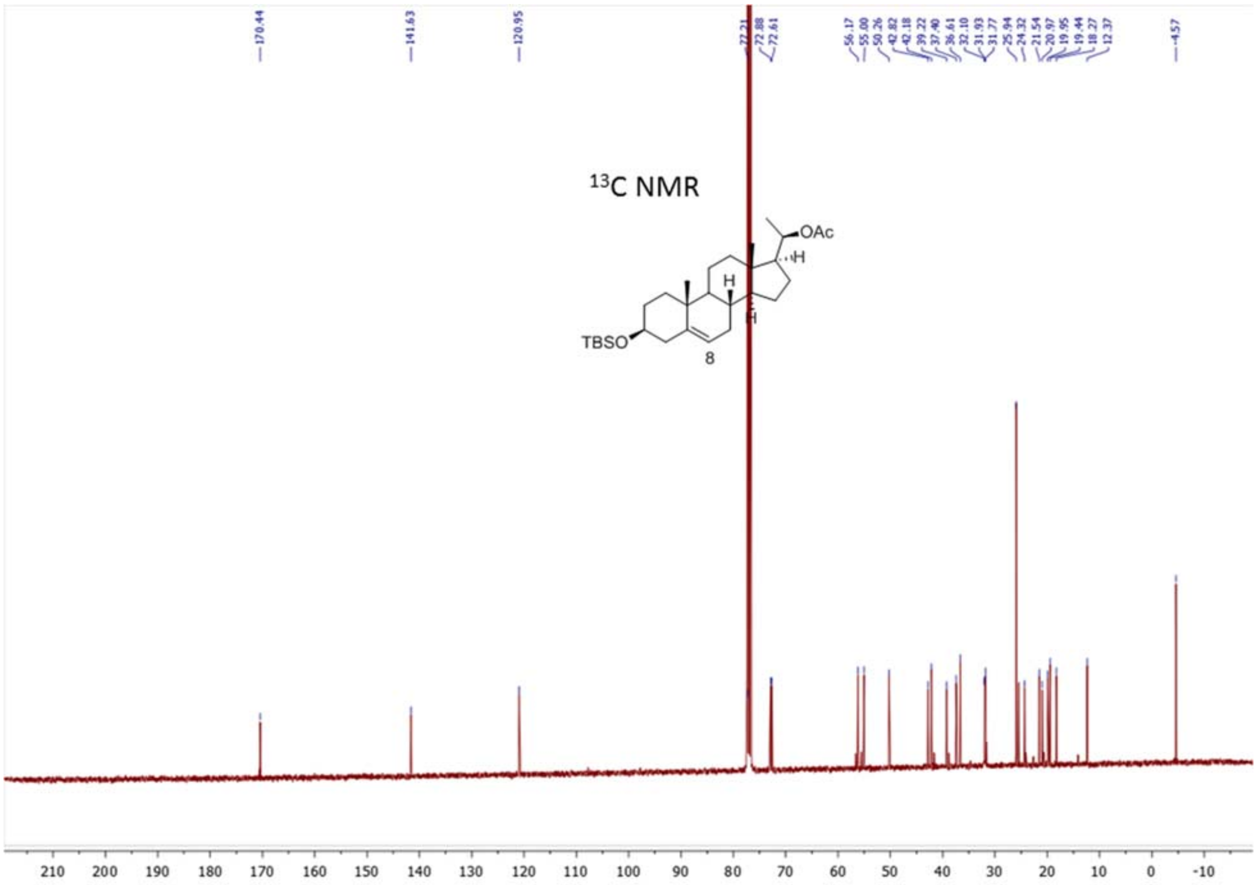

170.44
141.63
120.95
77.21
72.88
72.61
56.17
55.00
50.26
42.82
42.18
39.22
37.40
36.61
32.10
31.93
31.77
25.94
24.32
21.54
20.97
19.95
19.44
18.27
12.37
-4.57
^{13}C NMR
OAc
H
H
H
TBSO
8
210
200
190
180
170
160
150
140
130
120
110
100
90
80
70
60
50
40
30
20
10
0
-10

368

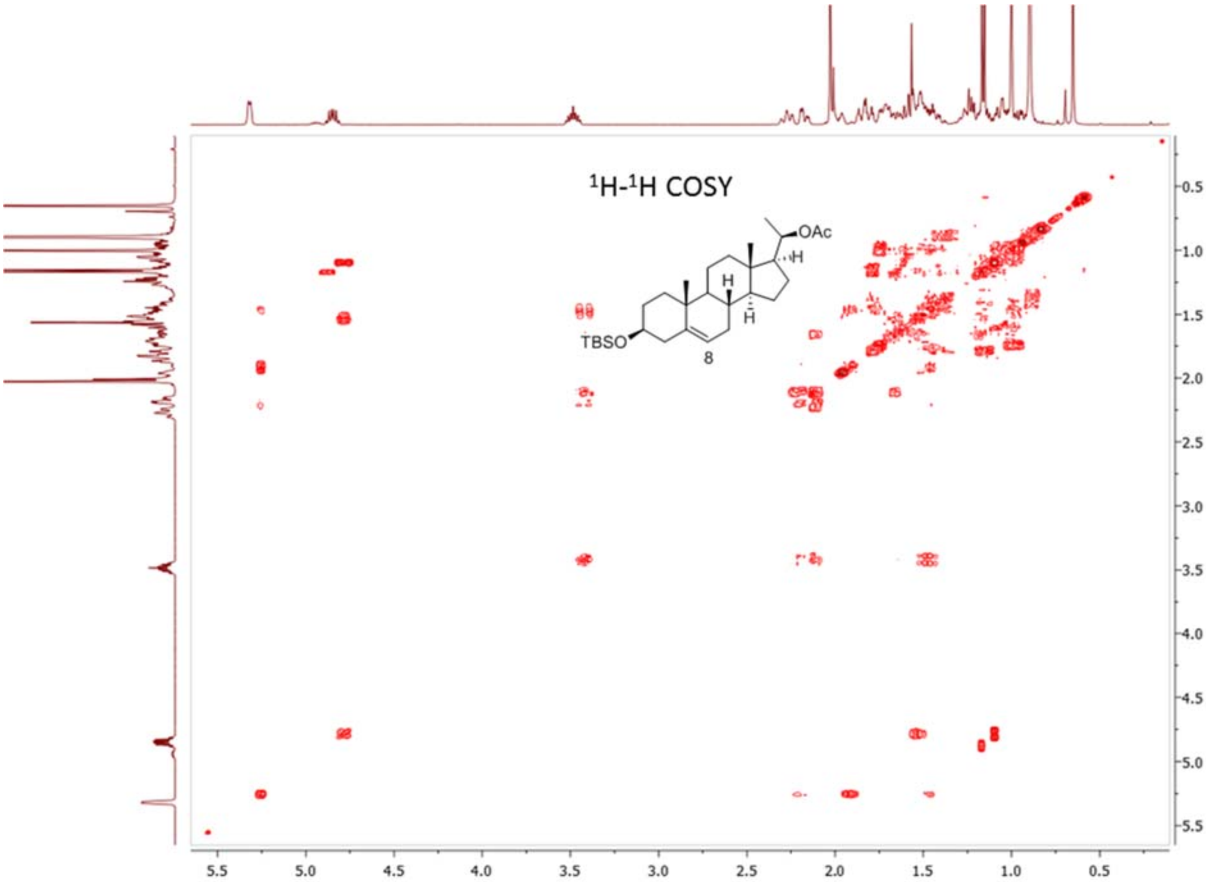

369

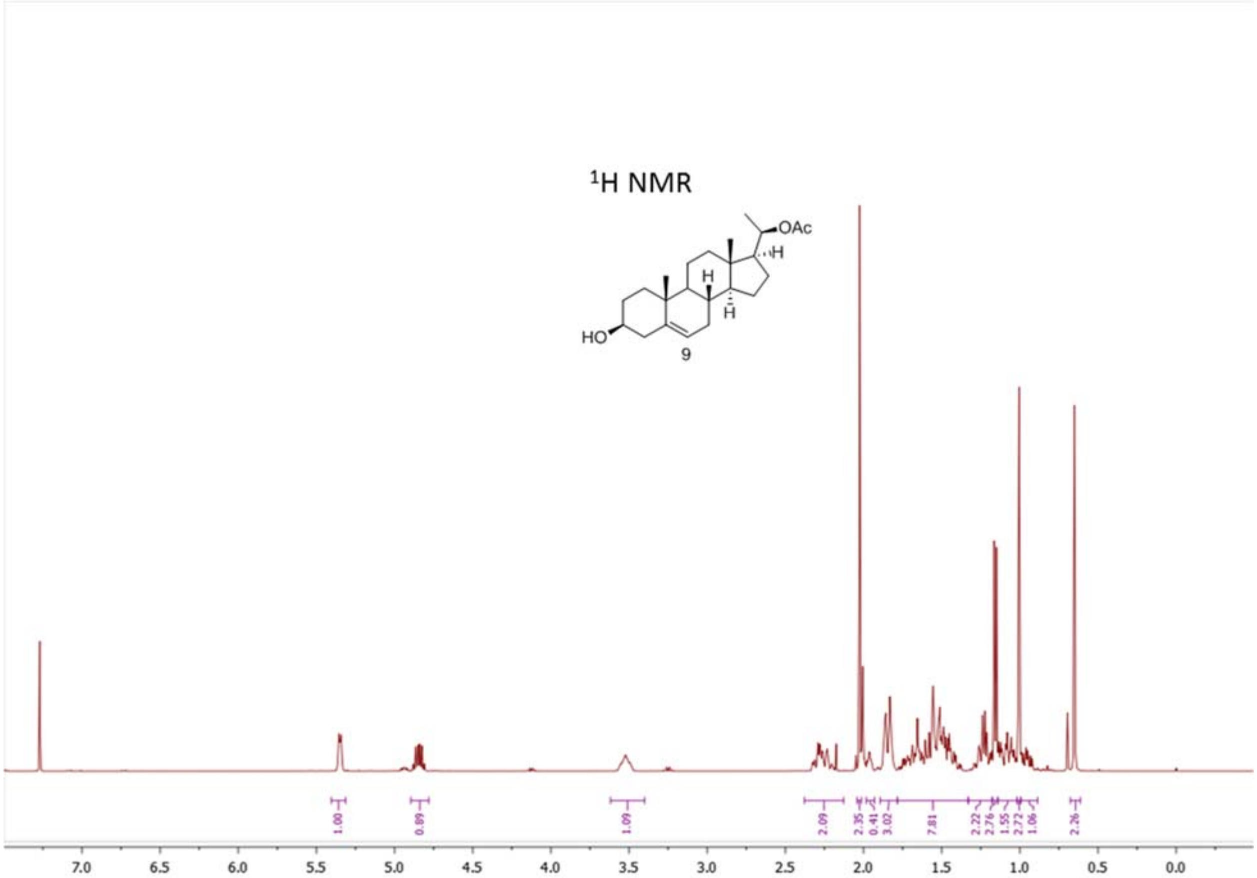

^{1}H NMR
OAc
H
HO
9
1.00
0.89
1.09
2.09
2.35
0.41
3.02
7.81
2.22
2.76
1.55
2.72
1.06
2.26
7.0
6.5
6.0
5.5
5.0
4.5
4.0
3.5
3.0
2.5
2.0
1.5
1.0
0.5
0.0

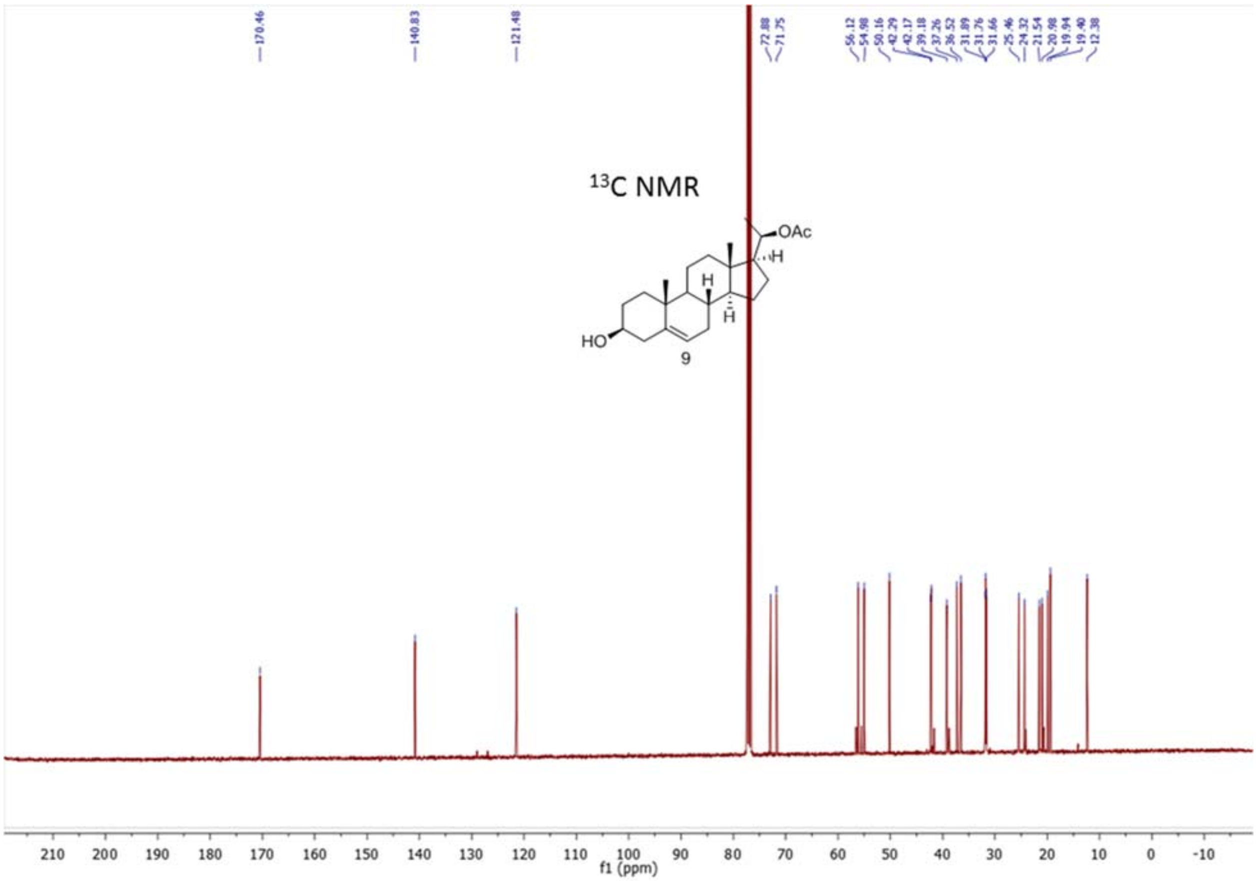

170.46
140.83
121.48
72.88
71.75
56.12
54.98
50.16
42.29
42.17
39.18
37.26
36.52
31.89
31.76
31.66
25.46
24.32
21.54
20.98
19.94
19.40
12.38
^{13}C NMR
OAc
H
H
H
HO
9
210 200 190 180 170 160 150 140 130 120 110 100 90 80 70 60 50 40 30 20 10 0 -10
f1 (ppm)

371

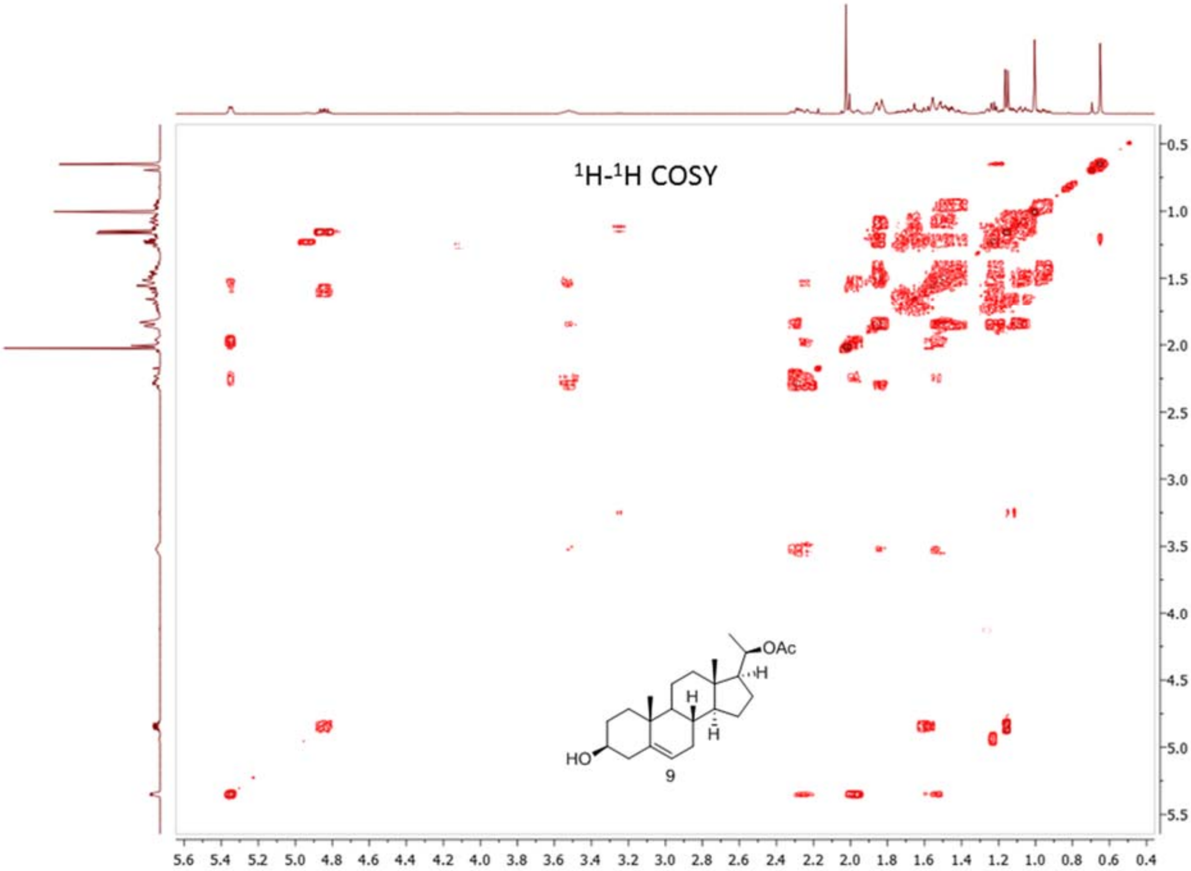

372

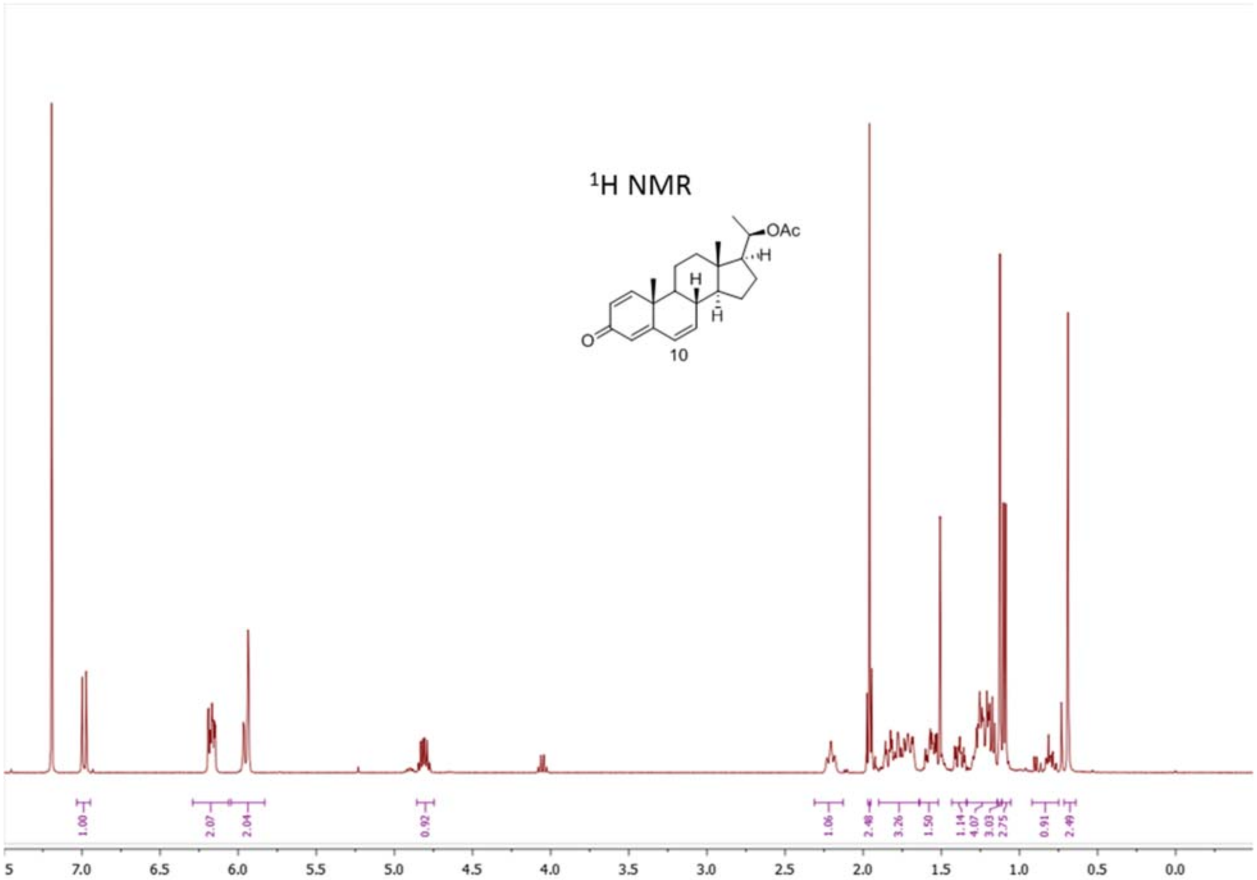

1H NMR
OAc
H
H
H
O
10
1.00
2.07
2.04
0.92
1.06
2.48
3.26
1.50
1.14
4.07
3.03
2.75
0.91
2.49
7.0
6.5
6.0
5.5
5.0
4.5
4.0
3.5
3.0
2.5
2.0
1.5
1.0
0.5
0.0

373

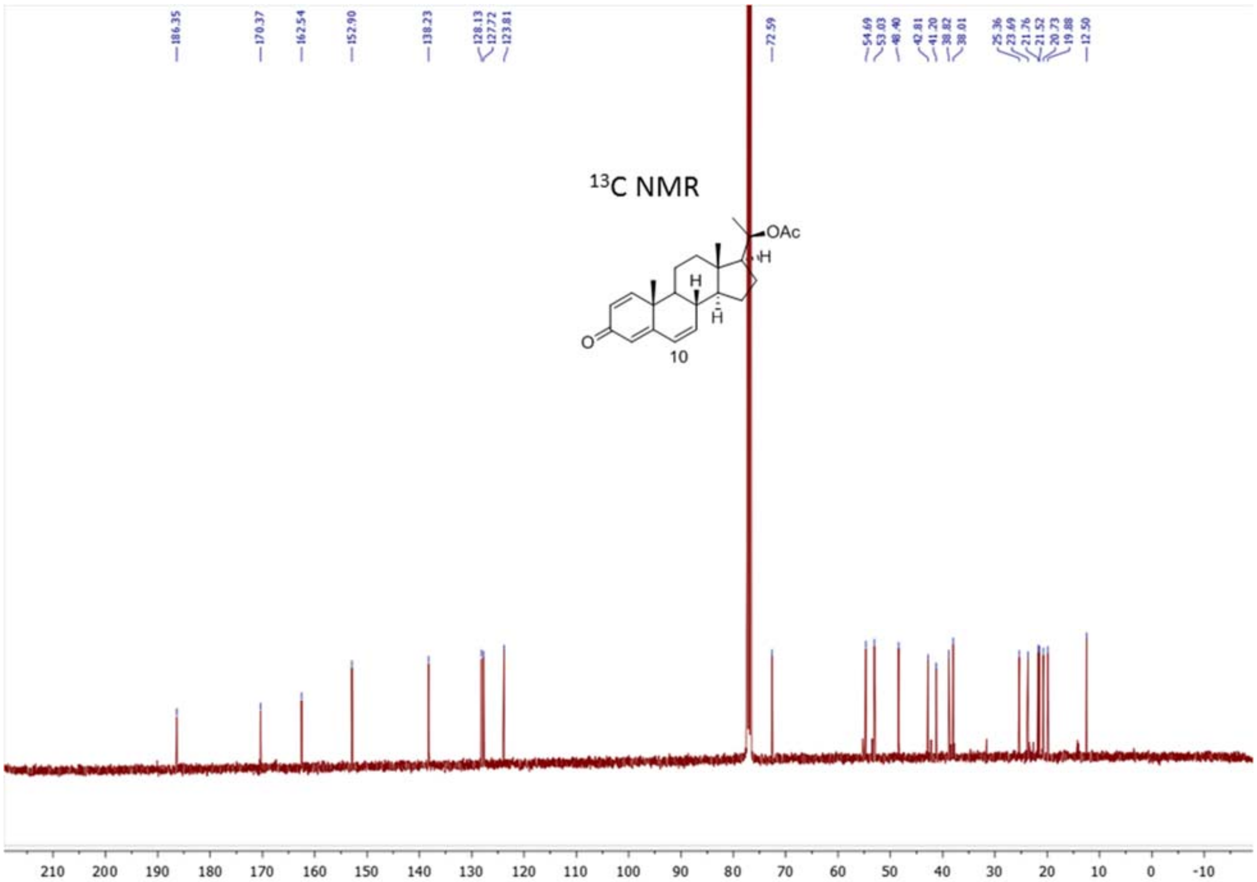
186.35
170.37
162.54
152.90
138.23
128.13
127.72
123.81
72.59
54.69
53.03
48.40
42.81
41.20
38.82
38.01
25.36
23.69
21.76
21.52
20.73
19.88
12.50
^{13}C NMR
OAc
H
10
210 200 190 180 170 160 150 140 130 120 110 100 90 80 70 60 50 40 30 20 10 0 -10

374

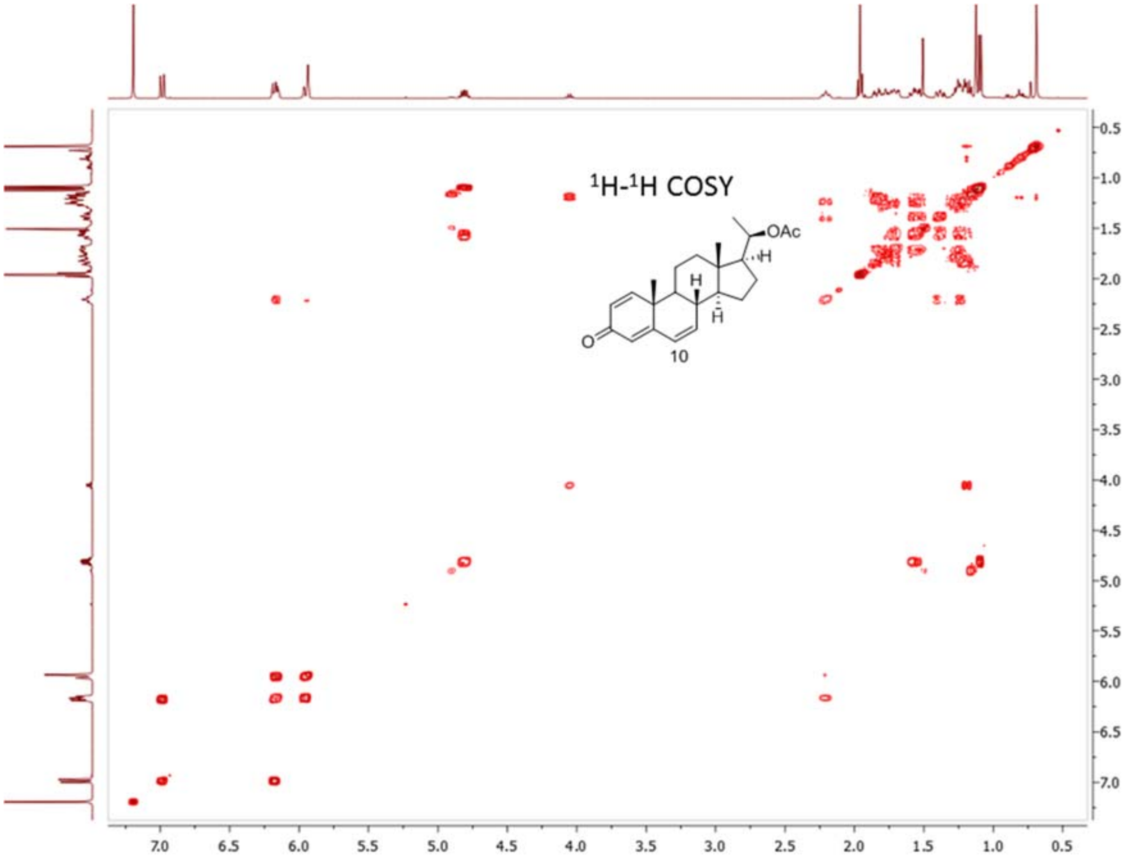

375

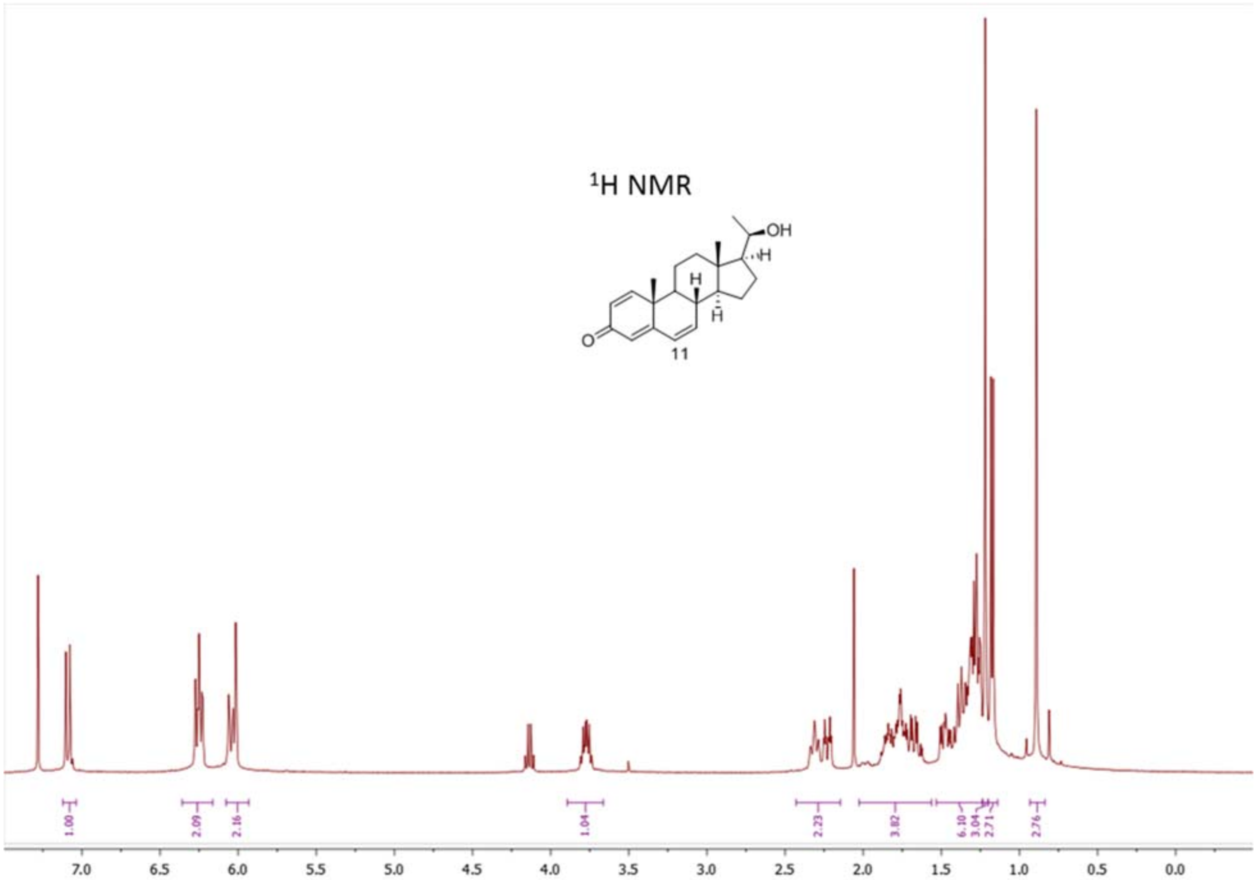

1H NMR
OH
H
H
H
O
11
1.00
2.09
2.16
1.04
2.23
3.82
6.10
3.04
2.71
2.76
7.0
6.5
6.0
5.5
5.0
4.5
4.0
3.5
3.0
2.5
2.0
1.5
1.0
0.5
0.0

376

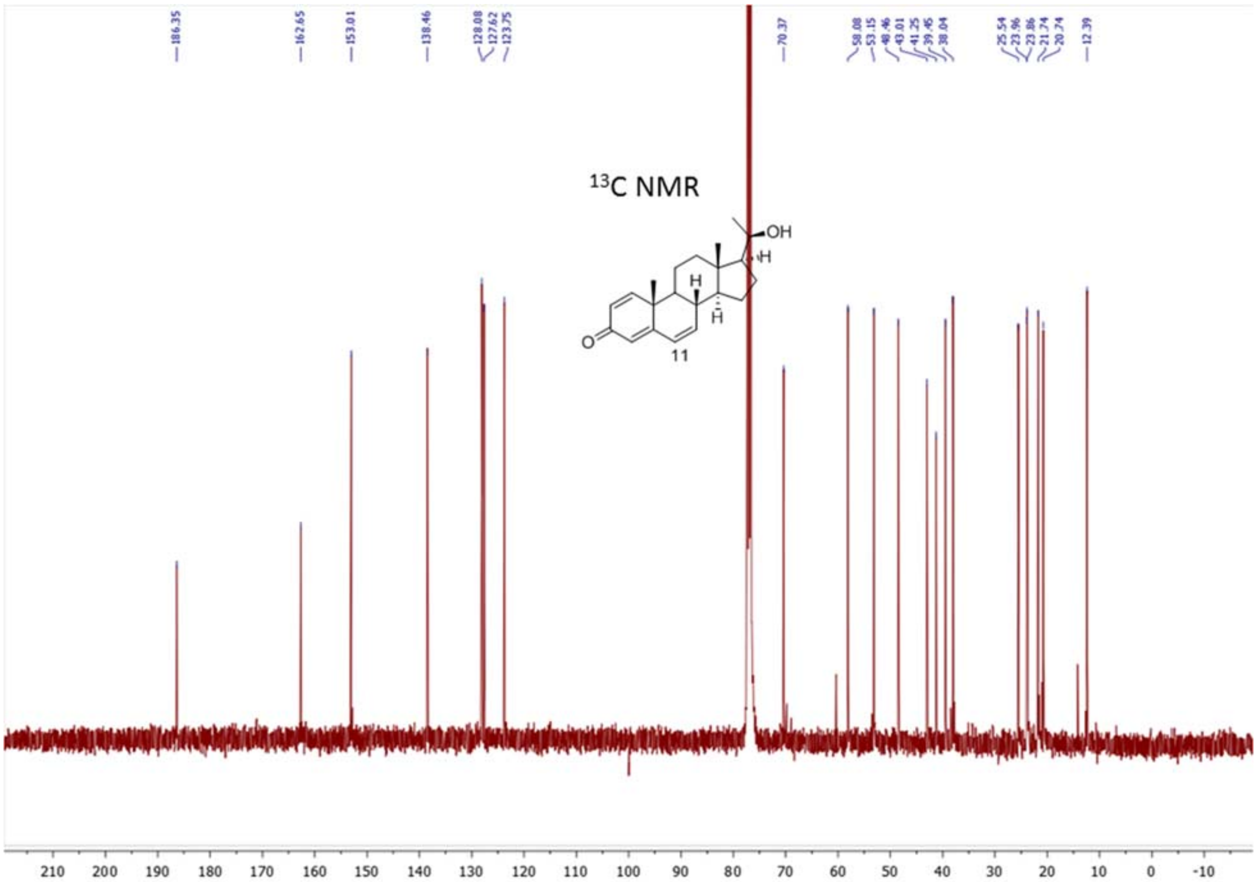
186.35
162.65
153.01
138.46
128.08
127.62
123.75
70.37
58.08
53.15
48.46
43.01
41.25
39.45
38.04
25.54
23.96
23.86
21.74
20.74
12.39
^{13}C NMR
OH
H
11
210 200 190 180 170 160 150 140 130 120 110 100 90 80 70 60 50 40 30 20 10 0 -10

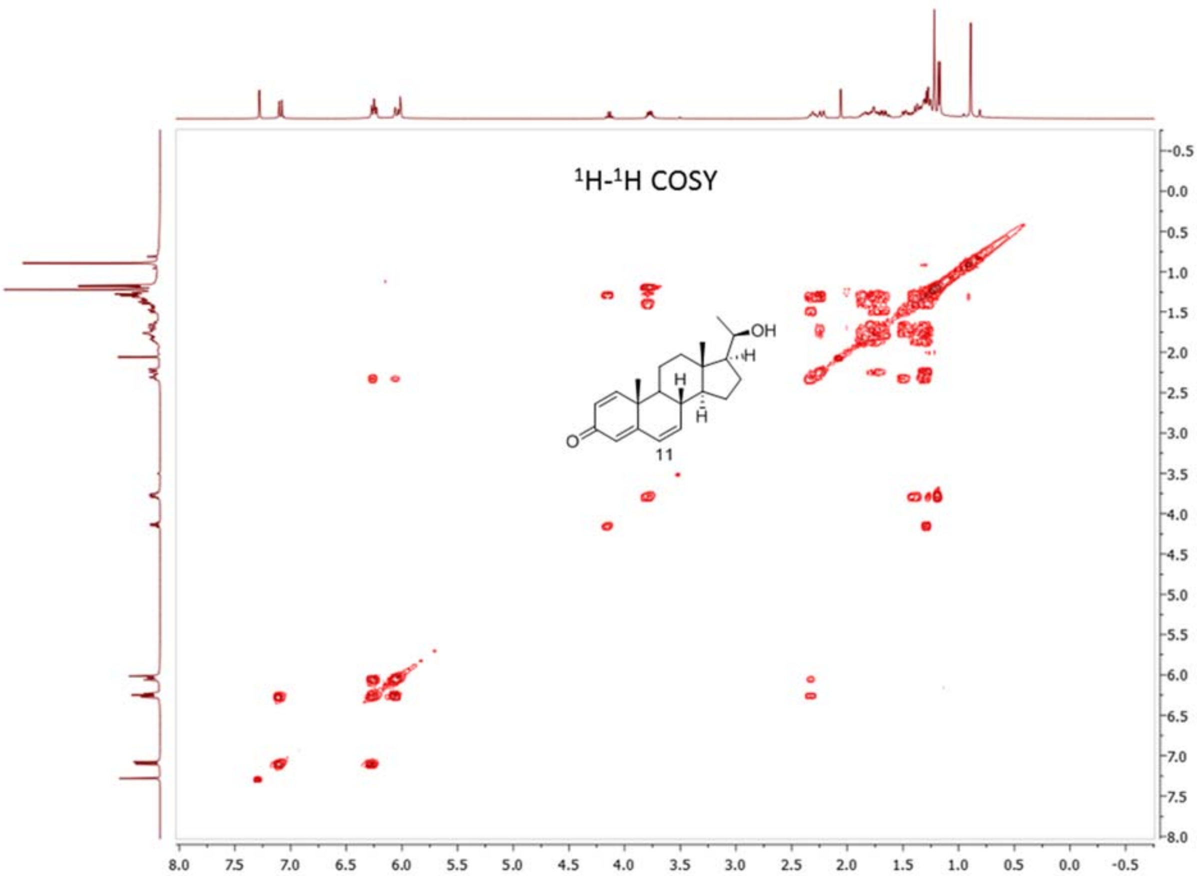

378

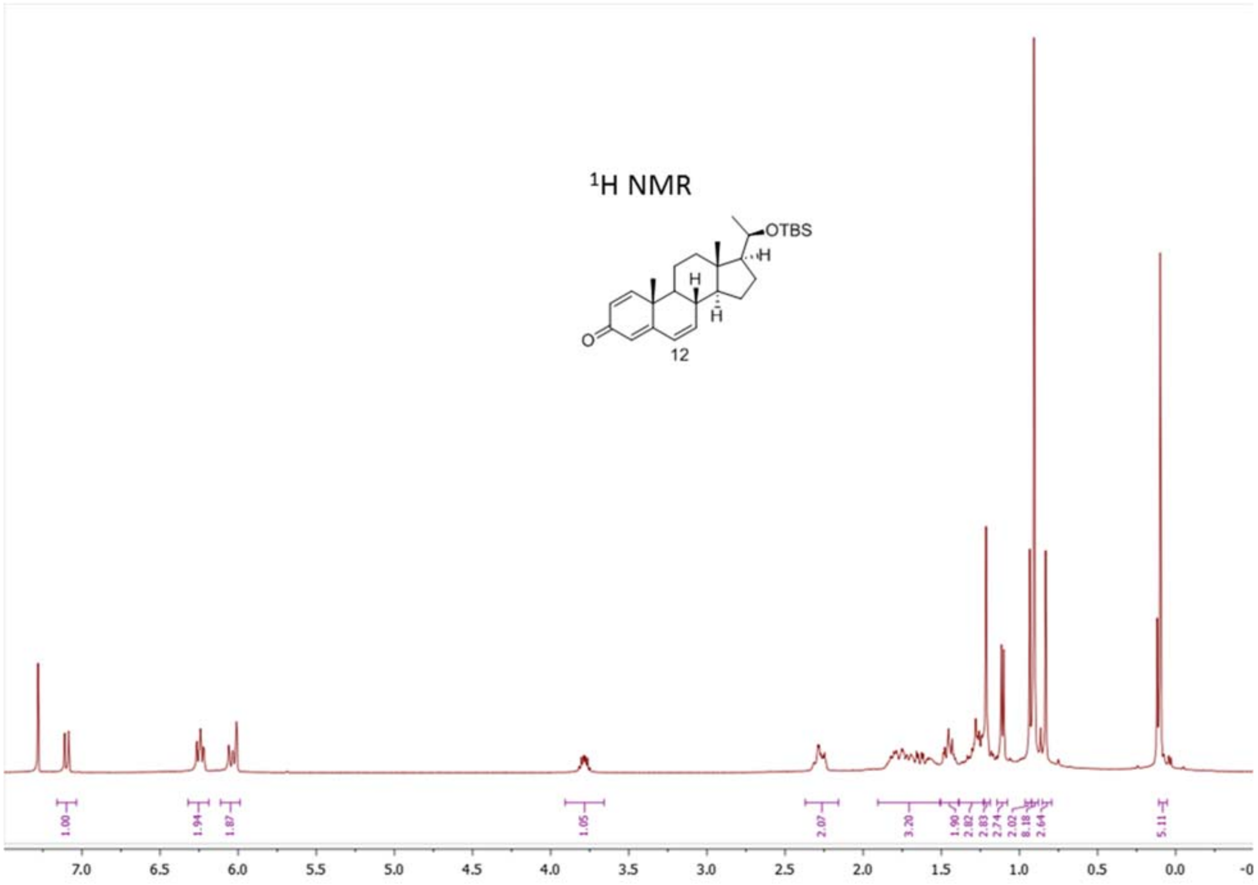

1H NMR
OTBS
H
H
H
O
12
1.00
1.94
1.87
1.05
2.07
3.20
1.90
2.82
2.83
2.74
2.02
8.18
2.64
5.11
7.0
6.5
6.0
5.5
5.0
4.5
4.0
3.5
3.0
2.5
2.0
1.5
1.0
0.5
0.0

379

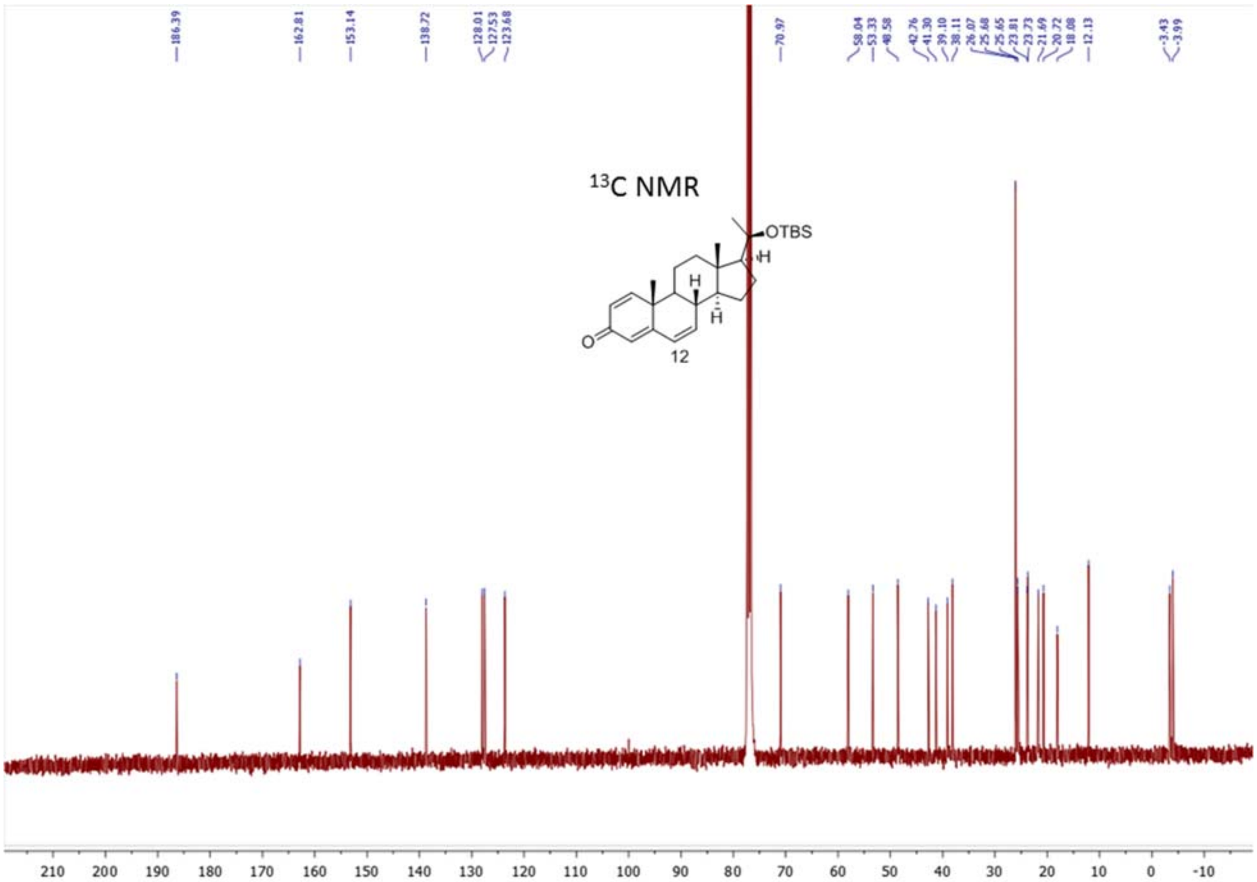
186.39
162.81
153.14
138.72
128.01
127.53
123.68
70.97
58.04
53.33
48.58
42.76
41.30
39.10
38.11
26.07
25.68
25.65
23.81
23.73
21.69
20.72
18.08
12.13
-3.43
-3.99
13C NMR
OTBS
H
H
H
O
12
210 200 190 180 170 160 150 140 130 120 110 100 90 80 70 60 50 40 30 20 10 0 -10

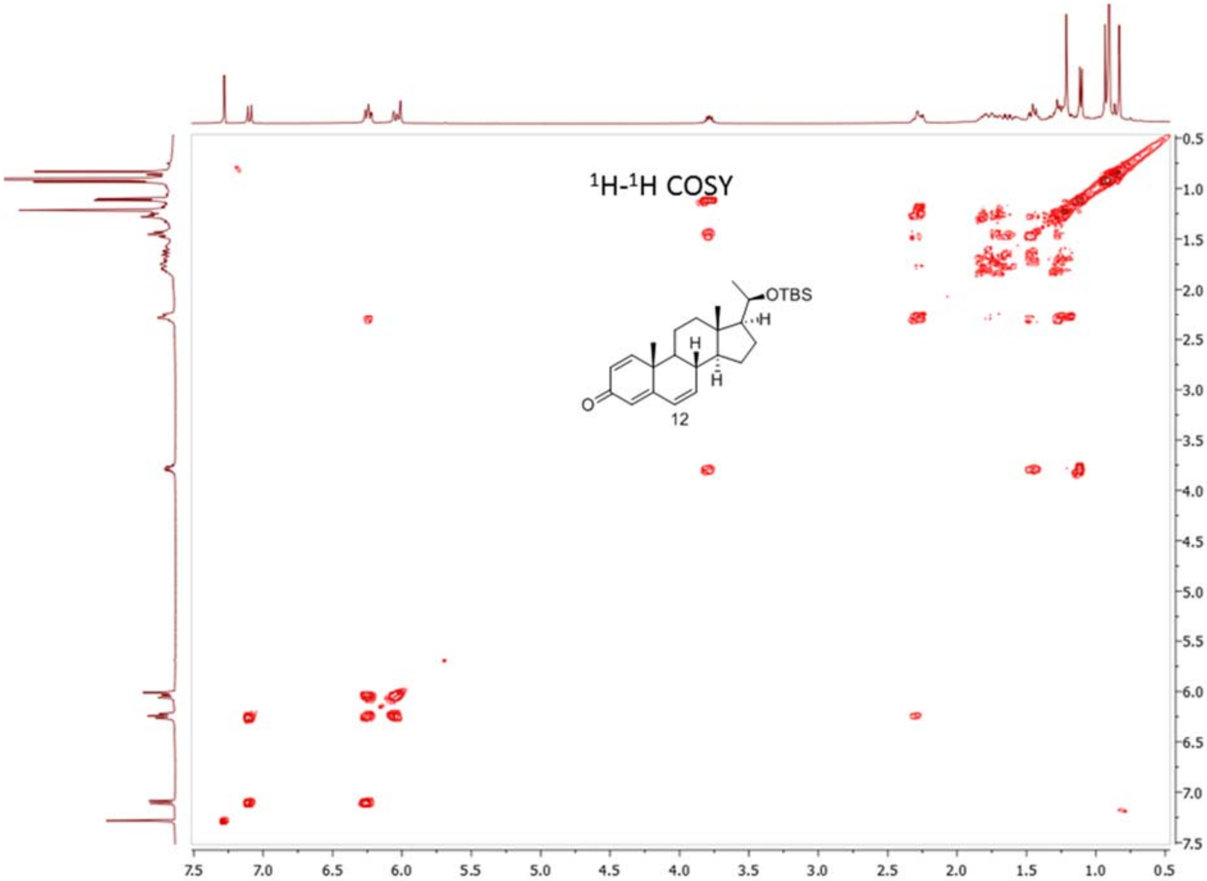
1H-1H COSY
OTBS
12
7.5
7.0
6.5
6.0
5.5
5.0
4.5
4.0
3.5
3.0
2.5
2.0
1.5
1.0
0.5

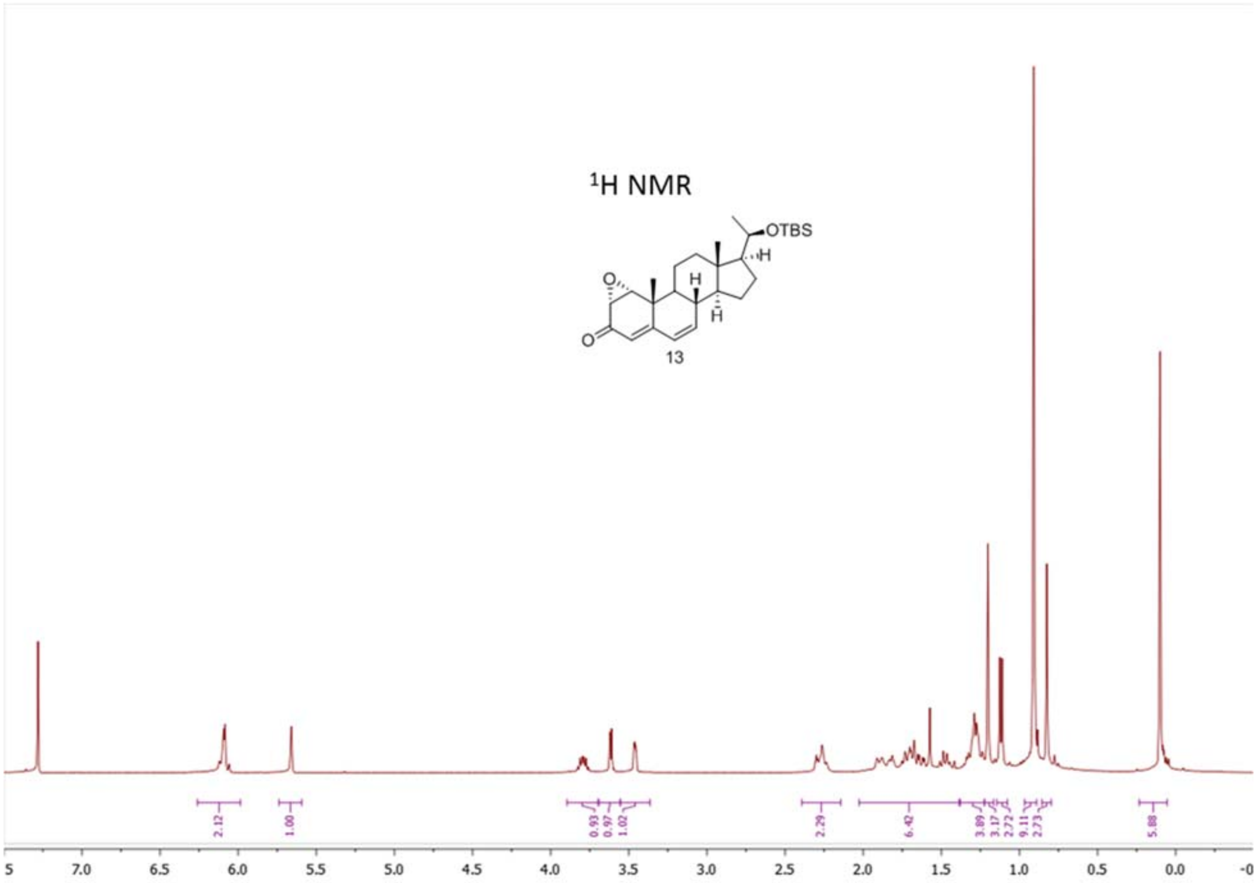

¹H NMR
OTBS
13
2.12
1.00
0.93
0.97
1.02
2.29
6.47
3.89
3.17
2.72
9.11
2.73
5.88
7.0
6.5
6.0
5.5
5.0
4.5
4.0
3.5
3.0
2.5
2.0
1.5
1.0
0.5
0.0

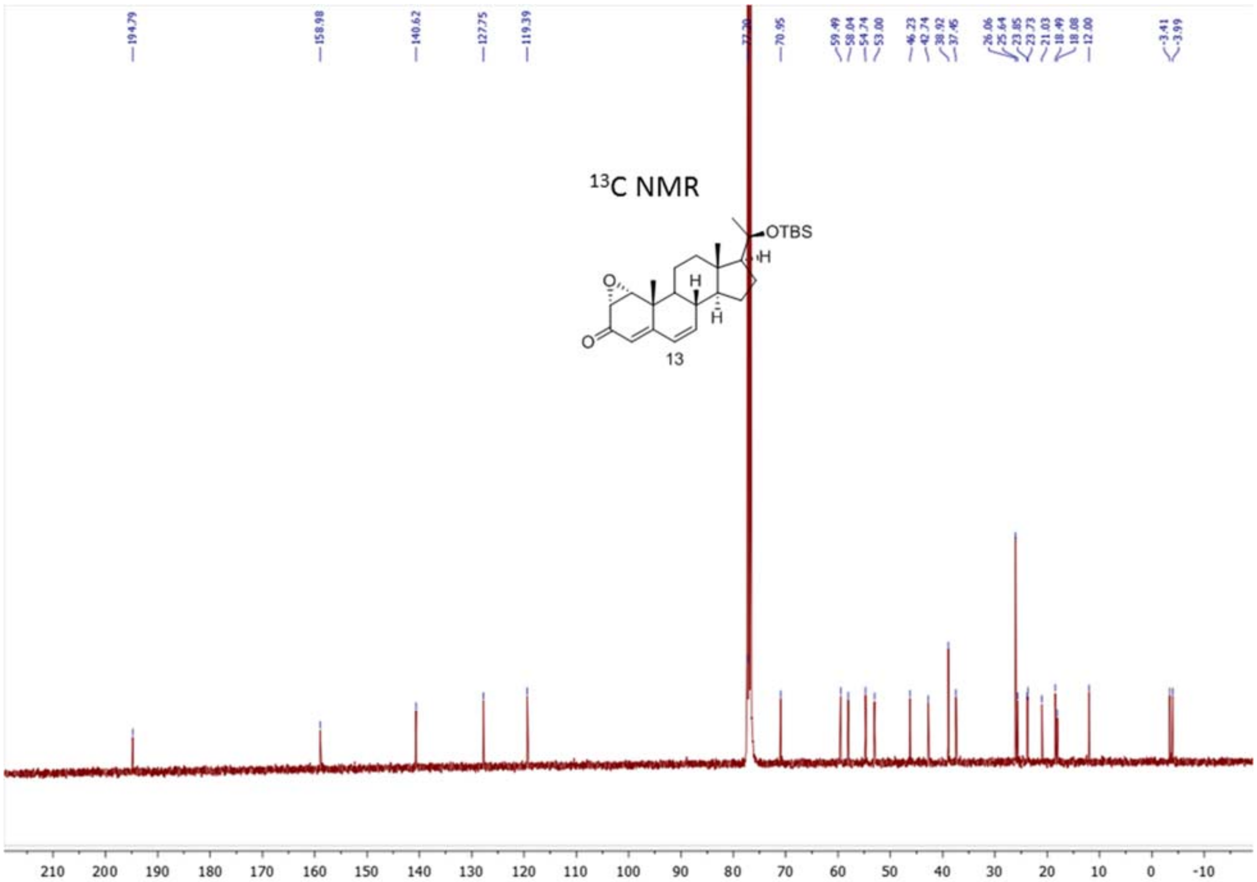

194.79
158.98
140.62
127.75
119.39
70.95
59.49
58.04
54.74
53.00
46.23
42.74
38.92
37.45
26.06
25.64
23.85
23.73
21.03
18.49
18.08
12.00
-3.41
-3.99
^{13}C NMR
OTBS
13
210 200 190 180 170 160 150 140 130 120 110 100 90 80 70 60 50 40 30 20 10 0 -10

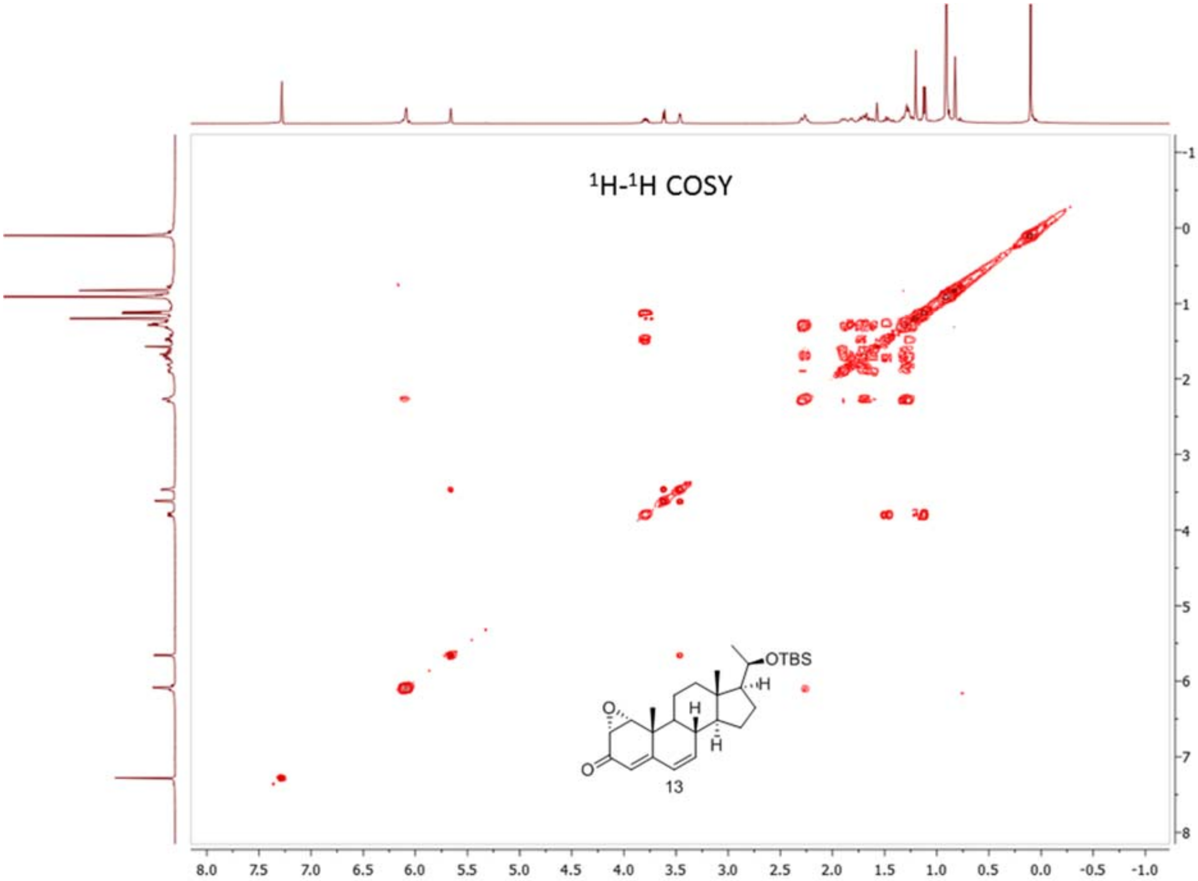

¹H-¹H COSY
OTBS
H
H
H
O
O
13
8.0
7.5
7.0
6.5
6.0
5.5
5.0
4.5
4.0
3.5
3.0
2.5
2.0
1.5
1.0
0.5
0.0
-0.5
-1.0
-1
0
1
2
3
4
5
6
7
8

385

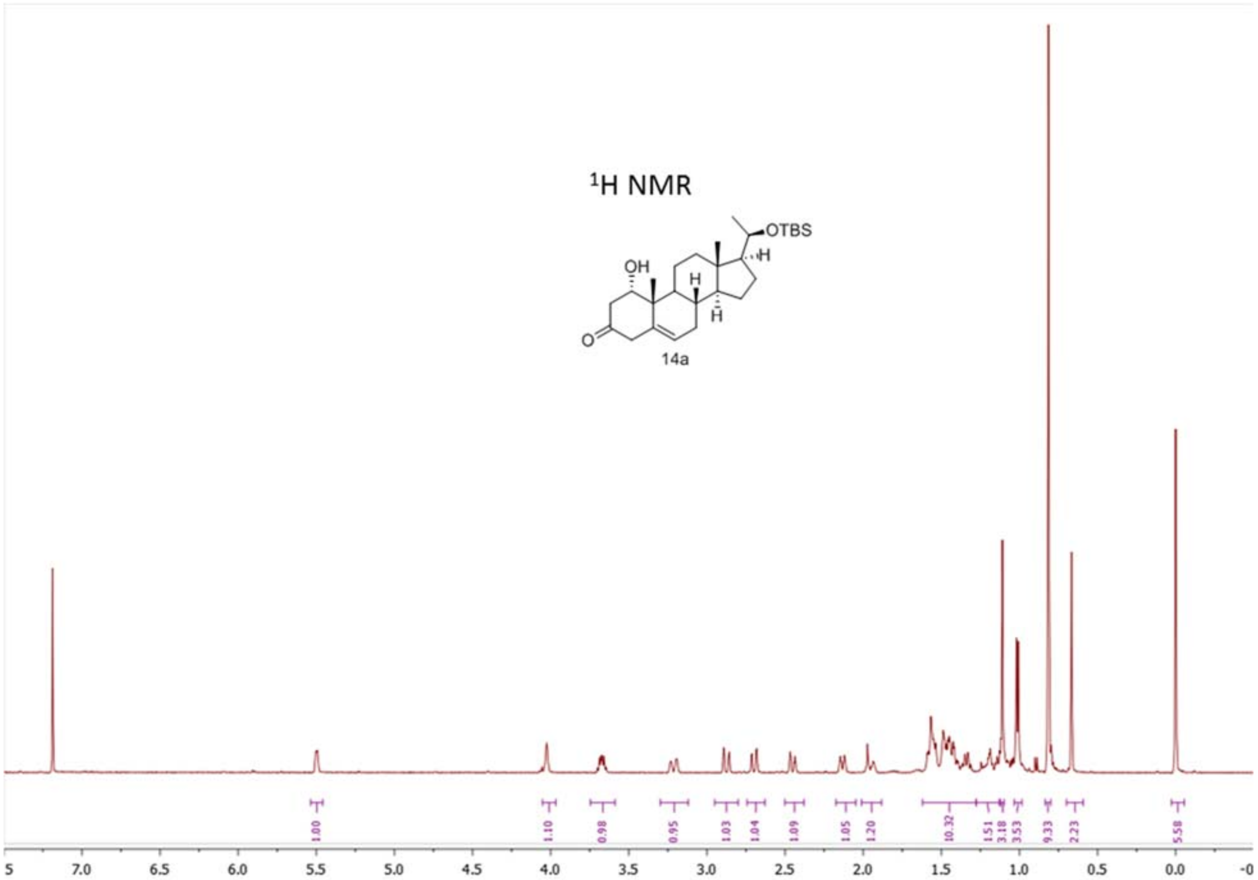
1H NMR
OTBS
OH
H
14a
1.00
1.10
0.98
0.95
1.03
1.04
1.09
1.05
1.20
10.32
1.51
3.18
3.53
9.33
2.23
5.58
7.0
6.5
6.0
5.5
5.0
4.5
4.0
3.5
3.0
2.5
2.0
1.5
1.0
0.5
0.0

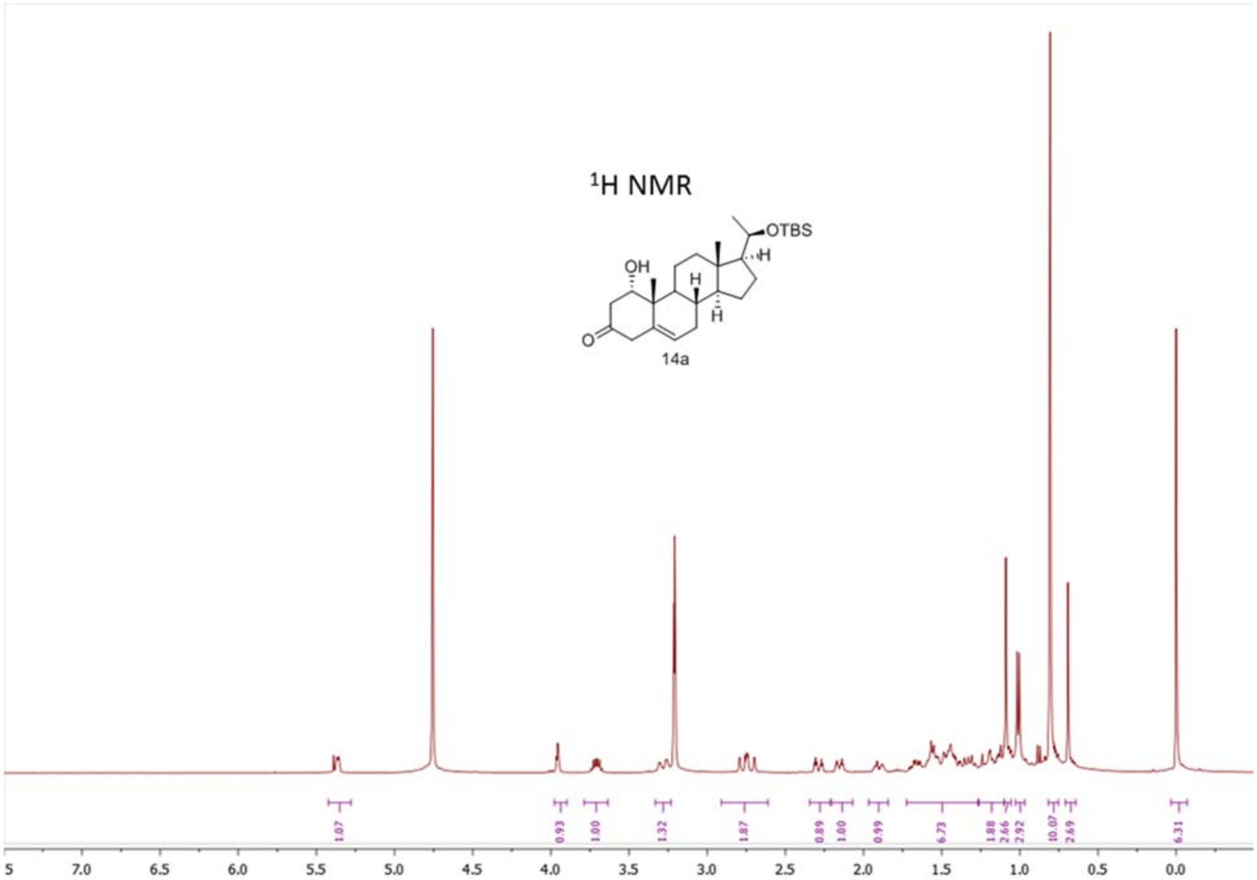

1H NMR
OH
OTBS
H
H
H
O
14a
1.07
0.93
1.00
1.32
1.87
0.89
1.00
0.99
6.73
1.88
2.66
2.92
10.07
2.69
6.31
7.0
6.5
6.0
5.5
5.0
4.5
4.0
3.5
3.0
2.5
2.0
1.5
1.0
0.5
0.0

387

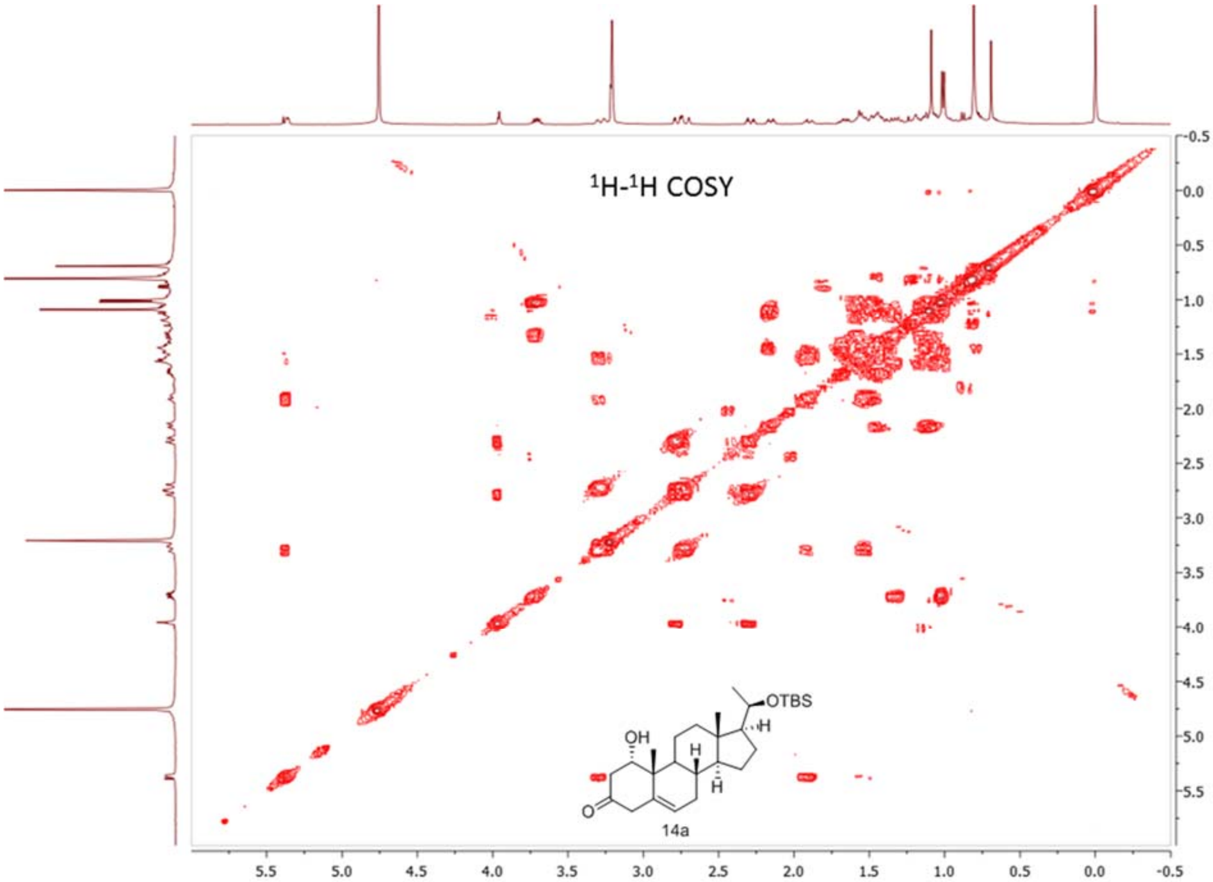

388

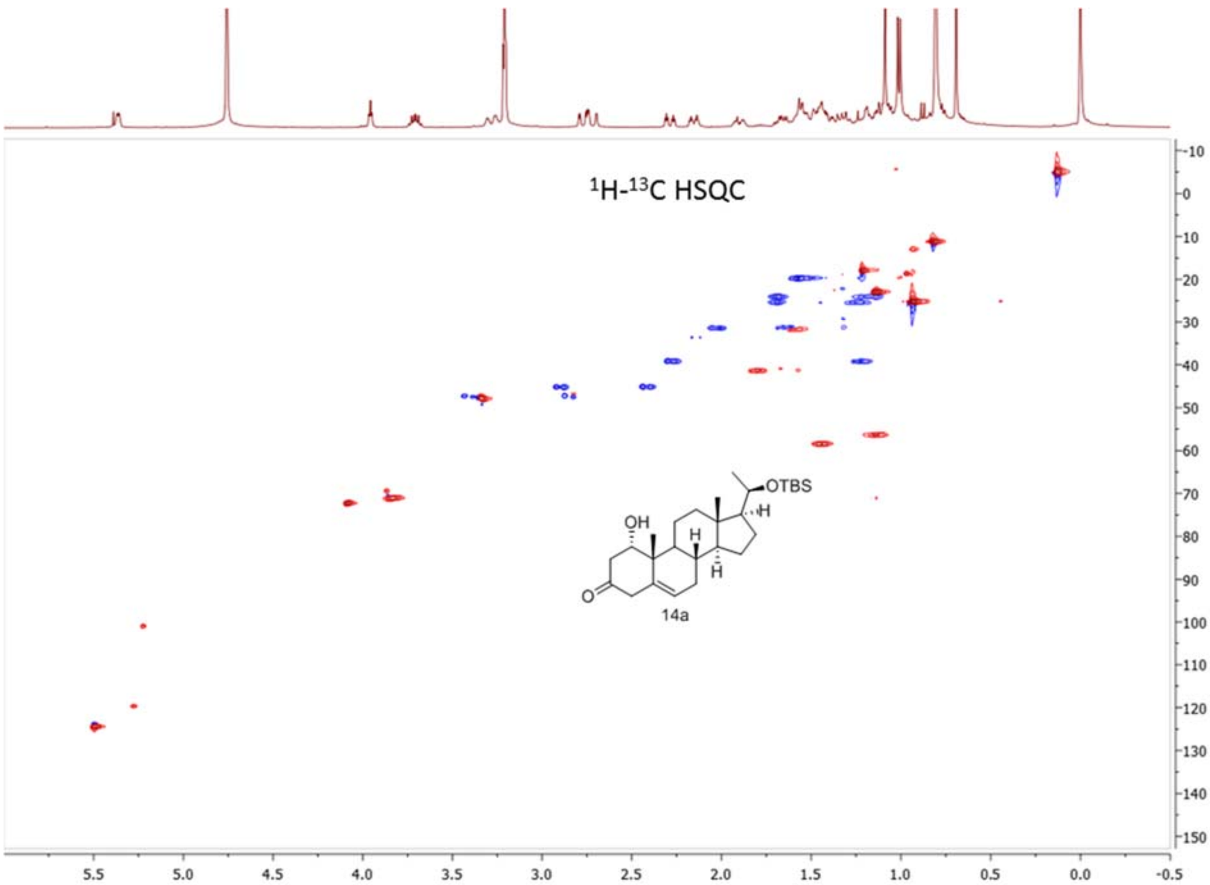

389

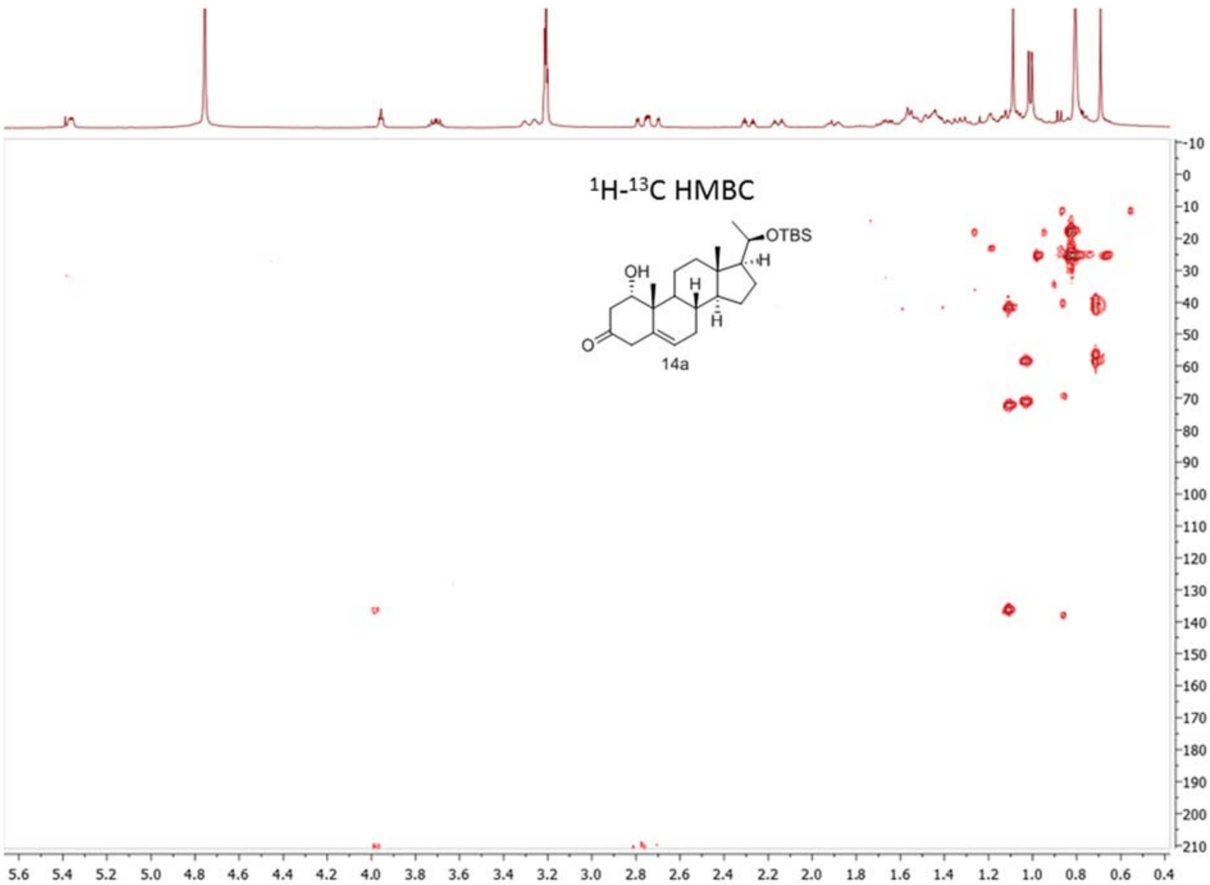

390

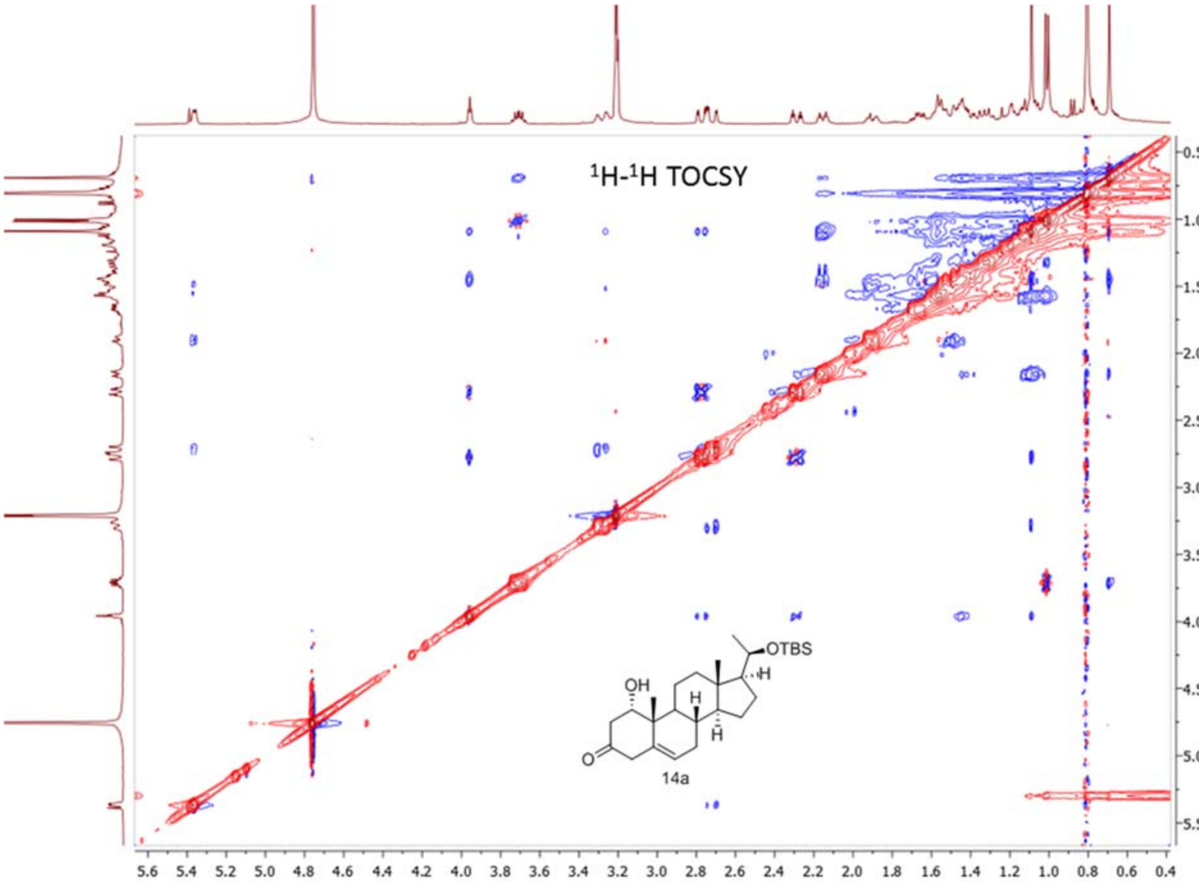

391

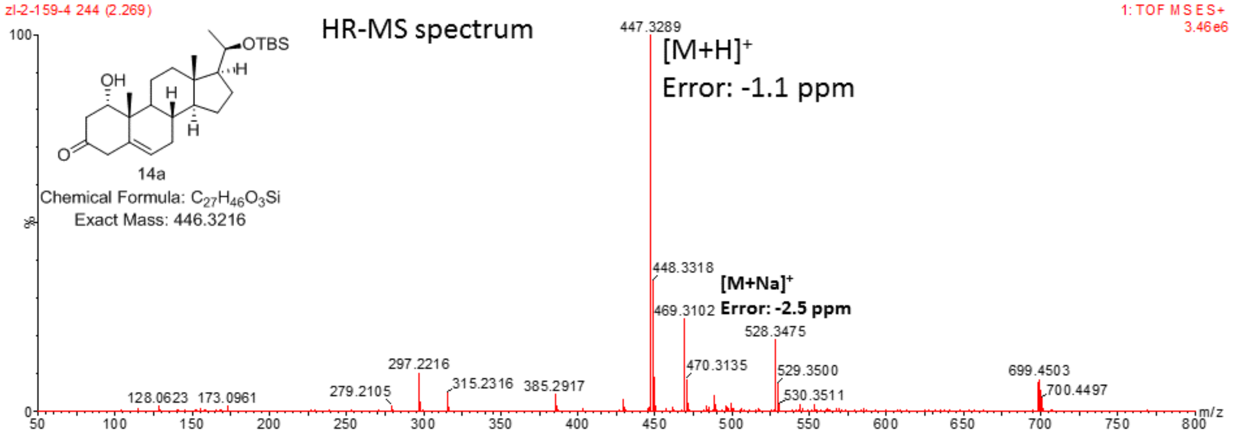
zl-2-159-4 244 (2.269)
1: TOF MS ES+
3.46e6
HR-MS spectrum
OTBS
OH
H
H
H
O
14a
Chemical Formula: $C_{27}H_{46}O_3Si$
Exact Mass: 446.3216
447.3289
[M+H]+
Error: -1.1 ppm
448.3318
[M+Na]+
Error: -2.5 ppm
469.3102
528.3475
470.3135
529.3500
530.3511
297.2216
315.2316
385.2917
279.2105
128.0623
173.0961
699.4503
700.4497
%
m/z

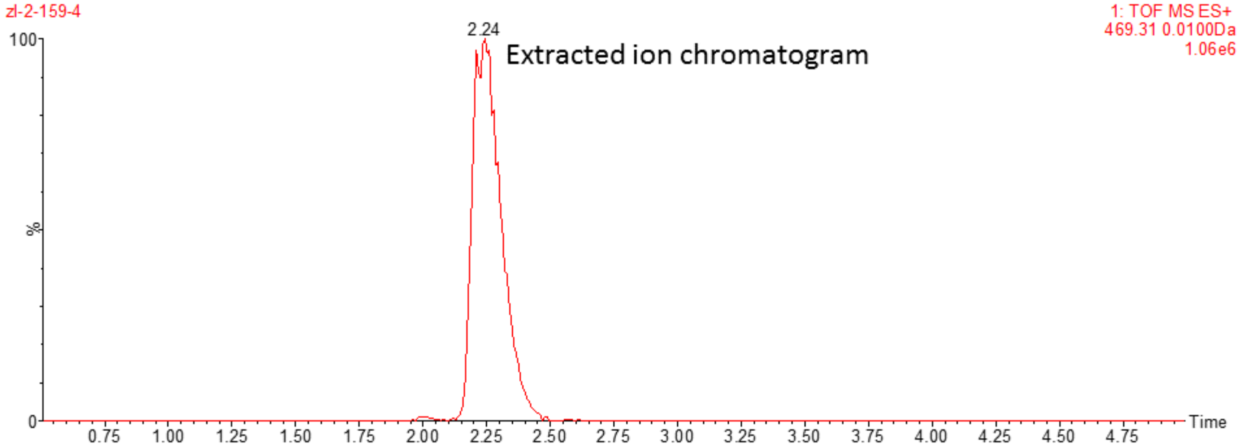
zl-2-159-4
1: TOF MS ES+
469.31 0.0100Da
1.06e6
2.24
Extracted ion chromatogram
%
Time

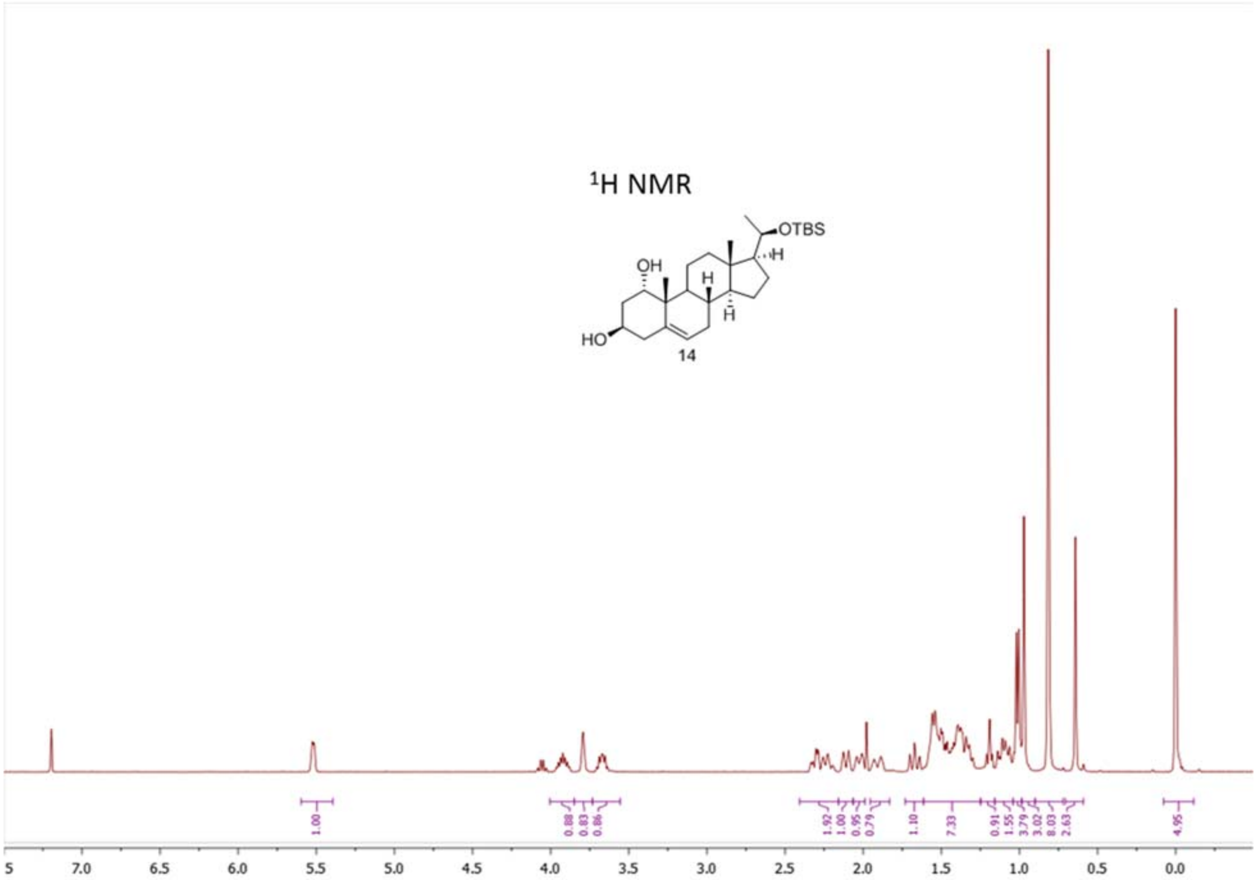
^{1}H NMR
OTBS
OH
HO
14
1.00
0.88
0.83
0.86
1.92
1.00
0.95
0.79
1.10
7.33
0.91
1.55
3.79
3.02
8.03
2.63
4.95
7.0
6.5
6.0
5.5
5.0
4.5
4.0
3.5
3.0
2.5
2.0
1.5
1.0
0.5
0.0

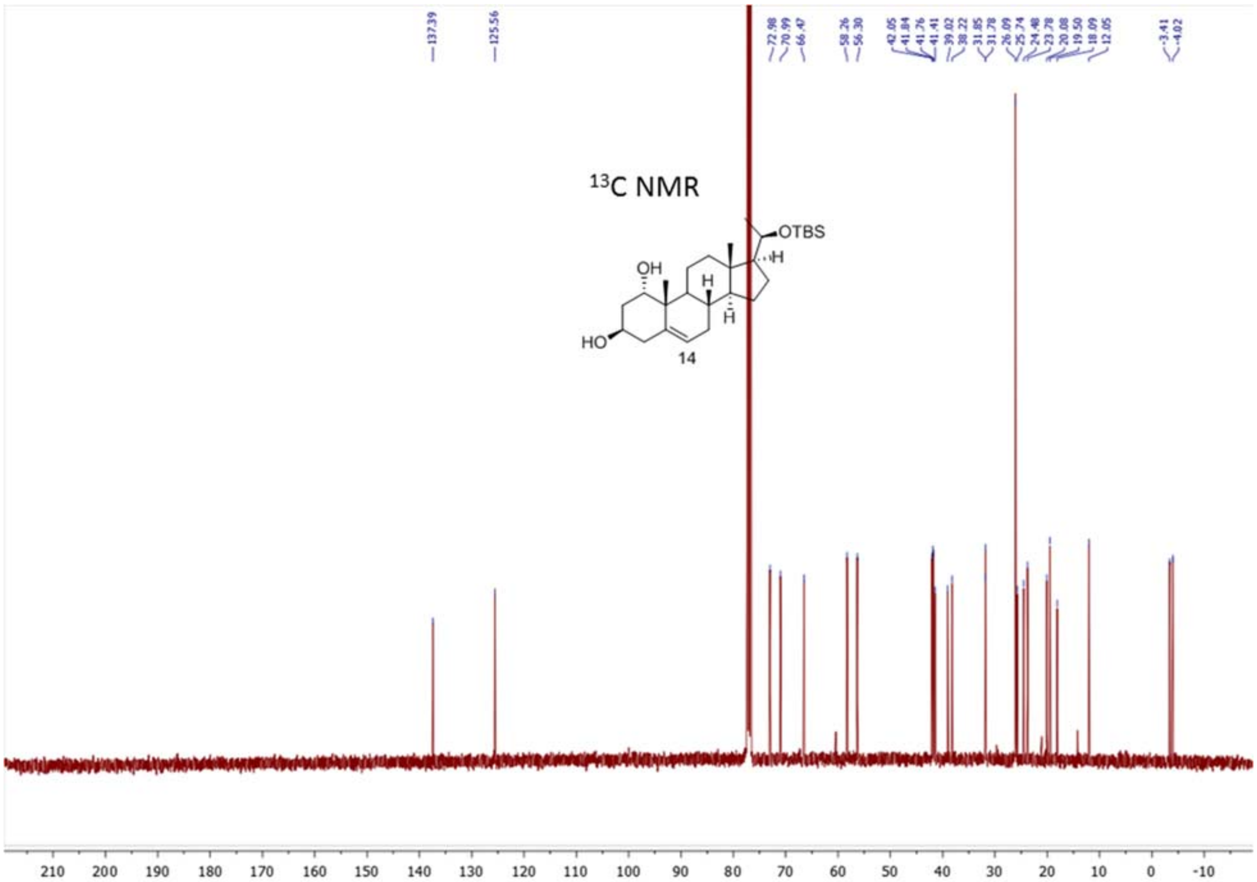

394

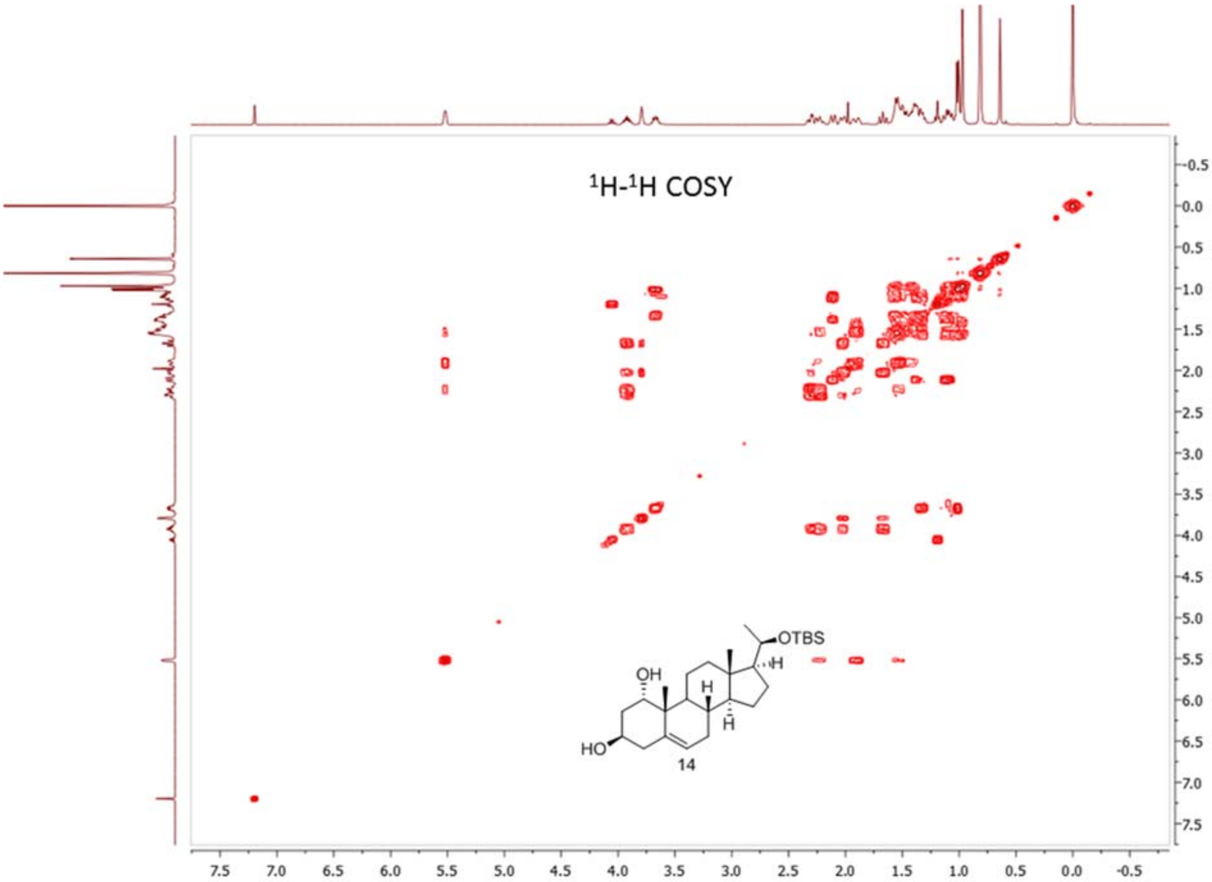

395

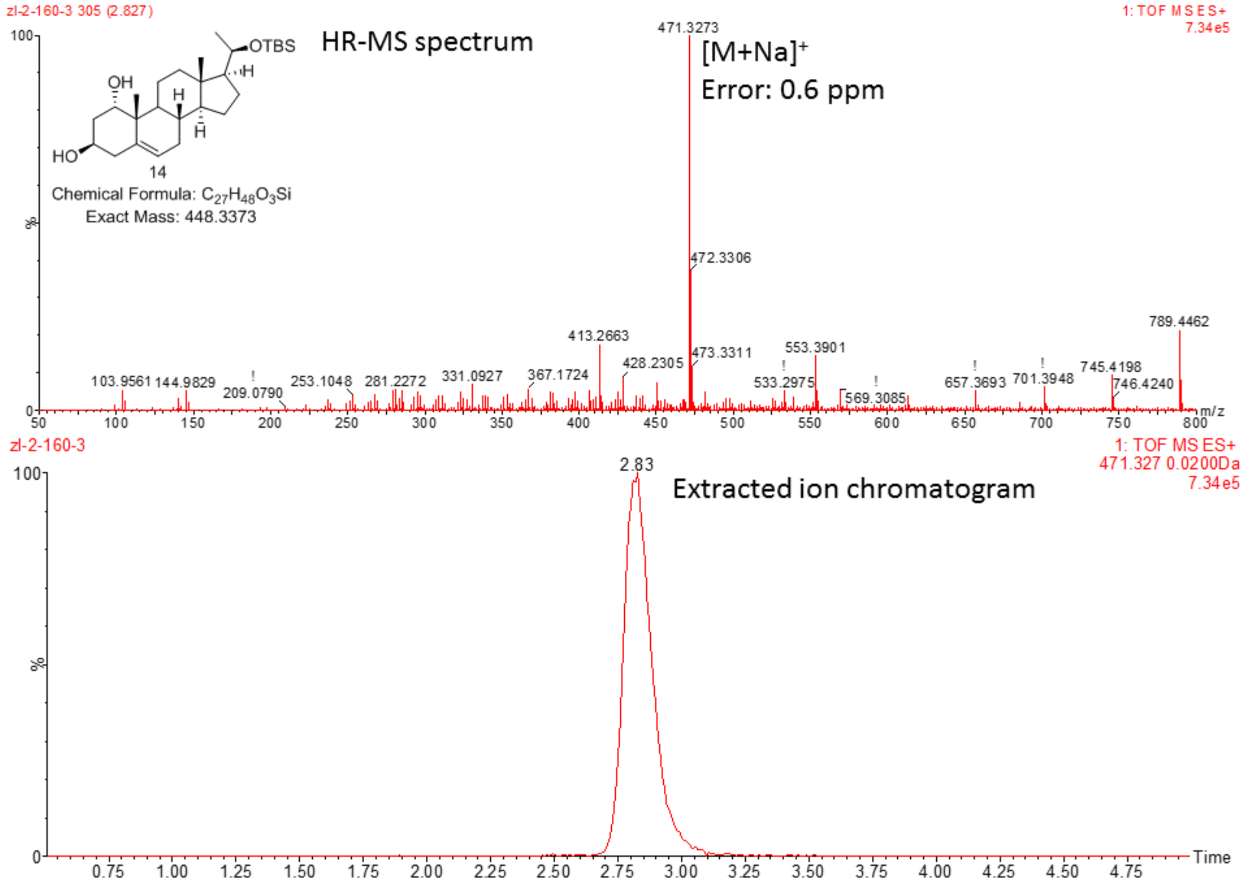

zl-2-160-3 305 (2.827)
1: TOF MS ES+
7.34e5
HR-MS spectrum
[M+Na]+
Error: 0.6 ppm
14
Chemical Formula: C27H48O3Si
Exact Mass: 448.3373
471.3273
472.3306
789.4462
413.2663
428.2305
473.3311
553.3901
533.2975
569.3085
657.3693
701.3948
745.4198
746.4240
103.9561
144.9829
209.0790
253.1048
281.2272
331.0927
367.1724
m/z
zl-2-160-3
1: TOF MS ES+
471.327 0.0200Da
7.34e5
2.83
Extracted ion chromatogram
Time

396

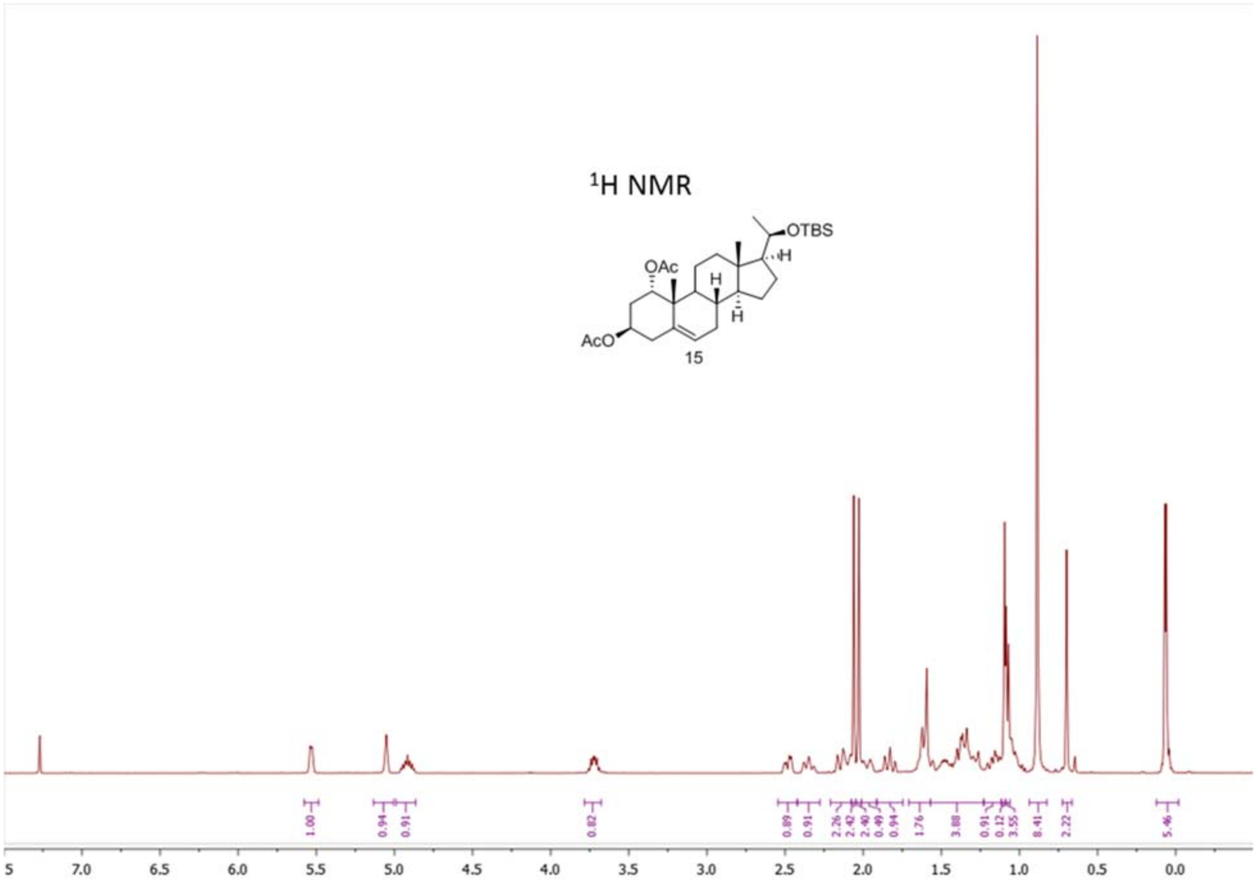

^{1}H NMR
OTBS
OAc
H
AcO
15
1.00
0.94
0.91
0.82
0.89
0.91
2.26
2.42
2.40
0.49
0.94
1.76
3.88
0.91
0.12
3.55
8.41
2.22
5.46
7.0
6.5
6.0
5.5
5.0
4.5
4.0
3.5
3.0
2.5
2.0
1.5
1.0
0.5
0.0

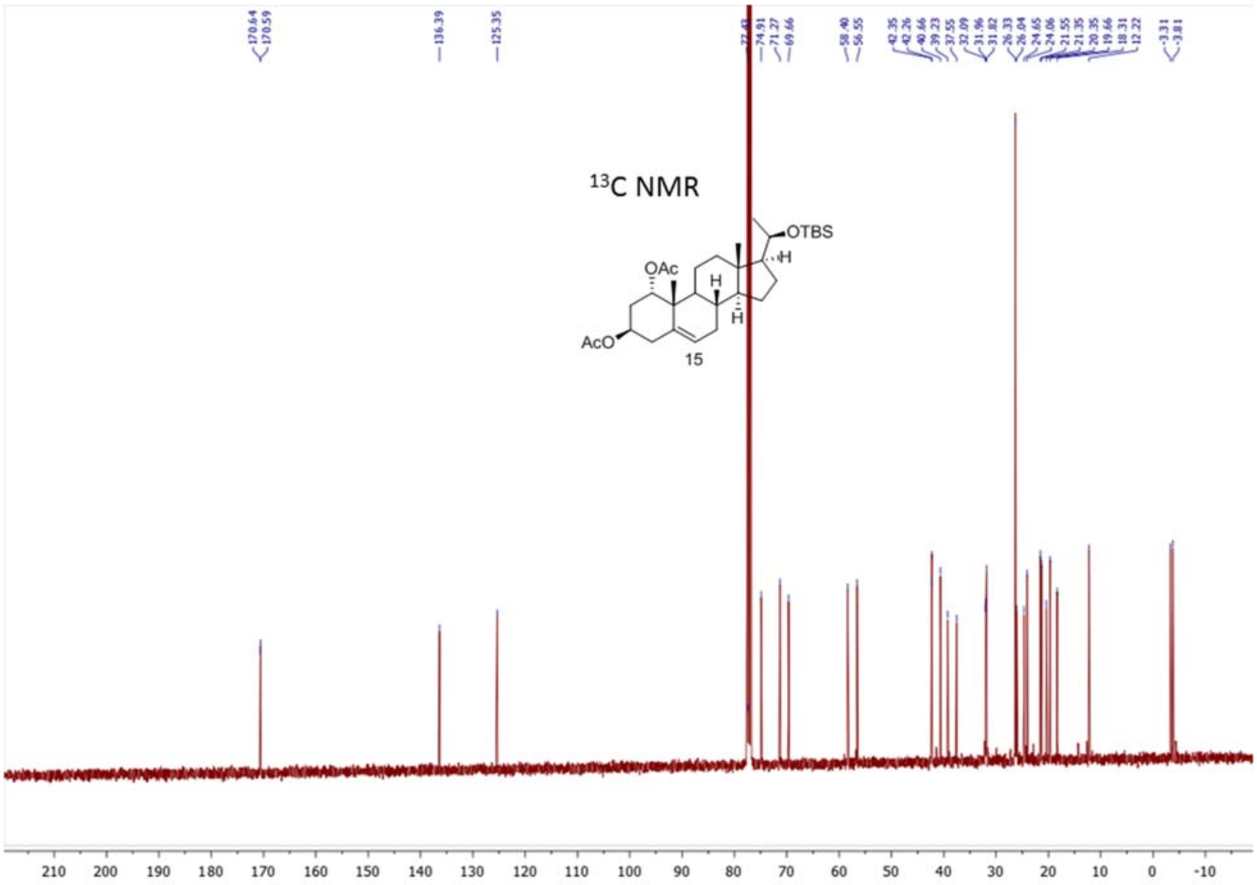
170.64
170.59
136.39
125.35
77.43
74.91
71.27
69.66
58.40
56.55
42.35
42.26
40.66
39.23
37.55
32.09
31.96
31.82
26.33
26.04
24.65
24.06
21.55
21.35
20.35
19.66
18.31
12.22
-3.31
-3.81
13C NMR
OTBS
OAc
H
H
H
AcO
15
210 200 190 180 170 160 150 140 130 120 110 100 90 80 70 60 50 40 30 20 10 0 -10

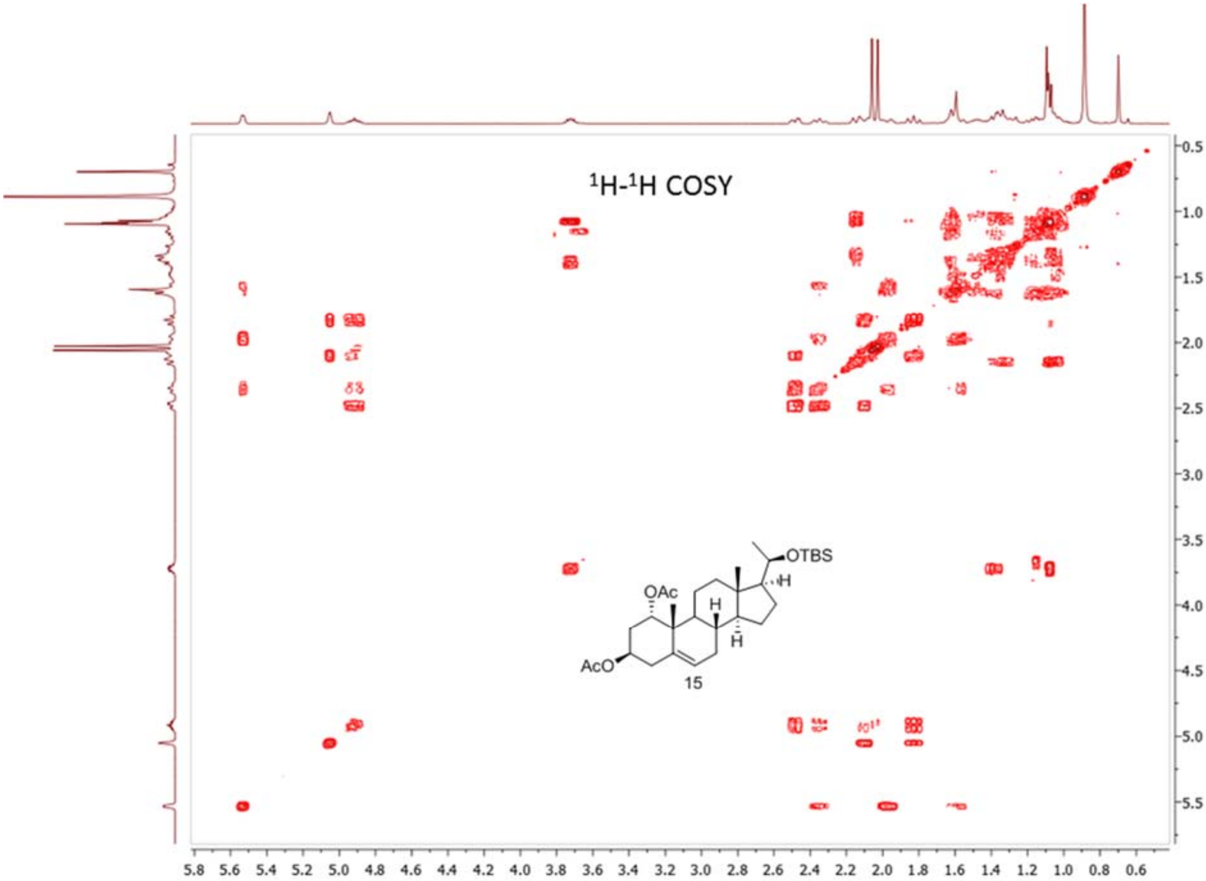
^1H-^1H COSY
OAc
OTBS
H
AcO
15
5.8 5.6 5.4 5.2 5.0 4.8 4.6 4.4 4.2 4.0 3.8 3.6 3.4 3.2 3.0 2.8 2.6 2.4 2.2 2.0 1.8 1.6 1.4 1.2 1.0 0.8 0.6
0.5 1.0 1.5 2.0 2.5 3.0 3.5 4.0 4.5 5.0 5.5

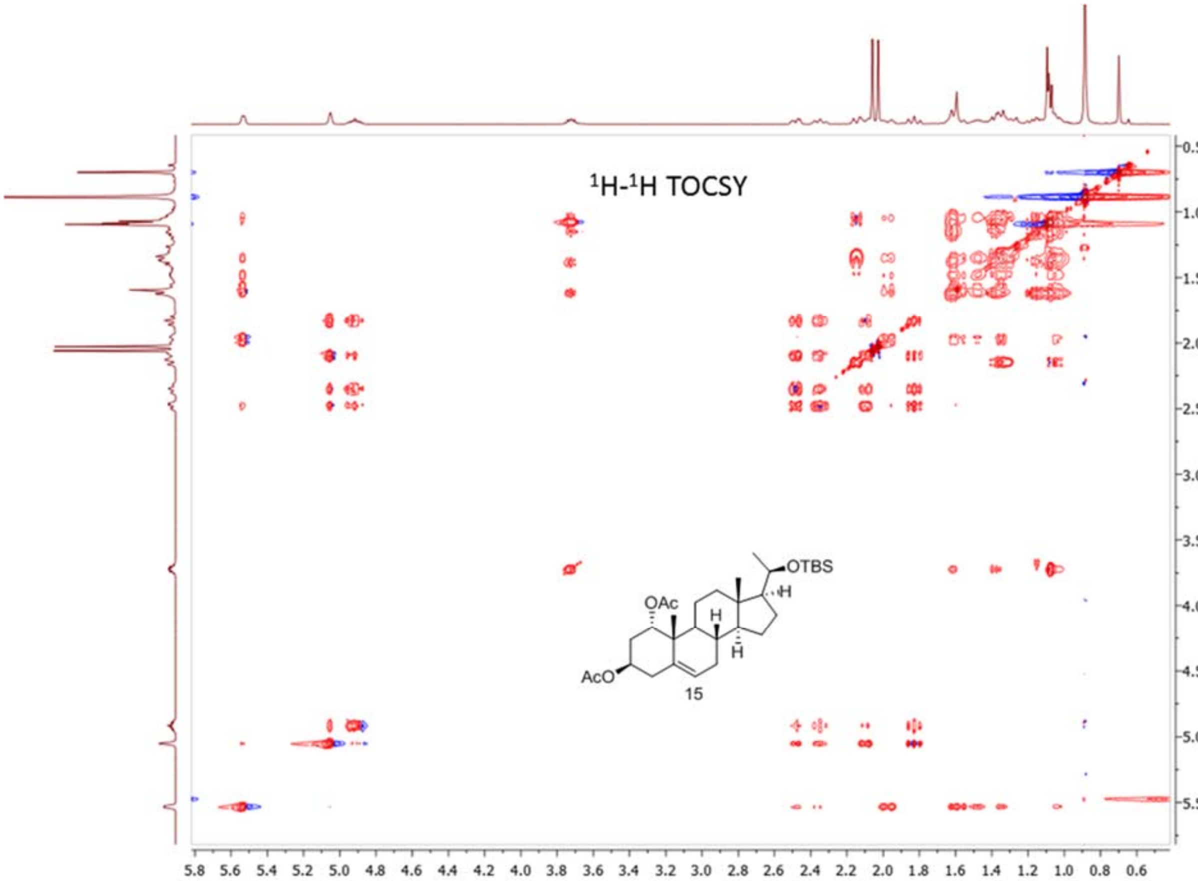
^{1}H-^{1}H TOCSY
OAc
OTBS
H
H
H
AcO
15
5.8 5.6 5.4 5.2 5.0 4.8 4.6 4.4 4.2 4.0 3.8 3.6 3.4 3.2 3.0 2.8 2.6 2.4 2.2 2.0 1.8 1.6 1.4 1.2 1.0 0.8 0.6
0.5 1.0 1.5 2.0 2.5 3.0 3.5 4.0 4.5 5.0 5.5

400

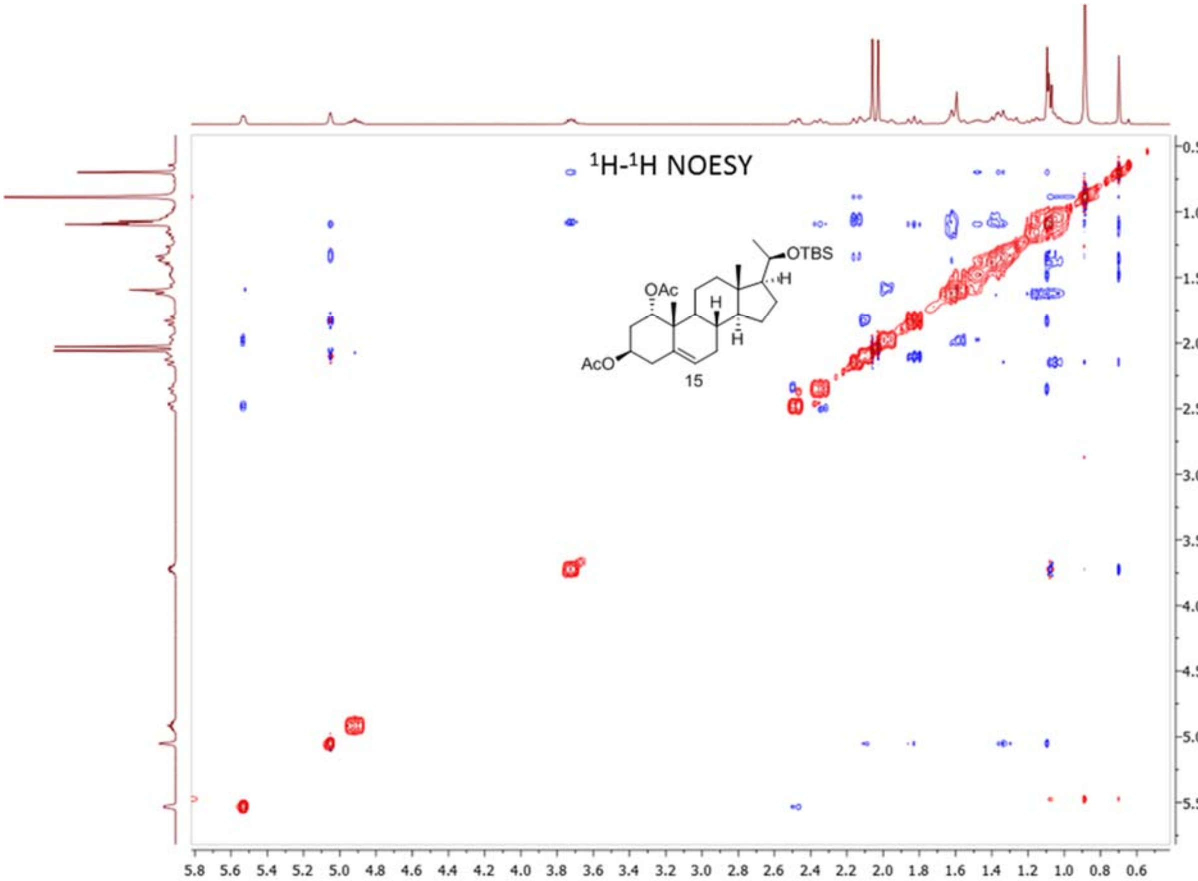

401

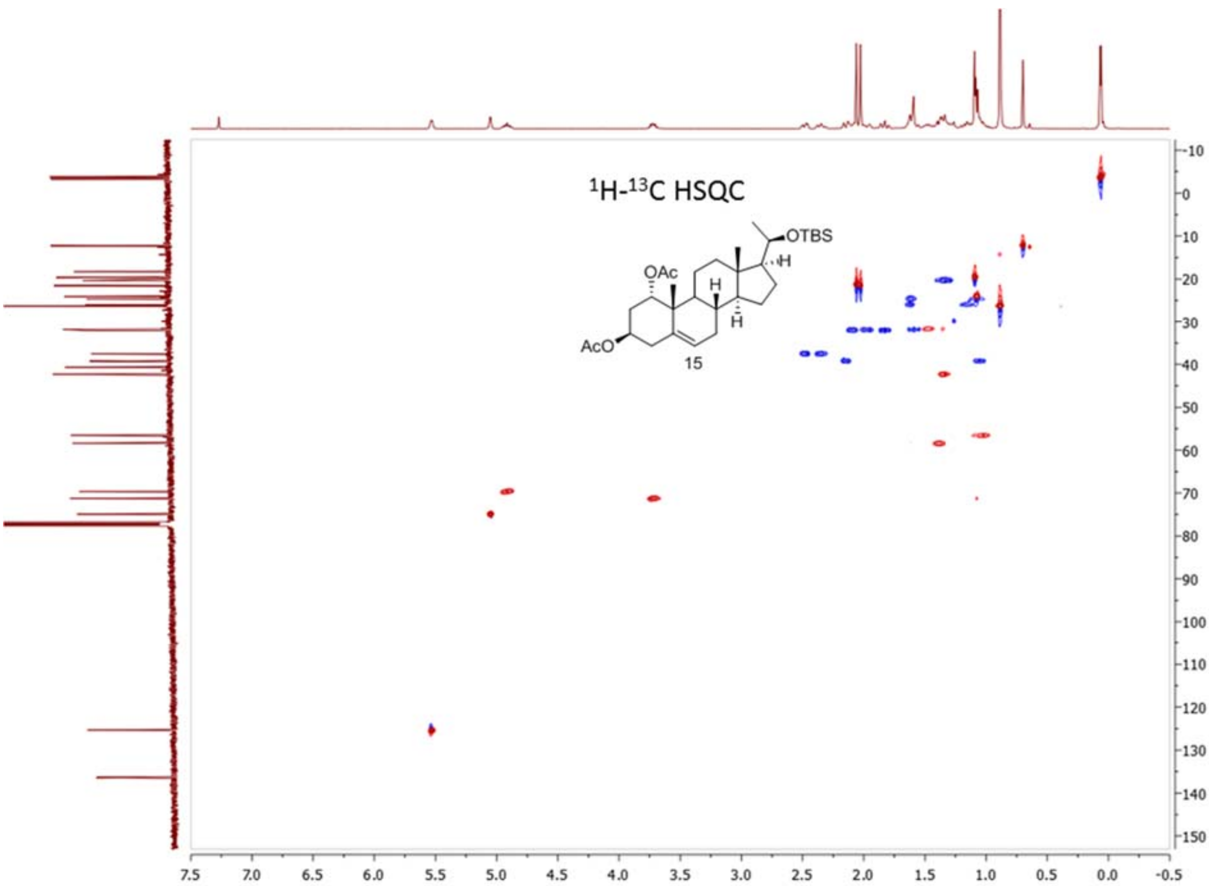

402

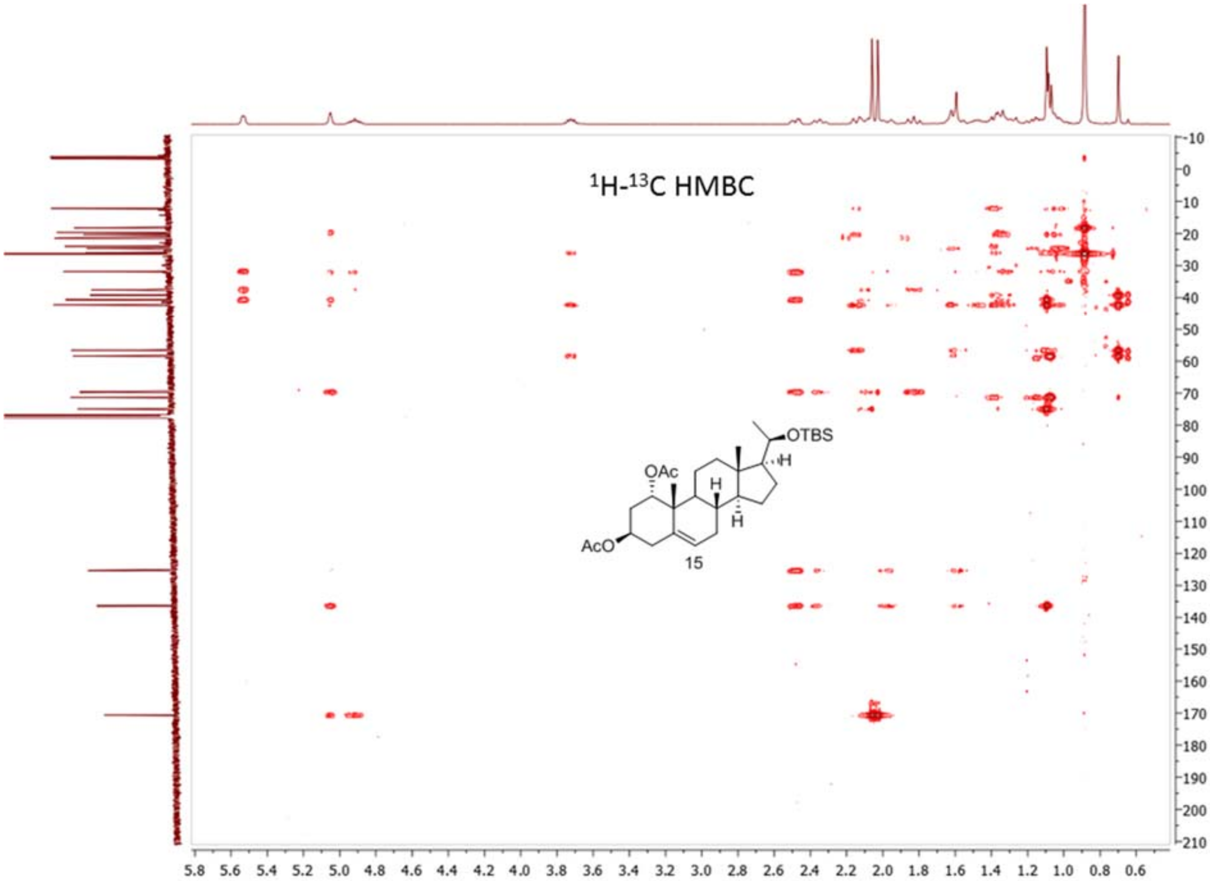

403

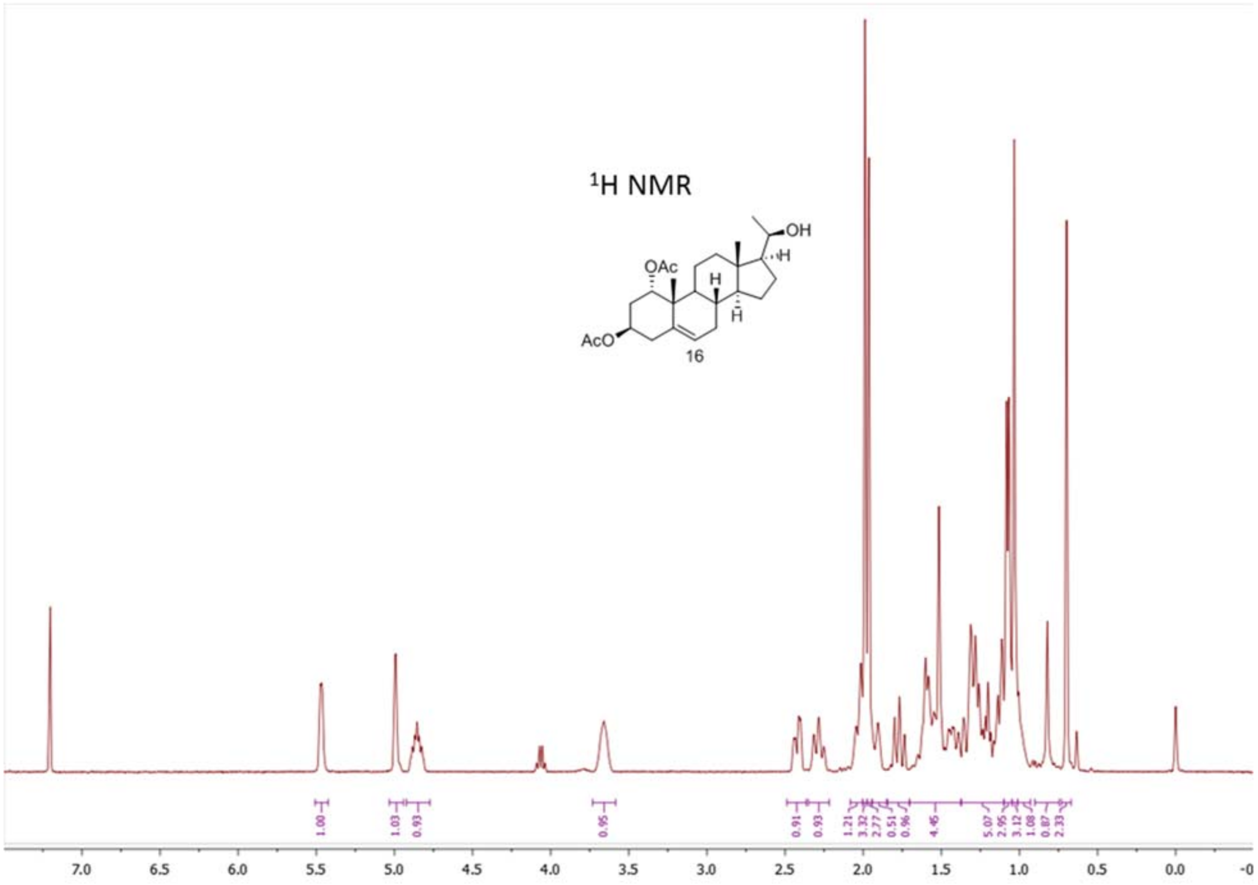

1H NMR
OAc
OH
H
H
H
AcO
16
1.00
1.03
0.93
0.95
0.91
0.93
1.21
3.32
2.77
0.51
0.96
4.45
5.07
2.95
3.12
1.08
0.87
2.33
7.0
6.5
6.0
5.5
5.0
4.5
4.0
3.5
3.0
2.5
2.0
1.5
1.0
0.5
0.0

404

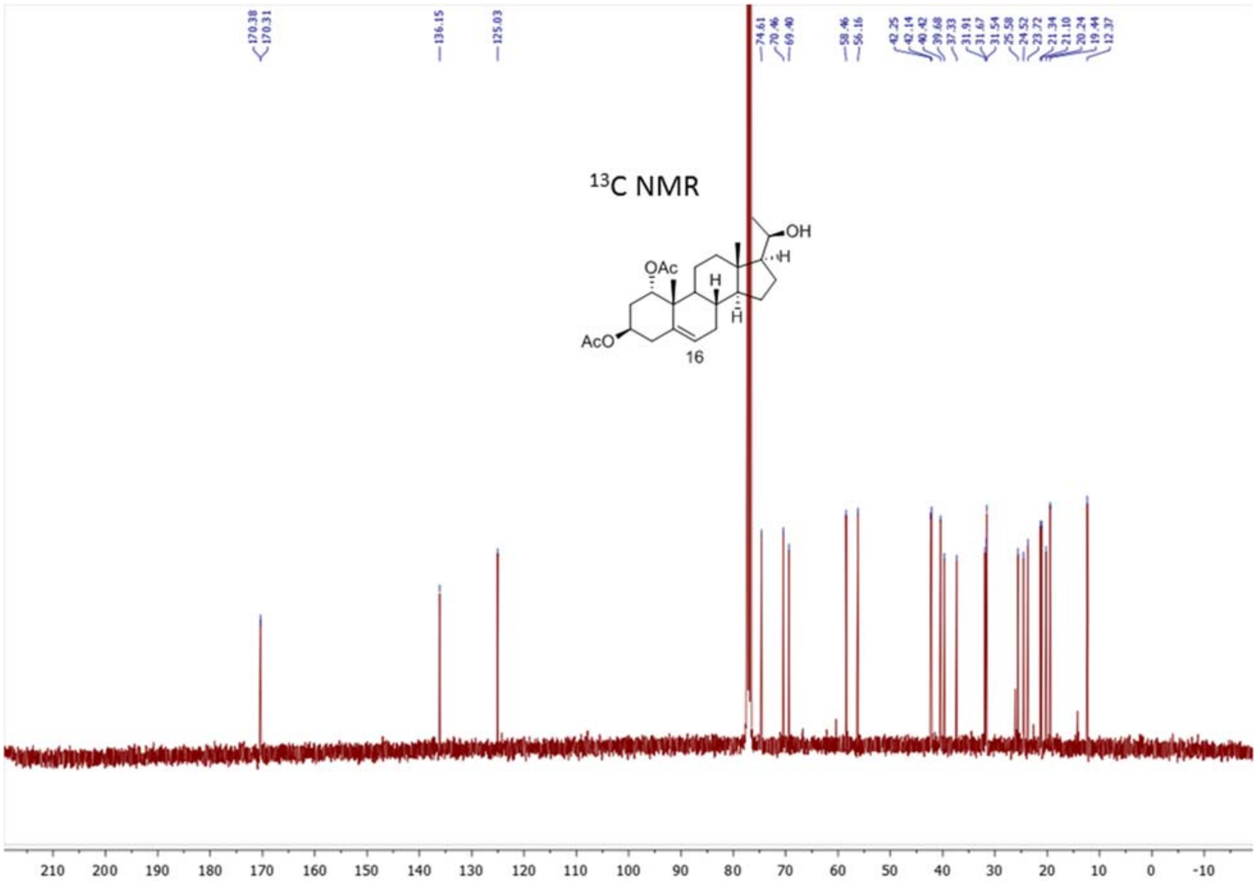
170.38
170.31
136.15
125.03
74.61
70.46
69.40
58.46
56.16
42.25
42.14
40.42
39.68
37.33
31.91
31.67
31.54
25.58
24.52
23.72
21.34
21.10
20.24
19.44
12.37
13C NMR
OH
H
OAc
H
H
AcO
16
210 200 190 180 170 160 150 140 130 120 110 100 90 80 70 60 50 40 30 20 10 0 -10

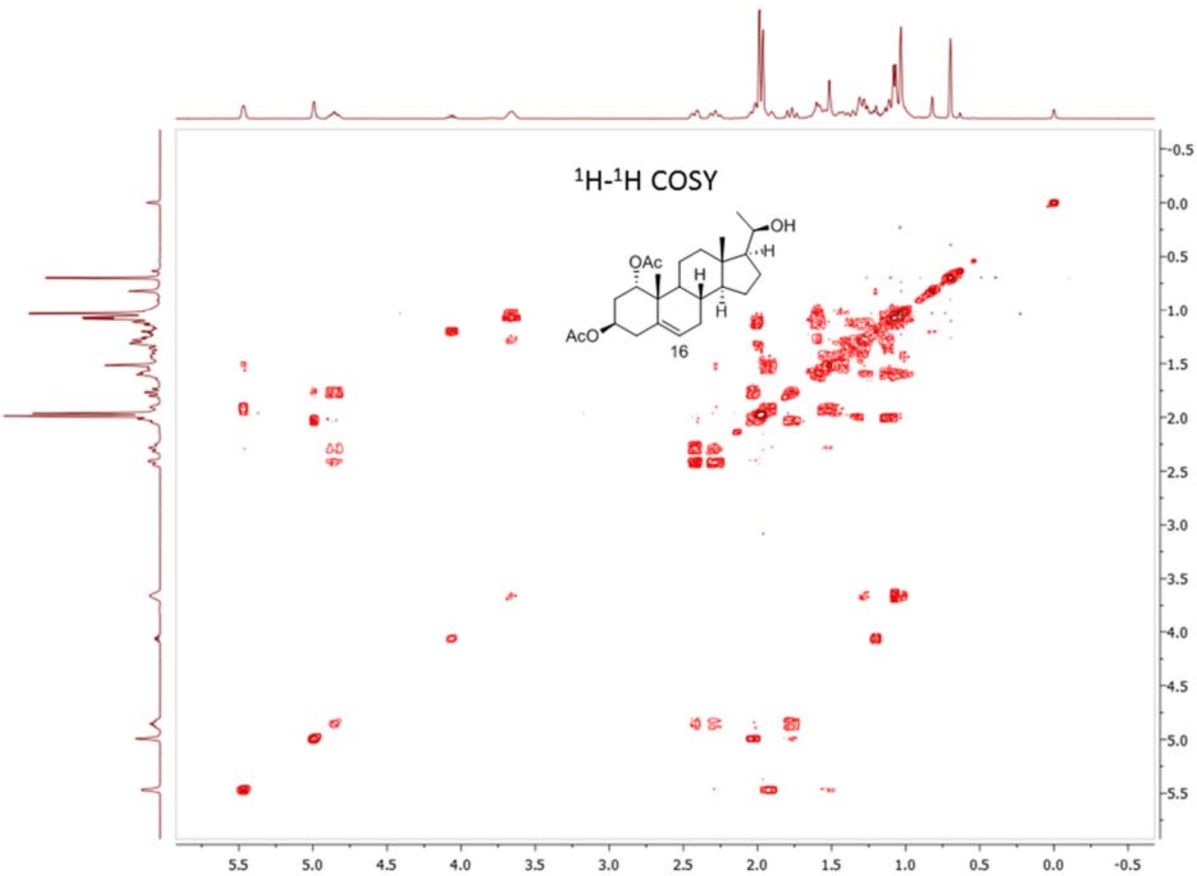

406

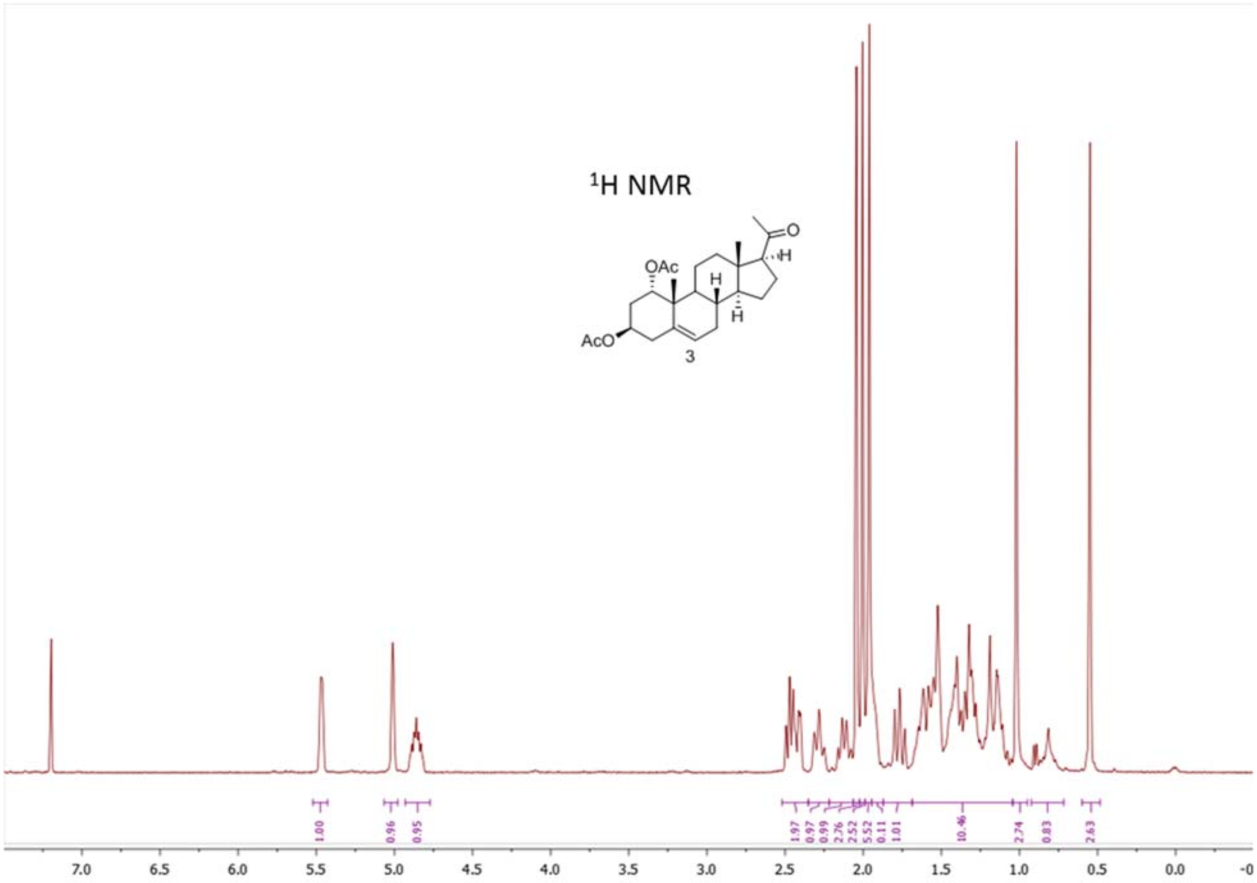

1H NMR
OAc
AcO
H
3
1.00
0.96
0.95
1.97
0.97
0.99
2.76
2.52
5.52
0.11
1.01
10.46
2.74
0.83
2.63
7.0
6.5
6.0
5.5
5.0
4.5
4.0
3.5
3.0
2.5
2.0
1.5
1.0
0.5
0.0

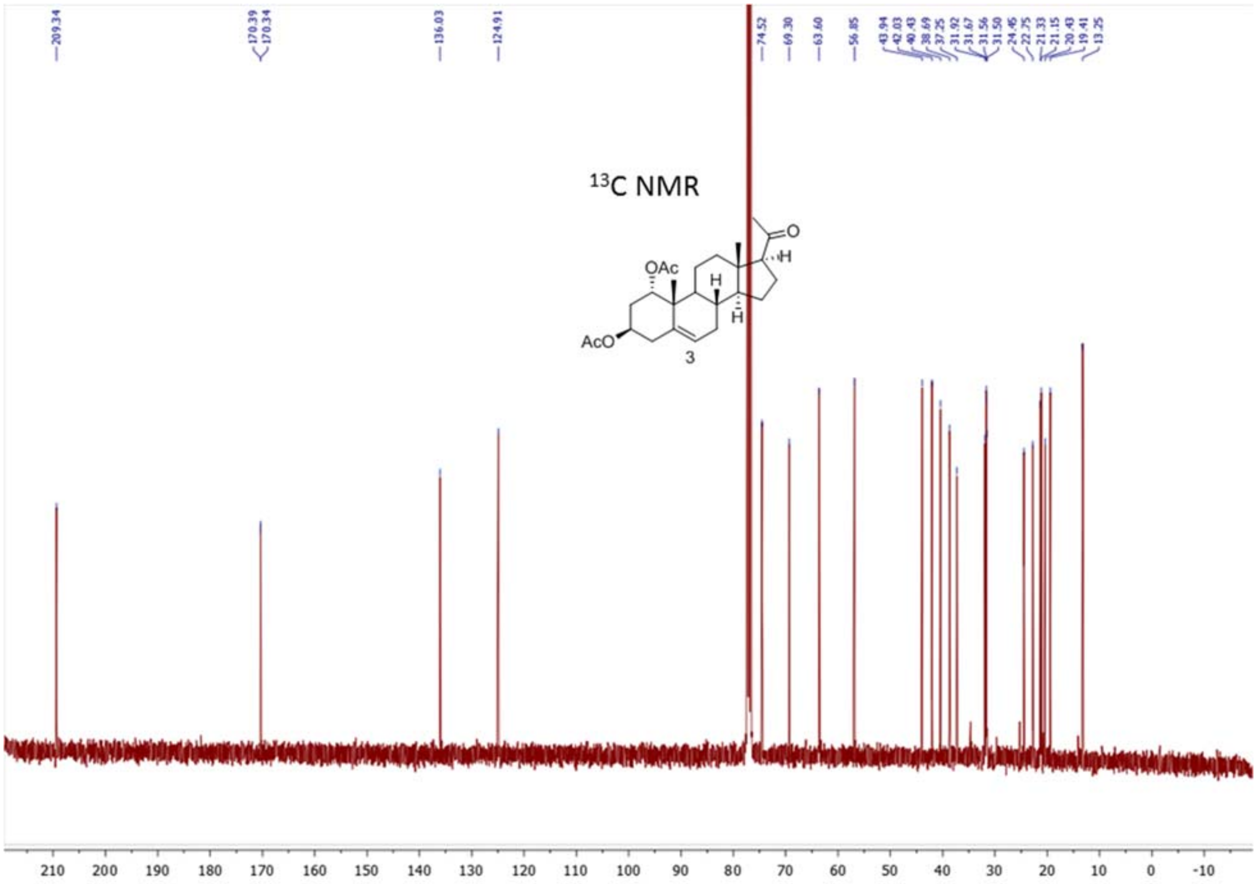

209.34
170.39
170.34
136.03
124.91
74.52
69.30
63.60
56.85
43.94
42.03
40.43
38.69
37.25
31.92
31.67
31.56
31.50
24.46
22.75
21.33
21.15
20.43
19.41
13.25
13C NMR
OAc
H
H
H
AcO
3
210
200
190
180
170
160
150
140
130
120
110
100
90
80
70
60
50
40
30
20
10
0
-10

408

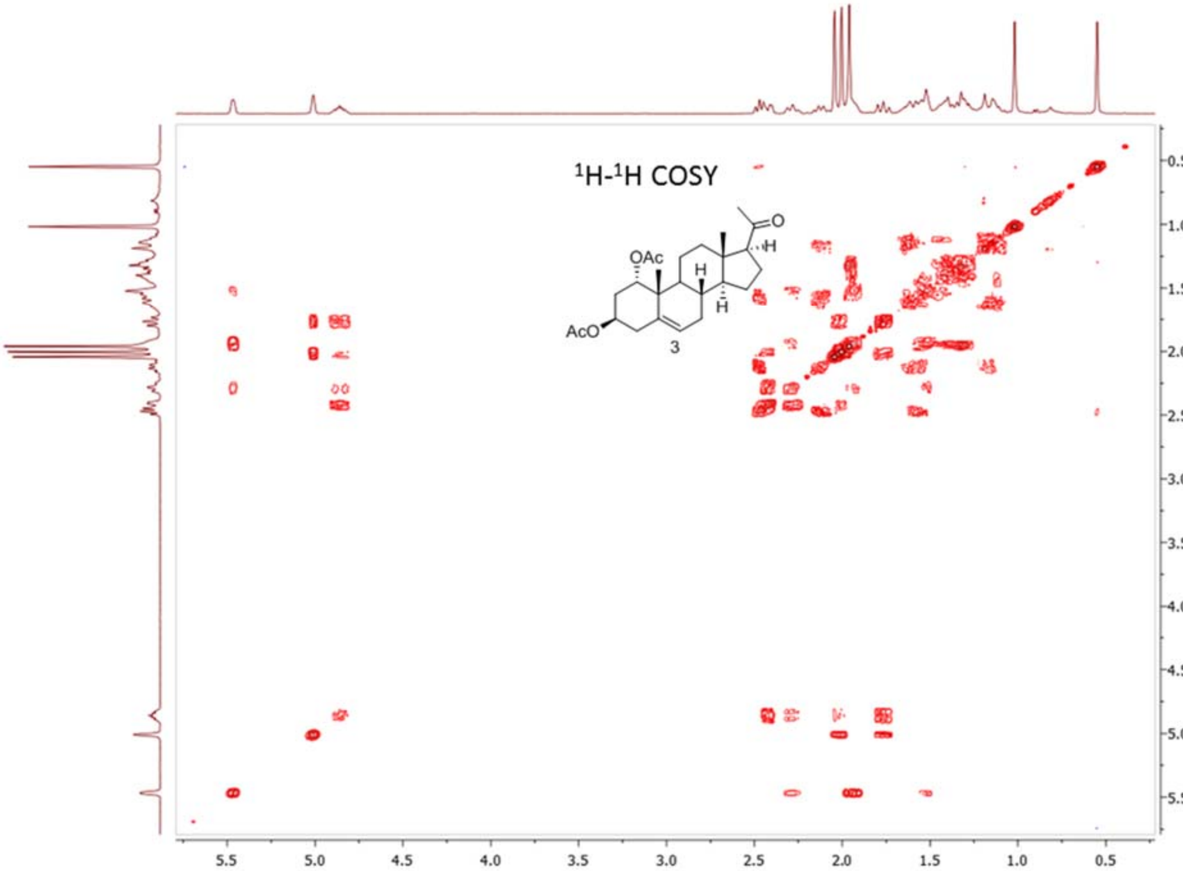

409

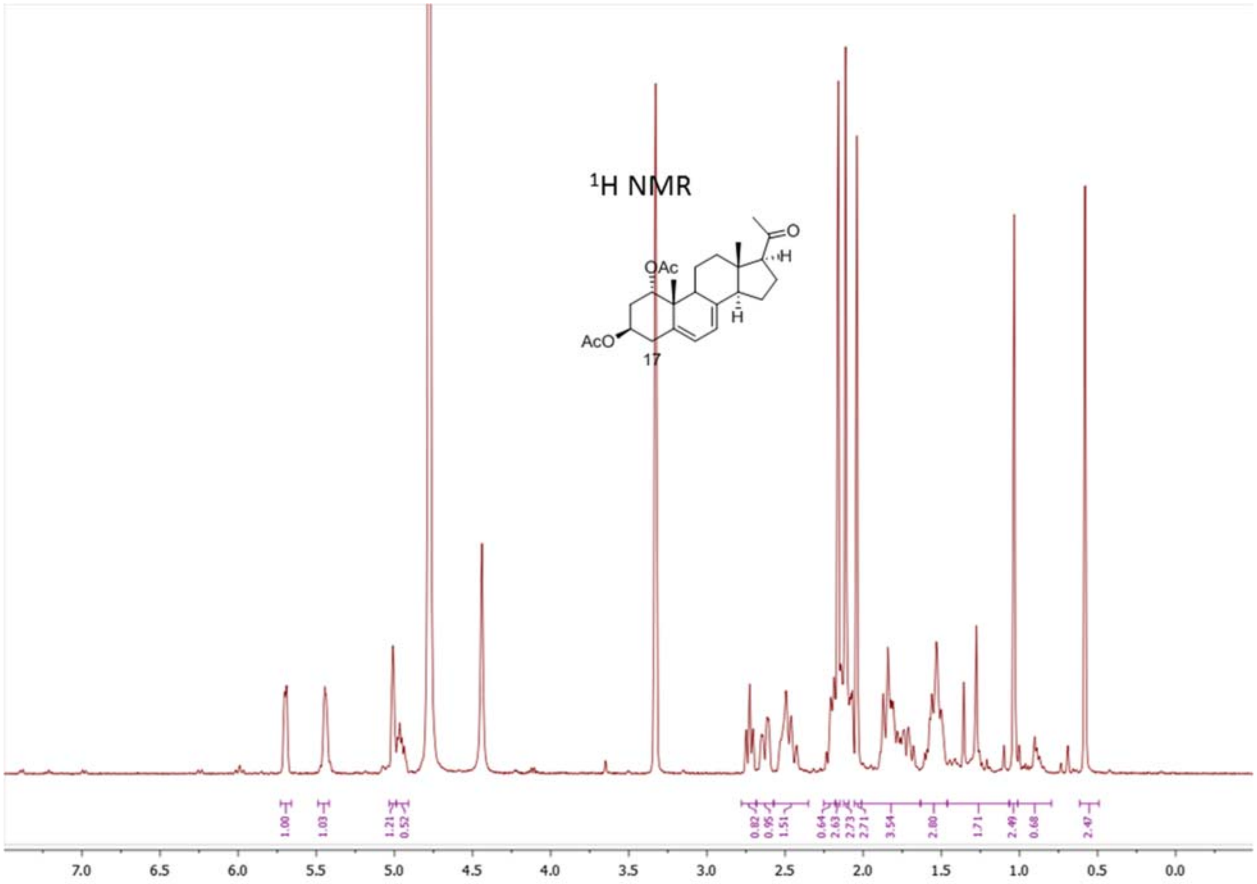

1H NMR
OAc
AcO
H
H
17
1.00
1.03
1.21
0.52
0.82
0.95
1.51
0.64
2.63
2.73
2.71
3.54
2.80
1.71
2.49
0.68
2.47
7.0
6.5
6.0
5.5
5.0
4.5
4.0
3.5
3.0
2.5
2.0
1.5
1.0
0.5
0.0

410

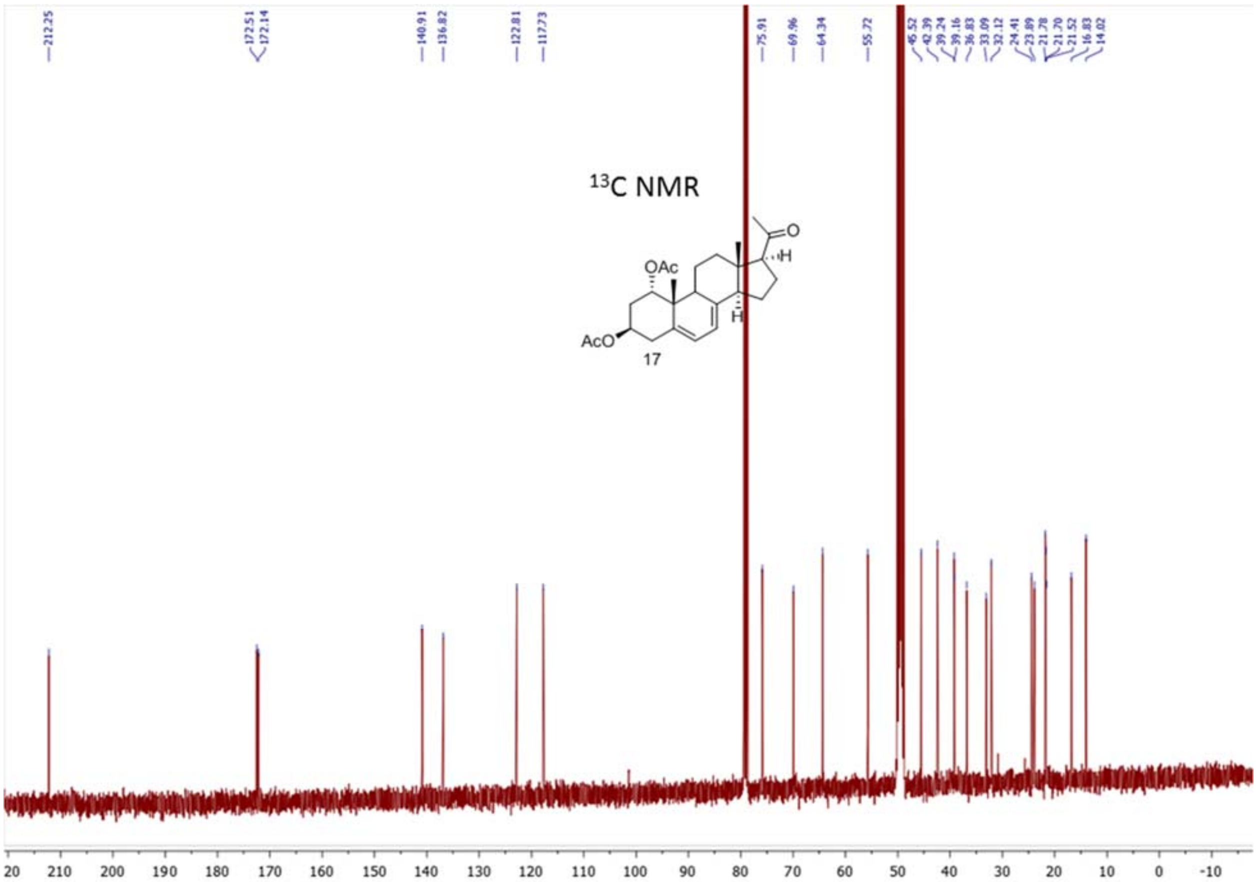
212.25
172.51
172.14
140.91
136.82
122.81
117.73
75.91
69.96
64.34
55.72
45.52
42.39
39.24
39.16
36.83
33.09
32.12
24.41
23.89
21.78
21.70
21.52
16.83
14.02
^{13}C NMR
OAc
H
H
AcO
17
20 210 200 190 180 170 160 150 140 130 120 110 100 90 80 70 60 50 40 30 20 10 0 -10

411

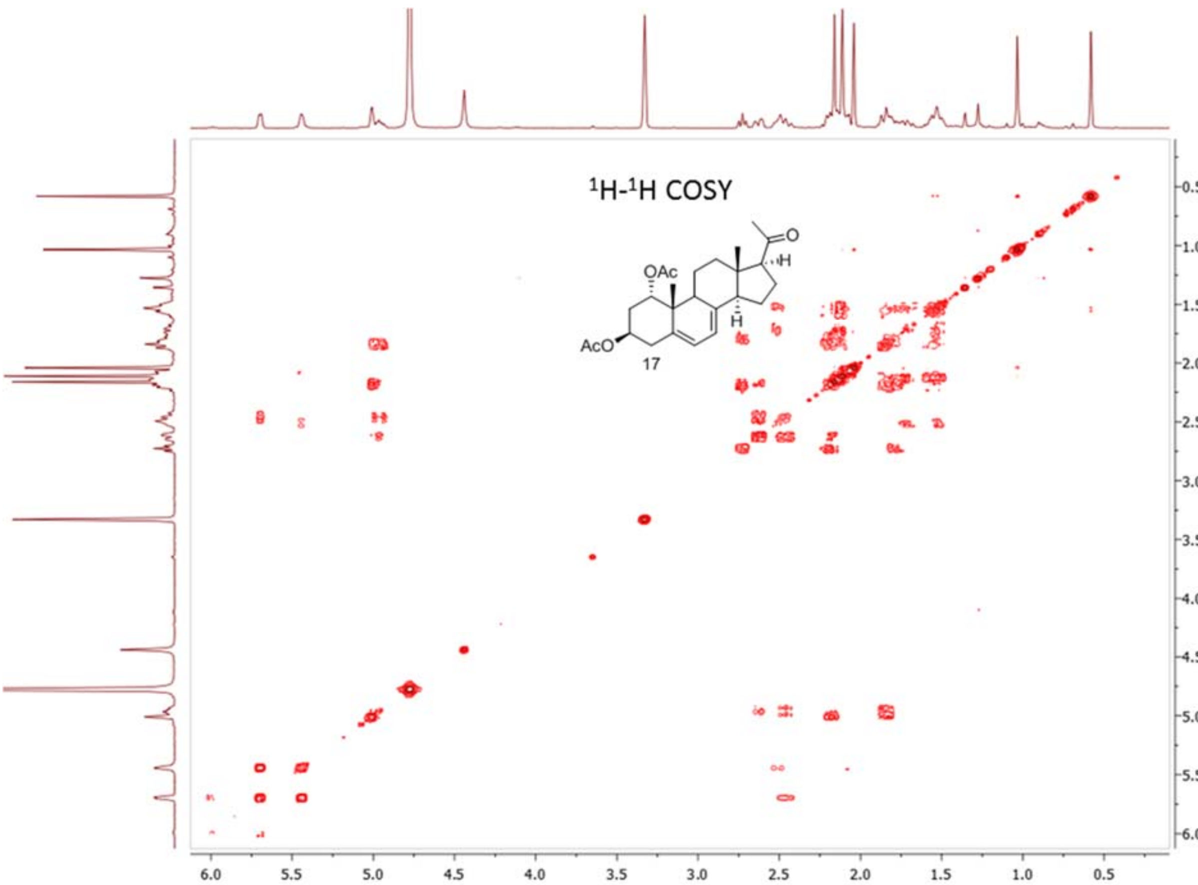

412

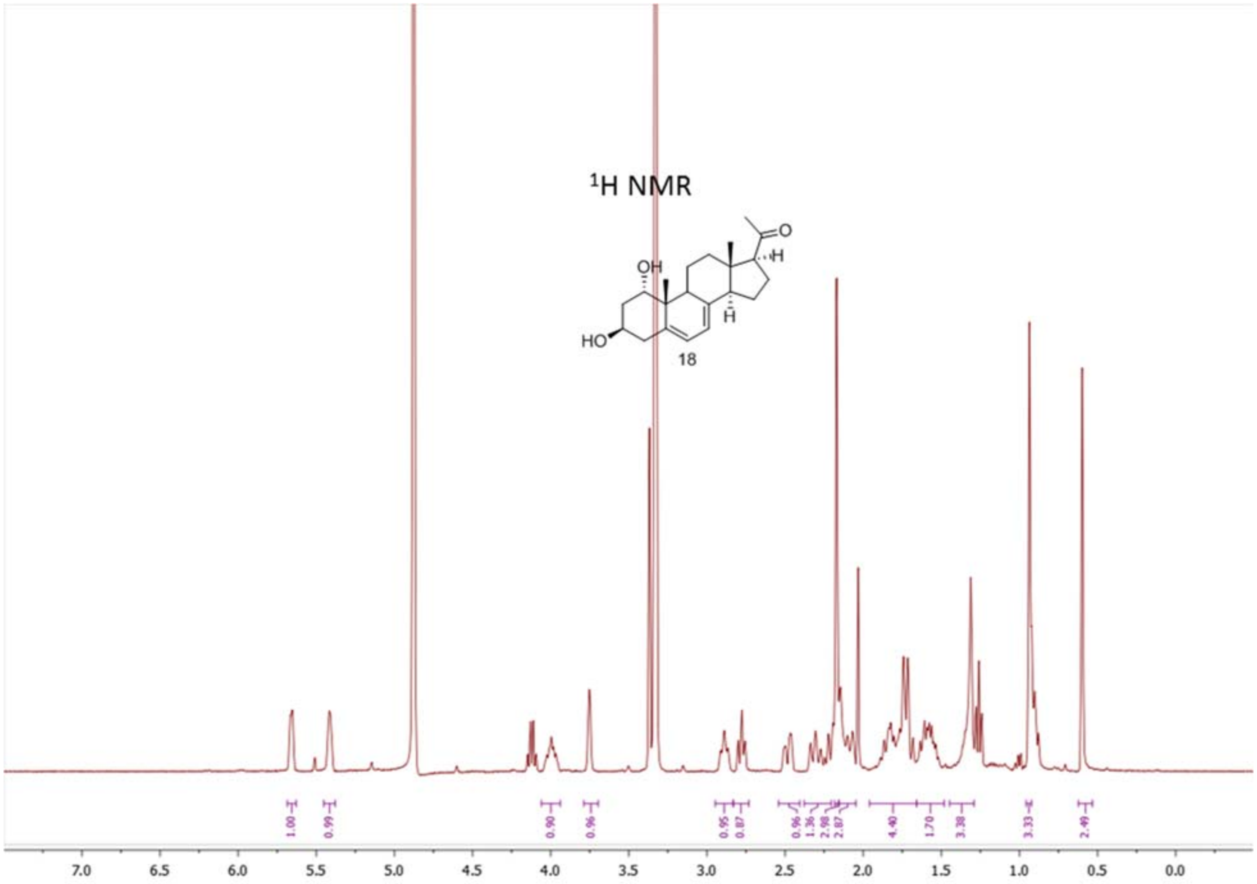

^{1}H NMR
OH
HO
H
H
O
18
1.00
0.99
0.90
0.96
0.95
0.87
0.96
1.36
2.98
2.87
4.40
1.70
3.38
3.33
2.49
7.0
6.5
6.0
5.5
5.0
4.5
4.0
3.5
3.0
2.5
2.0
1.5
1.0
0.5
0.0

413

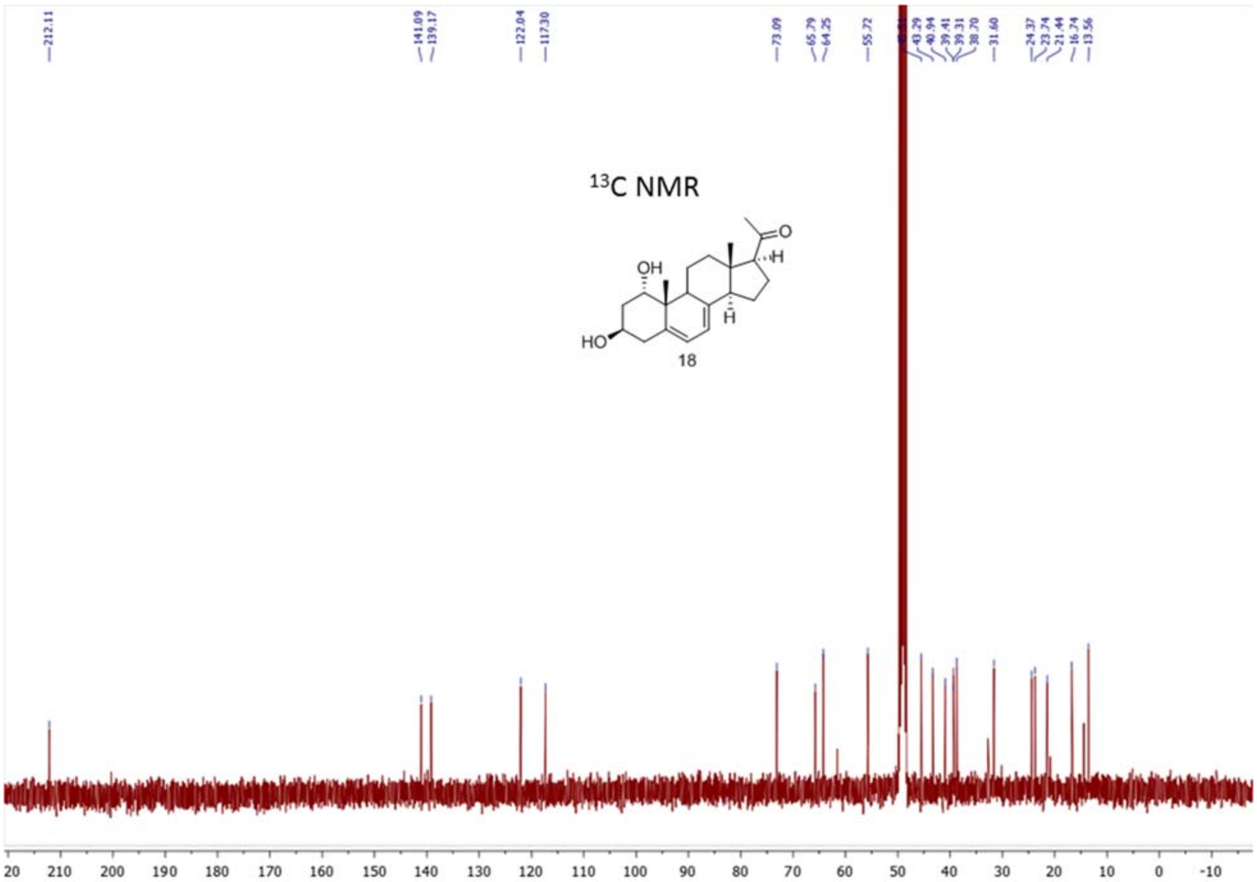
212.11
141.09
139.17
122.04
117.30
73.09
65.79
64.25
55.72
43.29
40.94
39.41
39.31
38.70
31.60
24.37
23.74
21.44
16.74
13.56
^{13}C NMR
OH
HO
H
O
18
20 210 200 190 180 170 160 150 140 130 120 110 100 90 80 70 60 50 40 30 20 10 0 -10

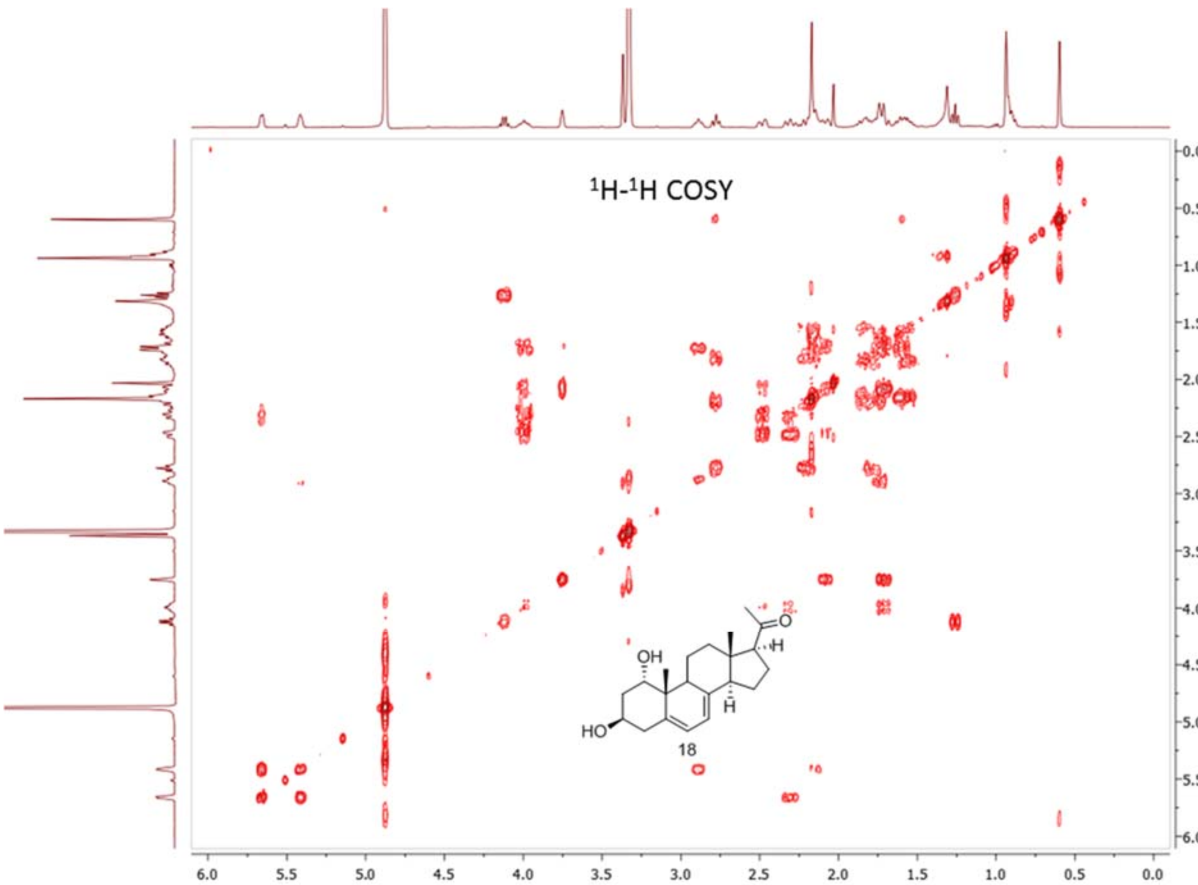

415

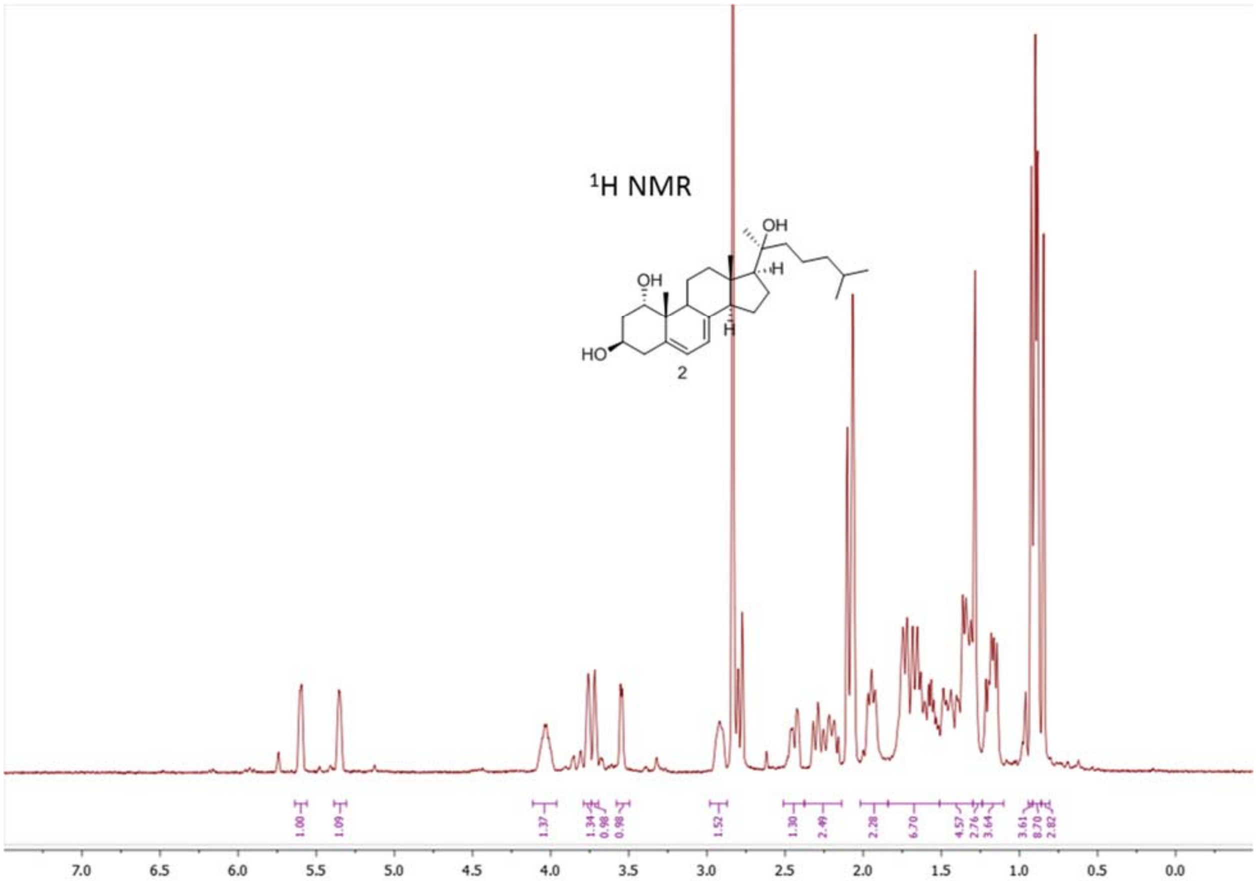

¹H NMR
OH
H
OH
HO
H
2
1.00
1.09
1.37
1.34
0.98
0.98
1.52
1.30
2.49
2.28
6.70
4.57
2.76
3.64
3.61
8.70
2.82
7.0
6.5
6.0
5.5
5.0
4.5
4.0
3.5
3.0
2.5
2.0
1.5
1.0
0.5
0.0

416

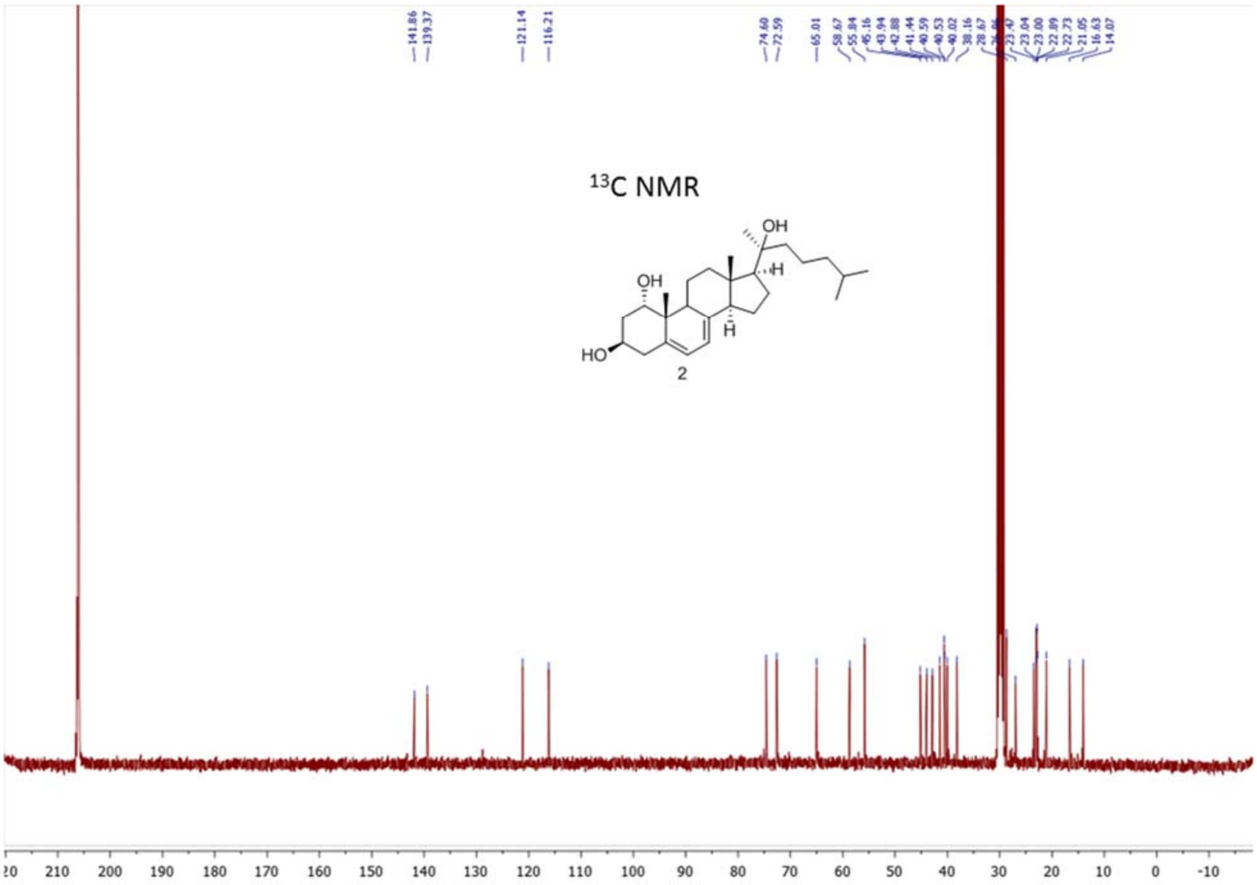
141.86
139.37
121.14
116.21
74.60
72.59
65.01
58.67
55.84
45.16
43.94
42.88
41.44
40.59
40.53
40.02
38.16
28.67
23.47
23.04
23.00
22.89
22.73
21.05
16.63
14.07
13C NMR
OH
OH
H
H
HO
2
210
200
190
180
170
160
150
140
130
120
110
100
90
80
70
60
50
40
30
20
10
0
-10

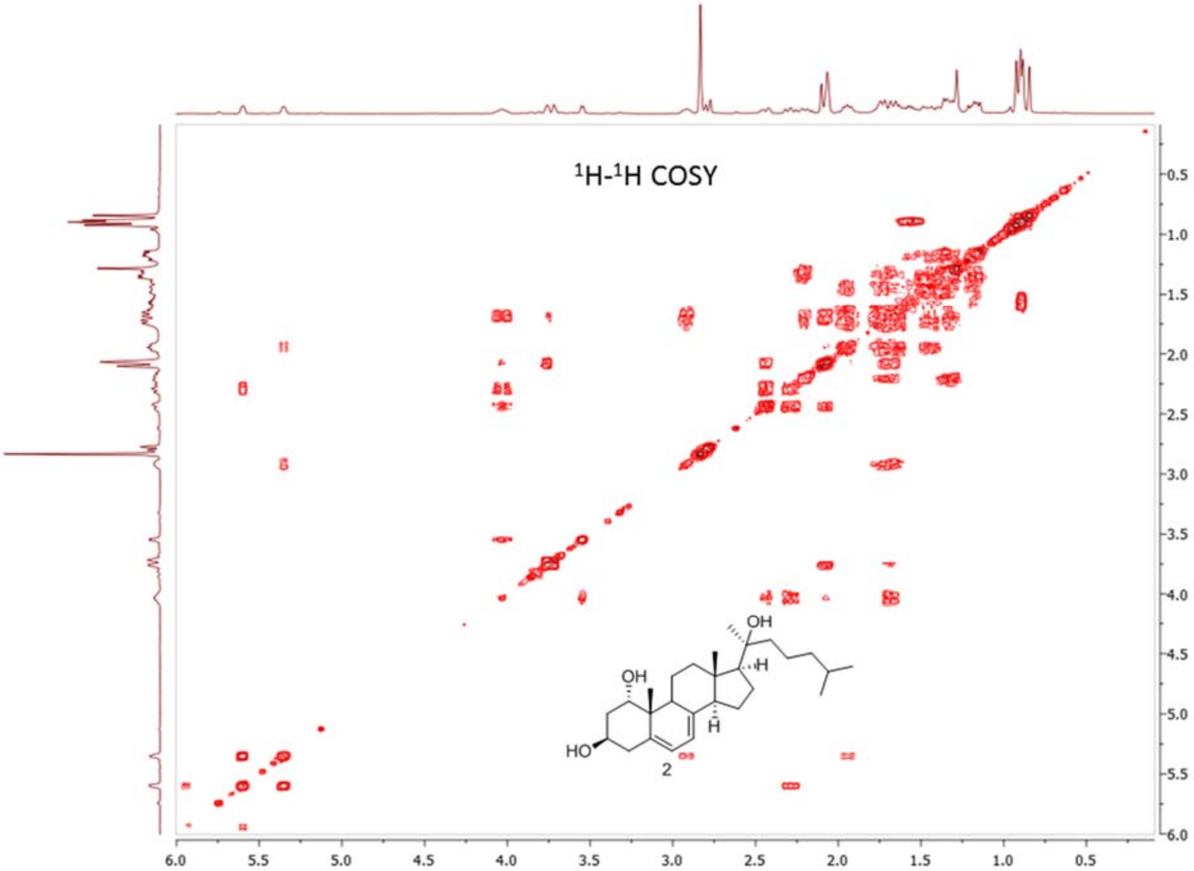

418

HPLC chromatogram

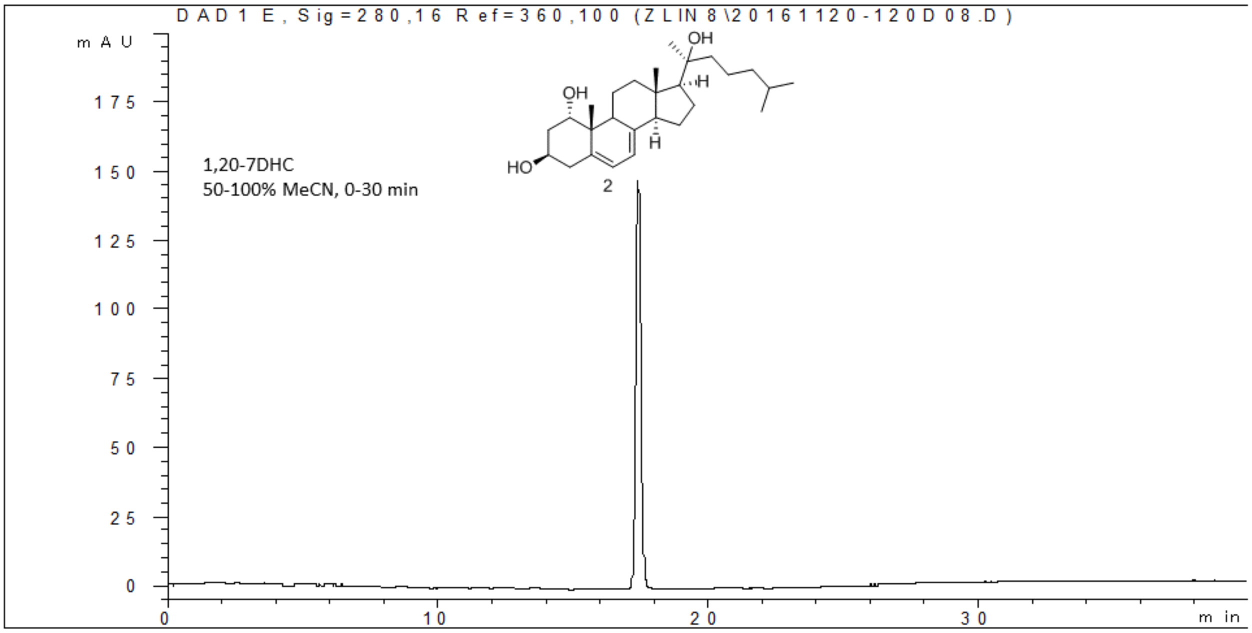

419

UV spectrum

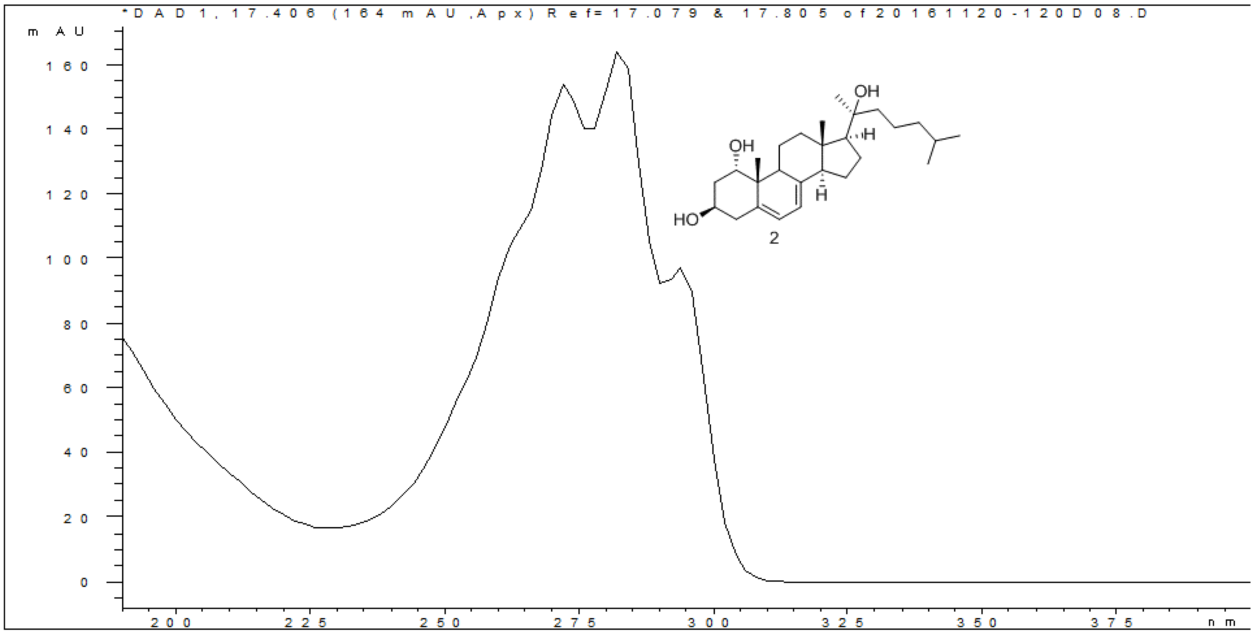

420

HR-MS spectrum

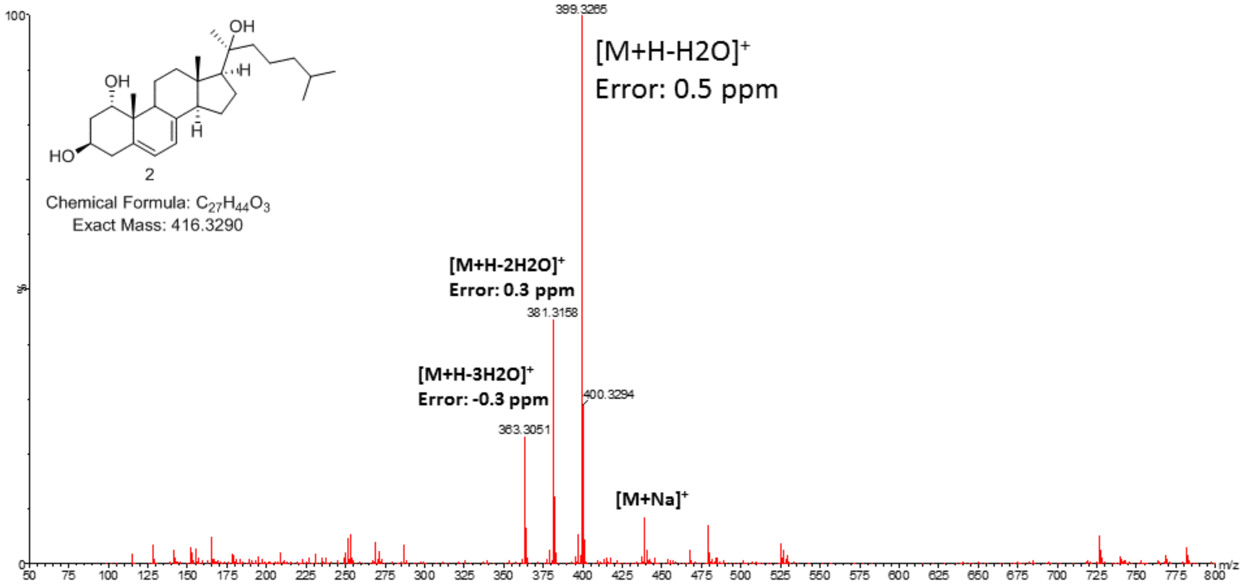

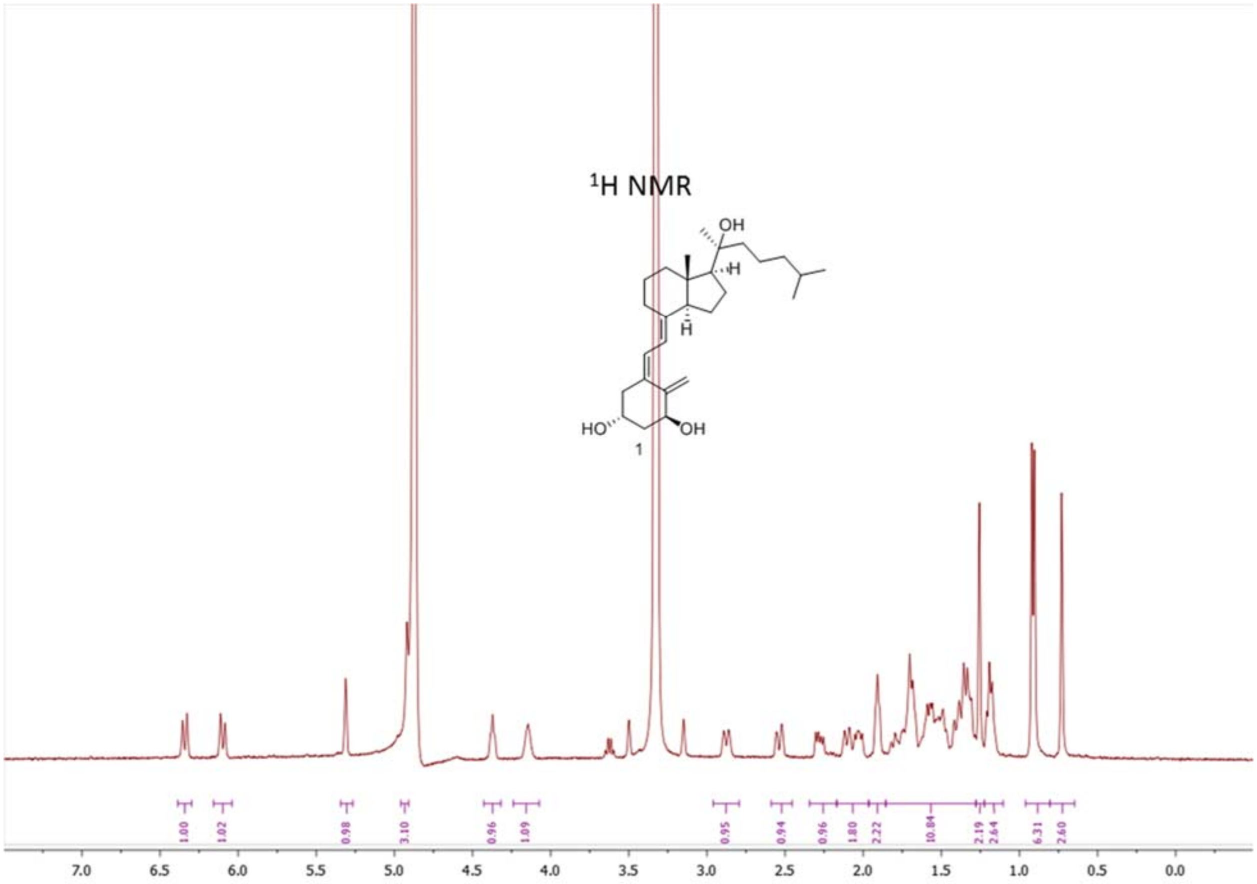

^{1}H NMR
OH
H
H
HO
OH
1
1.00
1.02
0.98
3.10
0.96
1.09
0.95
0.94
0.96
1.80
2.22
10.84
2.19
2.64
6.31
2.60
7.0
6.5
6.0
5.5
5.0
4.5
4.0
3.5
3.0
2.5
2.0
1.5
1.0
0.5
0.0

422

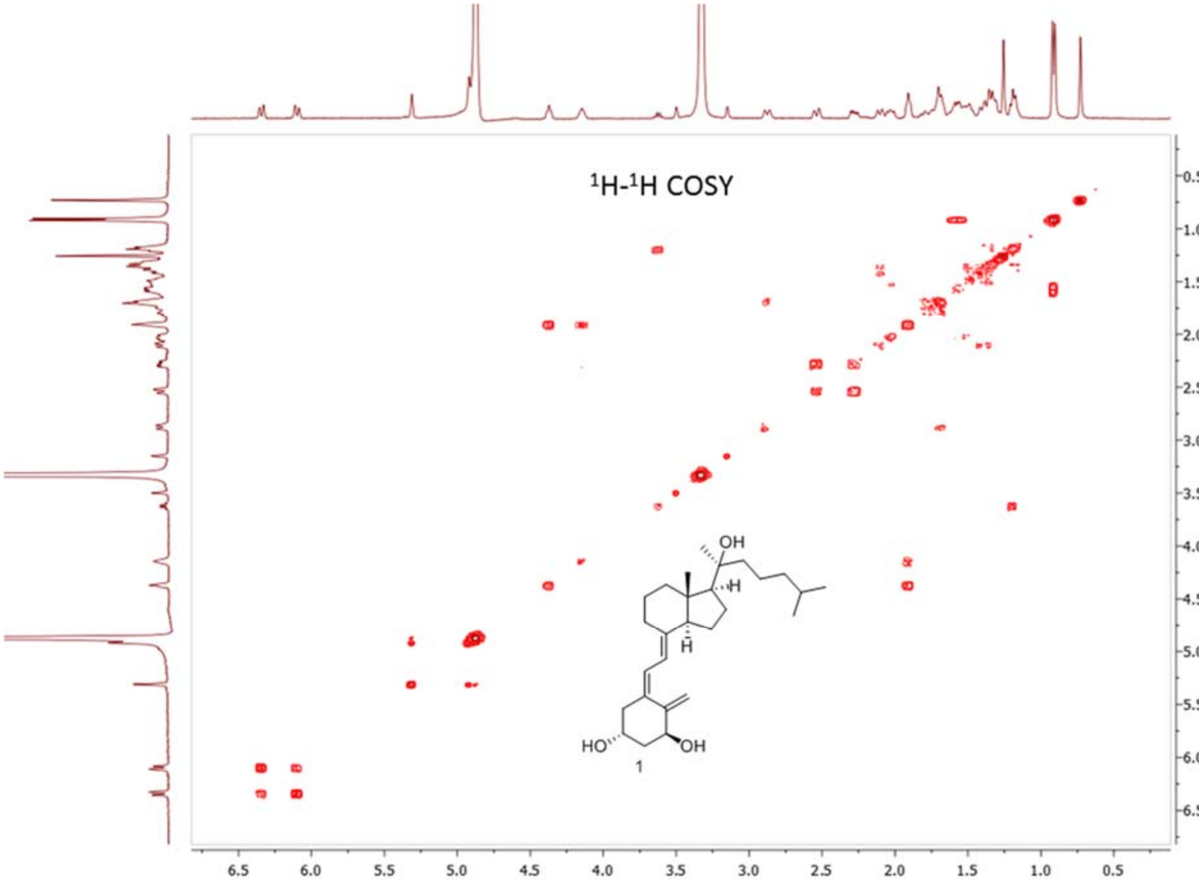

423

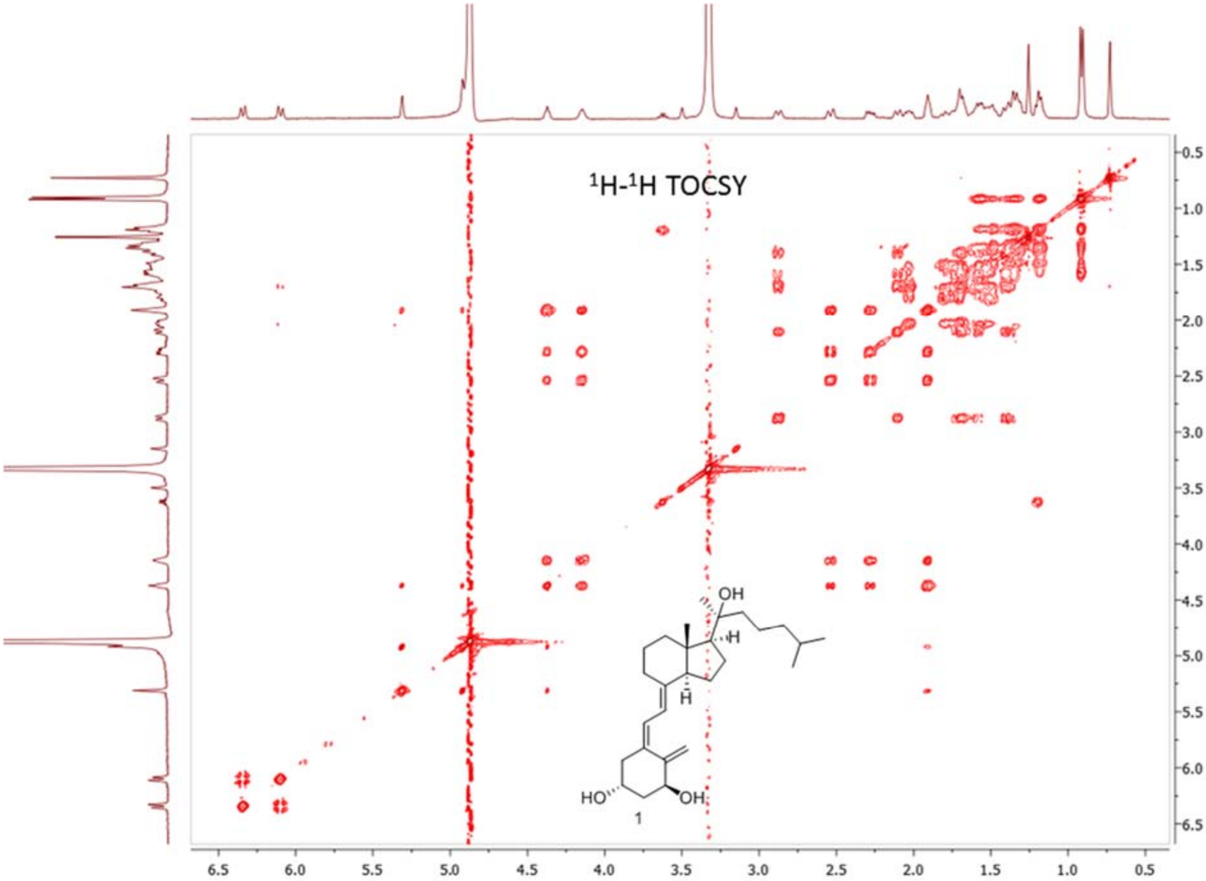

424

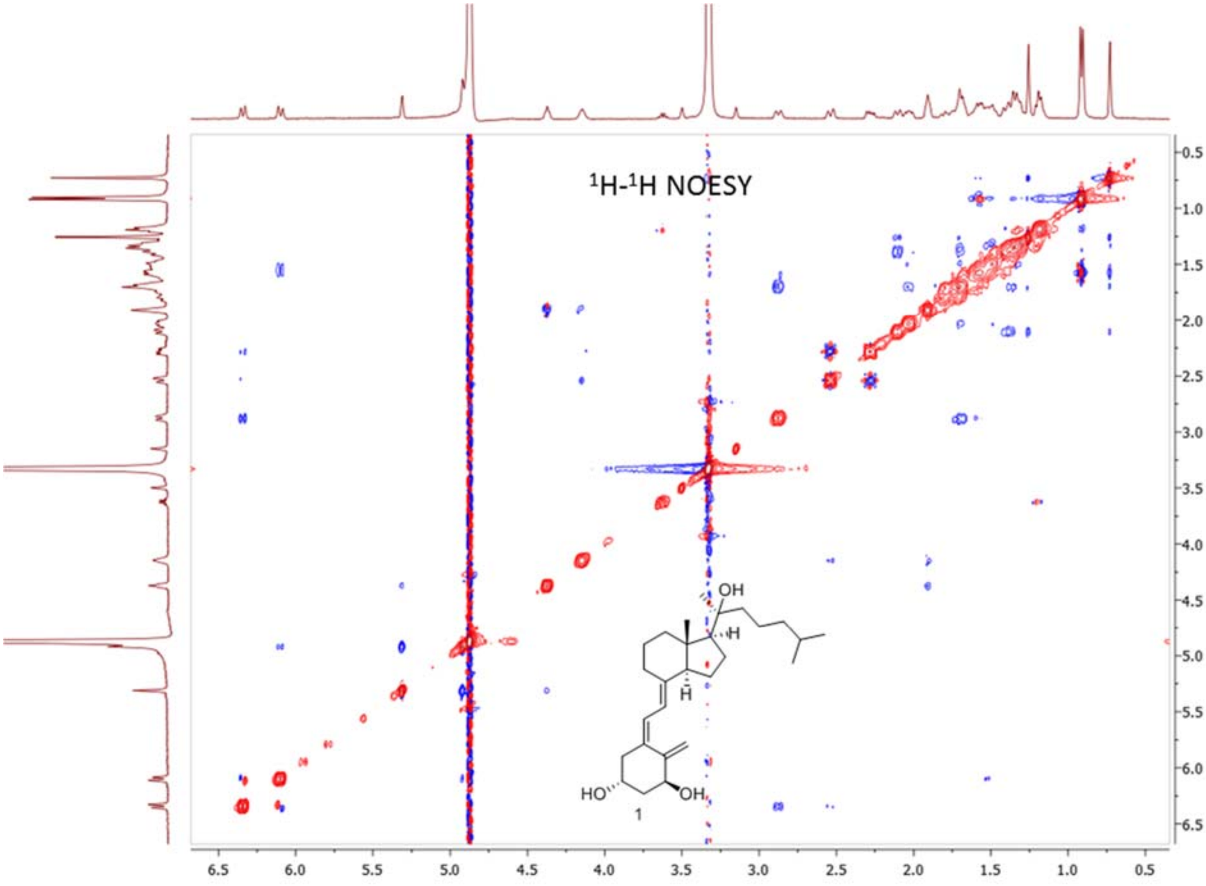

425

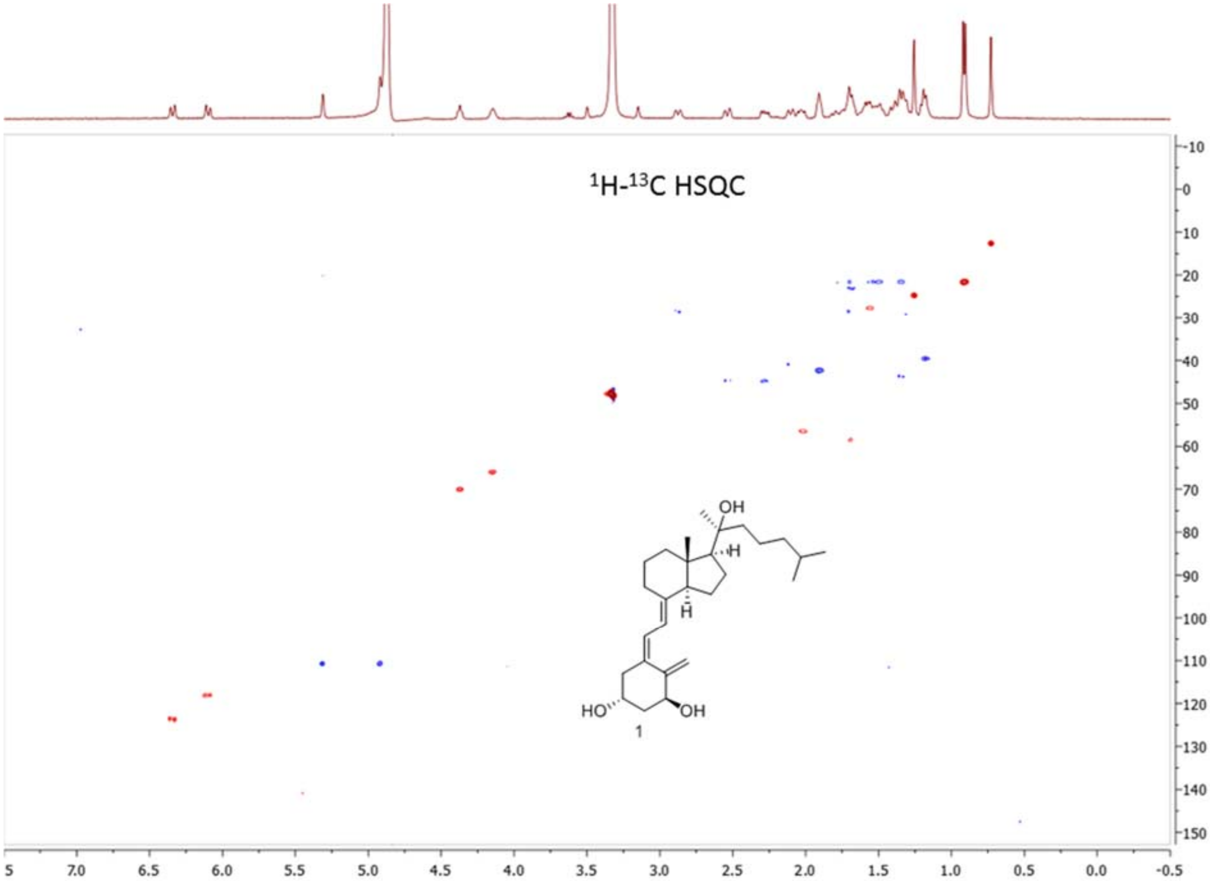

426

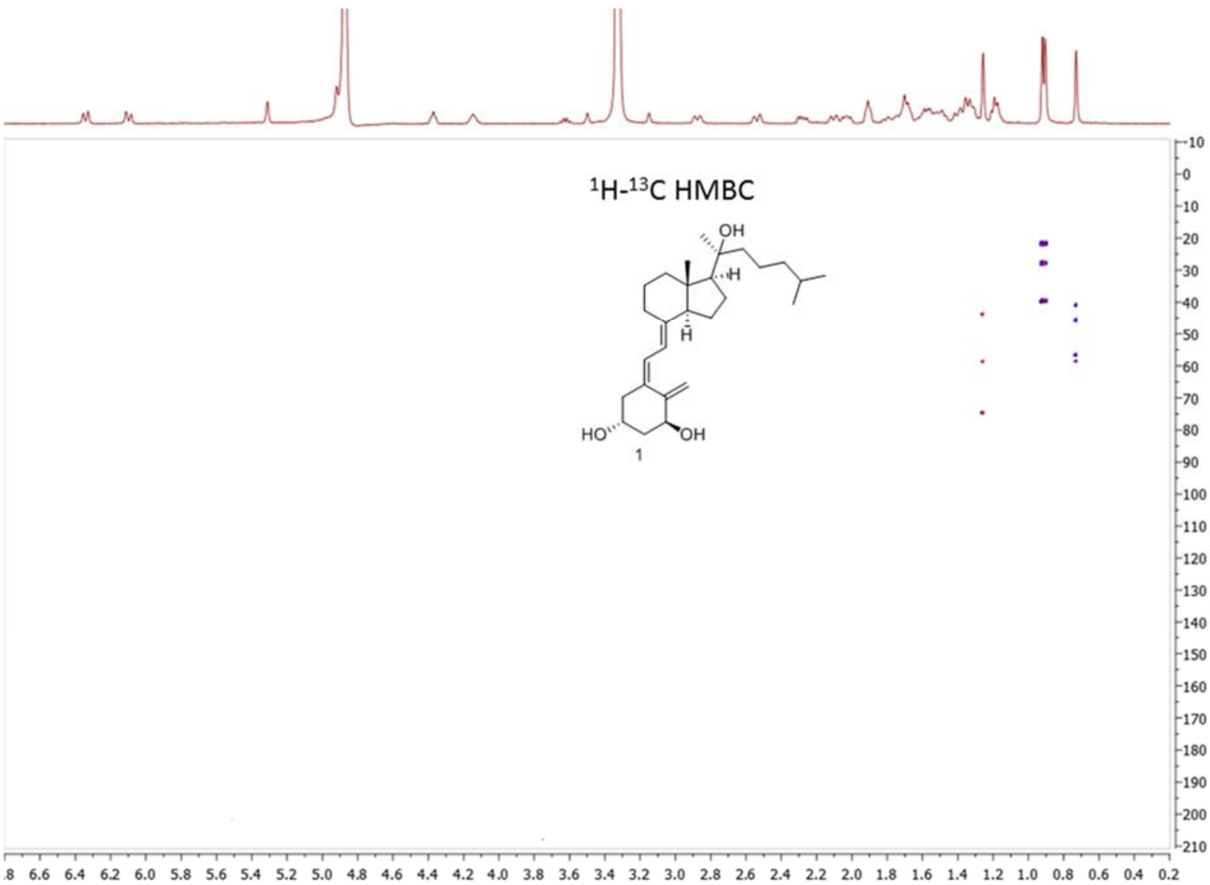

427

HPLC chromatogram

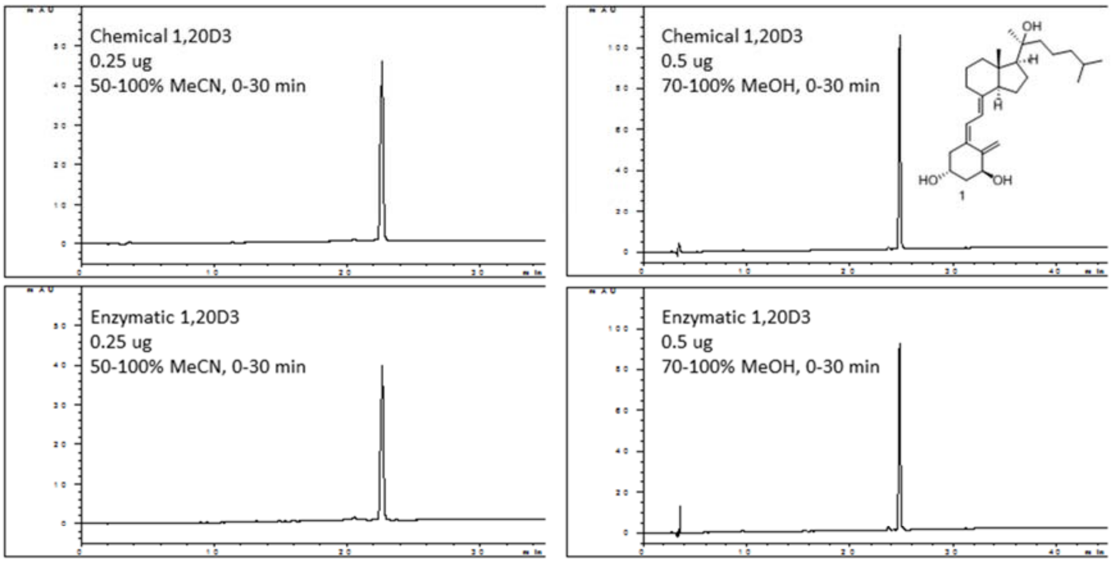

428

UV spectrum

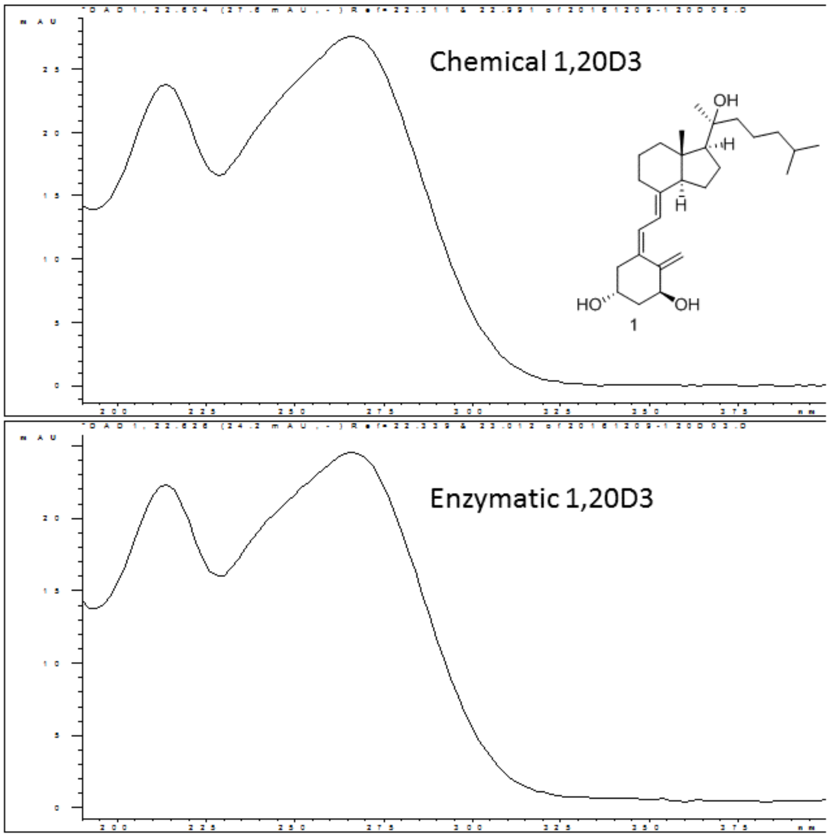

HPLC chromatogram

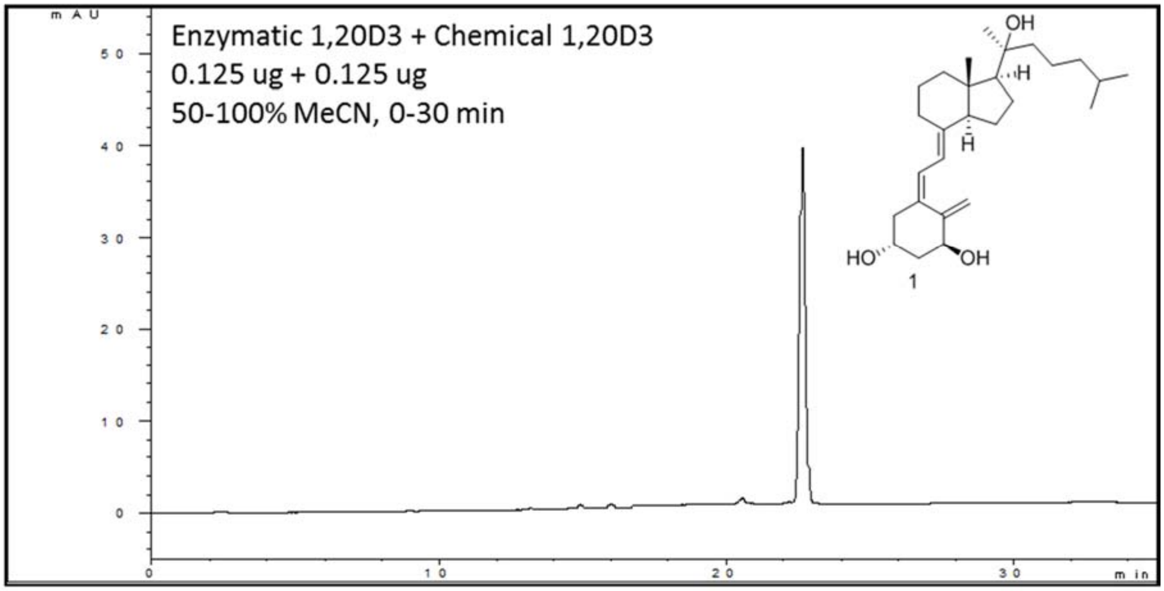

UV spectrum

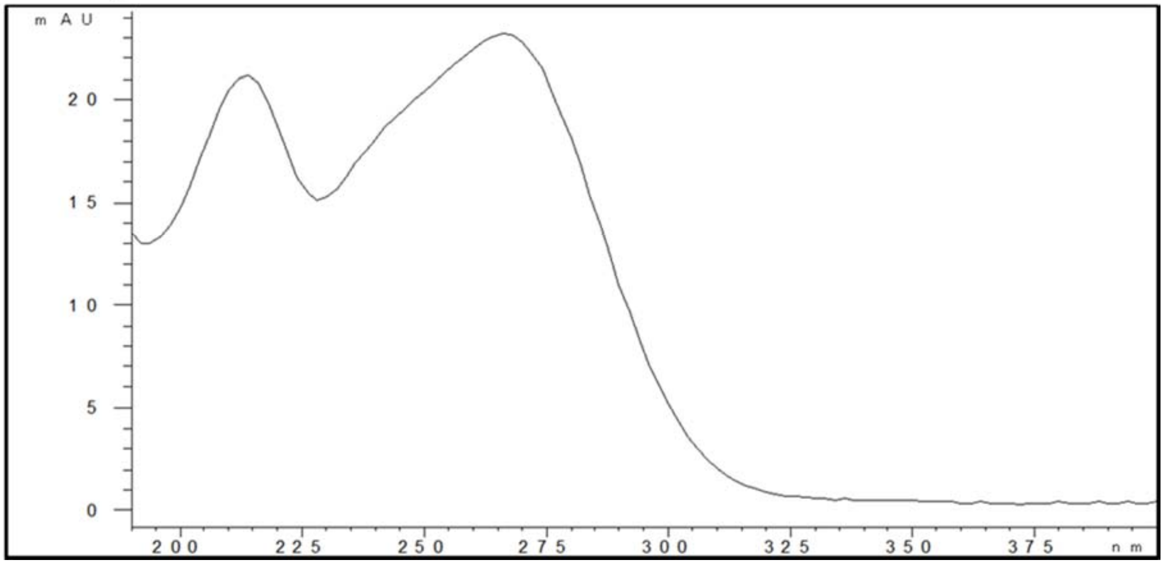

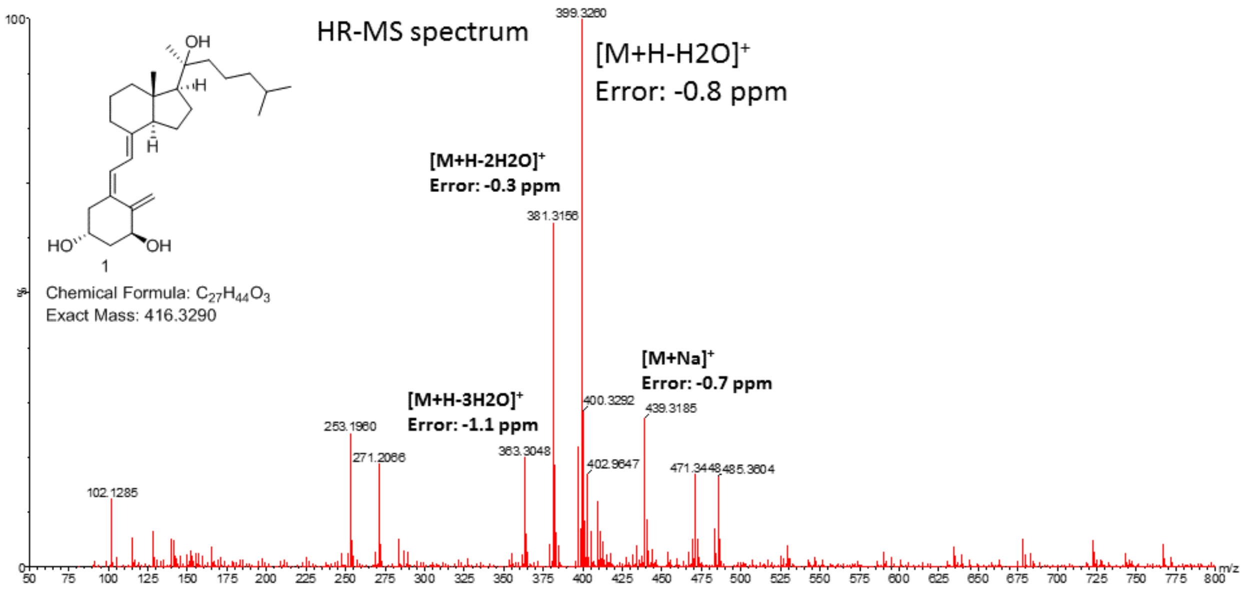
HR-MS spectrum
399.3260
[M+H-H2O]+
Error: -0.8 ppm
[M+H-2H2O]+
Error: -0.3 ppm
381.3156
[M+H-3H2O]+
Error: -1.1 ppm
363.3048
[M+Na]+
Error: -0.7 ppm
400.3292
439.3185
402.9647
471.3448
485.3604
253.1960
271.2066
102.1285
OH
H
H
HO
OH
1
Chemical Formula: C27H44O3
Exact Mass: 416.3290
100
%
0
m/z

431

6. checkCIF/PLATON report

Structure factors have been supplied for datablock(s) sj16_003

THIS REPORT IS FOR GUIDANCE ONLY. IF USED AS PART OF A REVIEW PROCEDURE FOR PUBLICATION, IT SHOULD NOT REPLACE THE EXPERTISE OF AN EXPERIENCED CRYSTALLOGRAPHIC REFEREE.

No syntax errors found. CIF dictionary Interpreting this report

Datablock: sj16_003

```
Bond precision:  C-C = 0.0058 A          Wavelength=1.54178

Cell:            a=12.3062(5)     b=6.4738(3)       c=19.5638(8)
                 alpha=90         beta=95.760(3)    gamma=90
Temperature:     100 K

                Calculated                 Reported
Volume          1550.74(11)                1550.73(11)
Space group     P 21                       P 21
Hall group      P 2yb                      P 2yb
Moiety formula  C31 H52 O5 Si              C31 H52 O5 Si
Sum formula     C31 H52 O5 Si              C31 H52 O5 Si
Mr              532.82                     532.81
Dx,g cm-3       1.141                      1.141
Z               2                          2
Mu (mm-1)       0.943                      0.943
F000            584.0                      584.0
F000’           586.06
h,k,lmax        14,7,23                    14,7,23
Nref            5727[ 3137]                5512
Tmin,Tmax       0.945,0.986                0.715,1.000
Tmin’           0.662

Correction method= # Reported T Limits: Tmin=0.715 Tmax=1.000
AbsCorr = NUMERICAL

Data completeness= 1.76/0.96      Theta(max)= 68.716

R(reflections)= 0.0596( 4649)     wR2(reflections)= 0.1280( 5512)

S = 1.054                  Npar= 335
```

The following ALERTS were generated. Each ALERT has the format
test-name_ALERT_alert-type_alert-level.
Click on the hyperlinks for more details of the test.

Alert level C

```
PLAT220_ALERT_2_C Non-Solvent Resd 1   C   Ueq(max)/Ueq(min) Range        3.6 Ratio
PLAT222_ALERT_3_C Non-Solvent Resd 1   H Uiso(max)/Uiso(min) Range        4.4 Ratio
PLAT340_ALERT_3_C Low Bond Precision on  C-C Bonds ...............    0.00579 Ang.
PLAT413_ALERT_2_C Short Inter XH3 .. XHn     H13A    ..  H24A    ..       2.11 Ang.
PLAT923_ALERT_1_C S    values in the CIF and FCF Differ by .......      0.011 Check
```

Alert level G

```
PLAT300_ALERT_4_G Atom Site Occupancy of *H24A   is Constrained at        0.5 Check
PLAT300_ALERT_4_G Atom Site Occupancy of *H24B   is Constrained at        0.5 Check
PLAT300_ALERT_4_G Atom Site Occupancy of *H24C   is Constrained at        0.5 Check
PLAT300_ALERT_4_G Atom Site Occupancy of *H24D   is Constrained at        0.5 Check
PLAT300_ALERT_4_G Atom Site Occupancy of *H24E   is Constrained at        0.5 Check
PLAT300_ALERT_4_G Atom Site Occupancy of *H24F   is Constrained at        0.5 Check
PLAT300_ALERT_4_G Atom Site Occupancy of *H30A   is Constrained at        0.5 Check
PLAT300_ALERT_4_G Atom Site Occupancy of *H30B   is Constrained at        0.5 Check
PLAT300_ALERT_4_G Atom Site Occupancy of *H30C   is Constrained at        0.5 Check
PLAT300_ALERT_4_G Atom Site Occupancy of *H30D   is Constrained at        0.5 Check
PLAT300_ALERT_4_G Atom Site Occupancy of *H30E   is Constrained at        0.5 Check
PLAT300_ALERT_4_G Atom Site Occupancy of *H30F   is Constrained at        0.5 Check
PLAT395_ALERT_2_G Deviating  X-O-Y   Angle from 120 Deg for  O8         127.7 Degre
PLAT791_ALERT_4_G The Model has Chirality at C9      (Chiral SPGR)          R Verify
PLAT791_ALERT_4_G The Model has Chirality at C11     (Chiral SPGR)          S Verify
PLAT791_ALERT_4_G The Model has Chirality at C14     (Chiral SPGR)          S Verify
PLAT791_ALERT_4_G The Model has Chirality at C15     (Chiral SPGR)          S Verify
PLAT791_ALERT_4_G The Model has Chirality at C20     (Chiral SPGR)          R Verify
PLAT791_ALERT_4_G The Model has Chirality at C26     (Chiral SPGR)          S Verify
PLAT791_ALERT_4_G The Model has Chirality at C31     (Chiral SPGR)          R Verify
PLAT791_ALERT_4_G The Model has Chirality at C33     (Chiral SPGR)          S Verify
PLAT791_ALERT_4_G The Model has Chirality at C36     (Chiral SPGR)          S Verify
PLAT910_ALERT_3_G Missing # of FCF Reflection(s) Below Theta(Min)         1 Note
PLAT912_ALERT_4_G Missing # of FCF Reflections Above STh/L=  0.600        3 Note
PLAT933_ALERT_2_G Number of OMIT Records in Embedded .res File ...        1 Note
PLAT978_ALERT_2_G Number C-C Bonds with Positive Residual Density.        2 Note
```

---

0 **ALERT level A** = Most likely a serious problem - resolve or explain
0 **ALERT level B** = A potentially serious problem, consider carefully
5 **ALERT level C** = Check. Ensure it is not caused by an omission or oversight
26 **ALERT level G** = General information/check it is not something unexpected

1 ALERT type 1 CIF construction/syntax error, inconsistent or missing data
5 ALERT type 2 Indicator that the structure model may be wrong or deficient
3 ALERT type 3 Indicator that the structure quality may be low
22 ALERT type 4 Improvement, methodology, query or suggestion
0 ALERT type 5 Informative message, check

It is advisable to attempt to resolve as many as possible of the alerts in all categories. Often the minor alerts point to easily fixed oversights, errors and omissions in your CIF or refinement strategy, so attention to these fine details can be worthwhile. In order to resolve some of the more serious problems it may be necessary to carry out additional measurements or structure refinements. However, the purpose of your study may justify the reported deviations and the more serious of these should normally be commented upon in the discussion or experimental section of a paper or in the "special_details" fields of the CIF. checkCIF was carefully designed to identify outliers and unusual parameters, but every test has its limitations and alerts that are not important in a particular case may appear. Conversely, the absence of alerts does not guarantee there are no aspects of the results needing attention. It is up to the individual to critically assess their own results and, if necessary, seek expert advice.

Publication of your CIF in IUCr journals

A basic structural check has been run on your CIF. These basic checks will be run on all CIFs submitted for publication in IUCr journals (*Acta Crystallographica*, *Journal of Applied Crystallography*, *Journal of Synchrotron Radiation*); however, if you intend to submit to *Acta Crystallographica Section C* or *E* or *IUCrData*, you should make sure that full publication checks are run on the final version of your CIF prior to submission.

Publication of your CIF in other journals

Please refer to the *Notes for Authors* of the relevant journal for any special instructions relating to CIF submission.

PLATON version of 24/11/2016; check.def file version of 23/11/2016

Datablock sj16_003 - ellipsoid plot

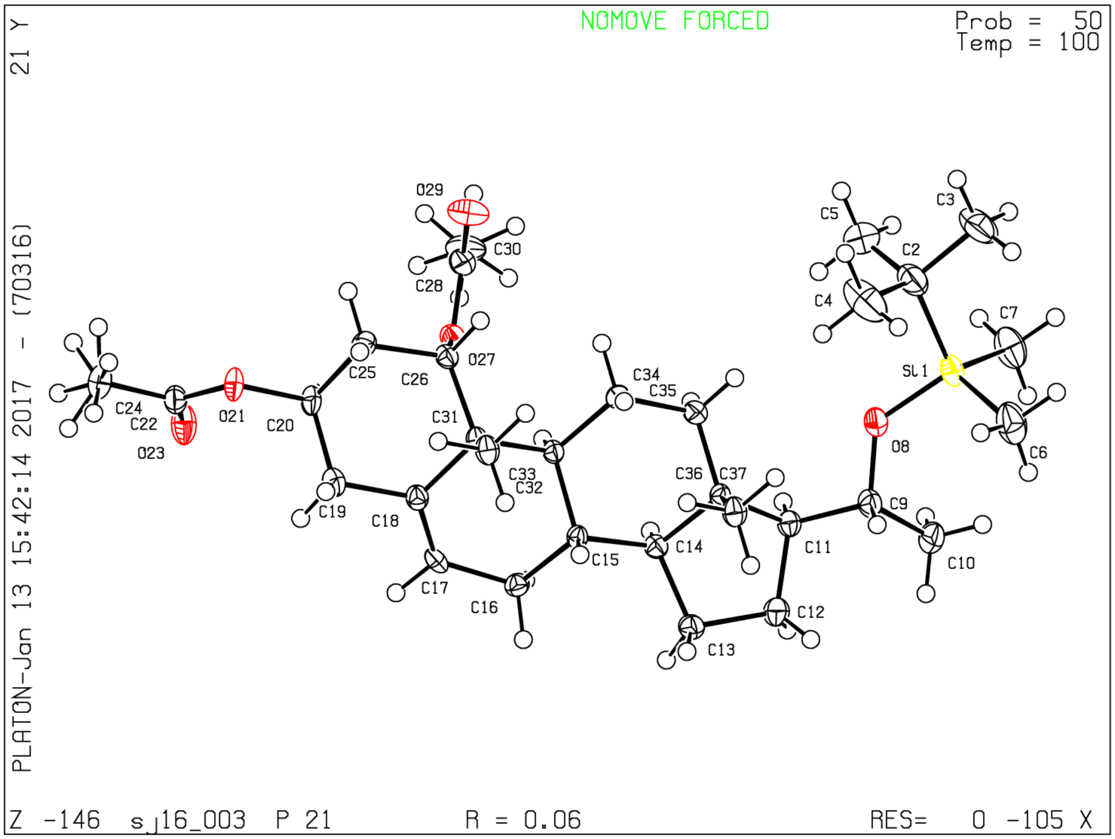

Supplement: Supplementary file 1 — Supplementary Info [file 41598_2017_10917_MOESM1_ESM.pdf]
